# Inhibitors of class I HDACs and of FLT3 combine synergistically against leukemia cells with mutant FLT3

**DOI:** 10.1007/s00204-021-03174-1

**Published:** 2021-10-19

**Authors:** Vanessa Wachholz, Al-Hassan M. Mustafa, Yanira Zeyn, Sven J. Henninger, Mandy Beyer, Melanie Dzulko, Andrea Piée-Staffa, Christina Brachetti, Patricia S. Haehnel, Andreas Sellmer, Siavosh Mahboobi, Thomas Kindler, Walburgis Brenner, Teodora Nikolova, Oliver H. Krämer

**Affiliations:** 1 Department of Toxicology, University Medical Center, Johannes Gutenberg University Mainz, Mainz, Germany; 2 Department of Zoology, Faculty of Science, Aswan University, Aswan, Egypt; 3 Department of Hematology, Medical Oncology and Pneumology, University Medical Center of the Johannes Gutenberg-University, Mainz, Germany; 4 German Consortia for Translational Cancer Research, Mainz, Germany; 5 Faculty of Chemistry and Pharmacy, Institute of Pharmacy, University of Regensburg, 93040 Regensburg, Germany; 6 Clinic for Obstetrics and Women’s Health, Johannes Gutenberg University Mainz, Mainz, Germany

**Keywords:** AML, Drug interaction, FLT3-ITD, HDAC, HDACi, TKi

## Abstract

Acute myeloid leukemia (AML) with mutations in the FMS-like tyrosine kinase (FLT3) is a clinically unresolved problem. AML cells frequently have a dysregulated expression and activity of epigenetic modulators of the histone deacetylase (HDAC) family. Therefore, we tested whether a combined inhibition of mutant FLT3 and class I HDACs is effective against AML cells. Low nanomolar doses of the FLT3 inhibitor (FLT3i) AC220 and an inhibition of class I HDACs with nanomolar concentrations of FK228 or micromolar doses of the HDAC3 specific agent RGFP966 synergistically induce apoptosis of AML cells that carry hyperactive FLT3 with an internal tandem duplication (FLT3-ITD). This does not occur in leukemic cells with wild-type FLT3 and without FLT3, suggesting a preferential toxicity of this combination against cells with mutant FLT3. Moreover, nanomolar doses of the new FLT3i marbotinib combine favorably with FK228 against leukemic cells with FLT3-ITD. The combinatorial treatments potentiated their suppressive effects on the tyrosine phosphorylation and stability of FLT3-ITD and its downstream signaling to the kinases ERK1/ERK2 and the inducible transcription factor STAT5. The beneficial pro-apoptotic effects of FLT3i and HDACi against leukemic cells with mutant FLT3 are associated with dose- and drug-dependent alterations of cell cycle distribution and DNA damage. This is linked to a modulation of the tumor-suppressive transcription factor p53 and its target cyclin-dependent kinase inhibitor p21. While HDACi induce p21, AC220 suppresses the expression of p53 and p21. Furthermore, we show that both FLT3-ITD and class I HDAC activity promote the expression of the checkpoint kinases CHK1 and WEE1, thymidylate synthase, and the DNA repair protein RAD51 in leukemic cells. A genetic depletion of HDAC3 attenuates the expression of such proteins. Thus, class I HDACs and hyperactive FLT3 appear to be valid targets in AML cells with mutant FLT3.

**Supplementary Information:**

The online version contains supplementary material available at 10.1007/s00204-021-03174-1.

## Introduction

The membrane-bound FMS-like tyrosine kinase-3 (FLT3) is activated by the FLT3 ligand and contributes to the development of hematopoietic cells. FLT3 is encoded on chromosome 13q12.2 and consists of five extracellular, immunoglobulin-like domains, a transmembrane domain, a juxtamembrane domain, and an intracellular tyrosine kinase domain (Majothi et al. 2020; Marensi et al. 2021; Scholl et al. 2020). Mutations in FLT3 promote cell growth and chemoresistance and lead to AML with poor prognosis. Internal tandem duplications in FLT3 (FLT3-ITD) occur in 20–35% of patients with de novo AML and tyrosine kinase domain (FLT3-TKD) mutations occur in 5–10% of patients with de novo AML (Heidel et al. 2006; Majothi et al. 2020; Marensi et al. 2021; Scholl et al. 2020).

Tyrosine kinase inhibitors (TKi) against hyperactive FLT3 show promising clinical results (Majothi et al. 2020; Marensi et al. 2021; Perl 2019; Scholl et al. 2020). However, during the therapy of AML patients with AC220 (quizartinib), which has a narrow inhibition profile (Zarrinkar et al. 2009), FLT3-TKD mutations arise. These disfavor the binding of AC220 (Smith et al. 2012). FLT3-TKD mutations can also adopt conformations that are unfavorable for the binding of broad-acting TKi. For example, the FLT3-N676K mutation disables binding of midostaurin (PKC412) (Heidel et al. 2006), which is approved for the treatment of AML with mutant FLT3 (Majothi et al. 2020; Perl 2019). A newly approved TKi against aberrant FLT3 is the dual FLT3/AXL inhibitor gilteritinib which is far less specific than AC220 (Pulte et al. 2021). We have recently developed the new FLT3 inhibitor (FLT3i) marbotinib which has nanomolar activity against FLT3-ITD, a very narrow inhibitory profile, and activity against FLT3-TKD mutants (Sellmer et al. 2020).

An alternative route to target FLT3 mutants is the induction of their degradation by ubiquitination-mediated proteasomal and lysosomal pathways. Histone deacetylase inhibitors (HDACi) are epigenetic drugs that induce a proteasomal degradation of FLT3-ITD. The underlying mechanism is a transcriptional induction of the E2 ubiquitin conjugase UBCH8 and a phosphorylation-dependent binding of FLT3-ITD by the UBCH8-associated SIAH1/SIAH2 E3 ubiquitin ligases (Buchwald et al. 2013). The proteasomal degradation of FLT3-ITD in the presence of HDACi correlates with a decreased interaction of FLT3-ITD with the chaperone HSP90 (Bali et al. 2004; Nishioka et al. 2008). Combined pharmacological inhibition of HDACs and FLT3-ITD synergistically induces apoptotic cell death in AML cells (Bali et al. 2004; Li et al. 2017; Pietschmann et al. 2012). This physiologically important cell death mechanism is controlled by pro- and anti-apoptotic proteins and dismantles cells without debris (Kale et al. 2018). Apoptosis of FLT3-ITD positive cells that are exposed to FLT3i and HDACi is linked to a proteasomal and caspase-dependent degradation of FLT3-ITD (Bali et al. 2004; Buchwald et al. 2013; Li et al. 2017; Nishioka et al. 2008; Pietschmann et al. 2012).

The HDAC family comprises classes I–IV (Krämer 2009; Krämer et al. 2014) and four HDACi have been approved by the FDA for the treatment of lymphoma and myeloma. These include the hydroxamic acid derivatives vorinostat (SAHA), panobinostat (LBH589), belinostat (PXD-101), and the depsipeptide FK228 (romidepsin/FR901228/istodax) (Li et al. 2019b). It is unclear whether a pharmacological inhibition of individual classes of HDACs can interact favorably with specific TKi against leukemic cells with aberrant FLT3 activation. Due to the strong association between class I HDACs and tumor growth and development (Li et al. 2020; Luo and Li 2020), these could be good targets for such an approach. Class I HDACi with reported anticancer activity are the clinically tested entinostat (MS-275), the neurologically used drug valproic acid, and the experimental agents RGFP966 and MERCK60 (Beyer et al. 2019; Blaheta and Cinatl 2002; Krämer 2009; Lakshmaiah et al. 2014).

Beyond the activation of proliferative kinase signaling cascades, mutant FLT3 promotes the expression of DNA repair proteins that antagonize chemotherapy. For example, FLT3-ITD and FLT3-TKD induce the expression of the filament protein RAD51, which supports faithful DNA repair through homologous recombination (HR) (Bagrintseva et al. 2005; Seedhouse et al. 2006). Checkpoint kinases (CHKs) are primary sensors of DNA replication stress and damage, and inhibitors of these enzymes kill AML cells with hyperactive FLT3 (Boudny and Trbusek 2020; Yuan et al. 2014). HDACs and HDACi modulate the expression and the acetylation of DNA repair proteins, and thereby modulate the threshold for lethal DNA damage in cancer cells with chemotherapy-induced stress (Göder et al. 2018; Krumm et al. 2016; Nikolova et al. 2017). In permanent and primary human AML cells with and without FLT3-ITD, HDACi decrease RAD51, CHK1, and the cell cycle regulatory kinase WEE1 (Dai et al. 2013; Li et al. 2019a; Qi et al. 2015; Xie et al. 2013; Zhao et al. 2017; Zhou et al. 2015). Whether a combined application of FLT3i and HDACi alters the levels of DNA repair proteins is not known.

HDACi with a specific inhibitory profile against individual HDACs produce less side effects than pan-HDACi (Li et al. 2020; Luo and Li 2020; Müller and Krämer 2010) and could therefore be a good combination partner with FLT3i. We treated AML cells that carry FLT3-ITD with AC220 and HDACi. We used the clinically approved HDACi FK228, which preferentially inhibits class I HDACs (Pojani and Barlocco 2020), and the benzamide-based HDACi RGFP966, which preferentially inhibits HDAC3 (Beyer et al. 2019; Matthews et al. 2015). We analyzed drug-induced apoptosis and replication stress/DNA damage. Combinations of nanomolar doses of AC220 or marbotinib with nanomolar doses of FK228 or micromolar doses of RGFP966 synergistically induced apoptosis and altered cell cycle progression. This cytotoxicity was associated with a loss of survival signaling through FLT3-ITD, a dysregulation of cell cycle and DNA repair proteins, and an accumulation of markers for replication stress and DNA damage.

## Materials and methods

### Cell lines and reagents

Cells were a gift from Prof. Dr. Frank-Dietmar Böhmer, University of Jena, Germany or were from the group of Prof. Dr. Thomas Kindler, Mainz, Germany (cells were originally from the German collection of microorganisms and cell cultures, Braunschweig, Germany). Cell authentication was done by DNA-profiling using eight different, highly polymorphic short tandems repeats (MV4-11, RS4-11, and K562 cells). Cells were maintained at 37 °C and 5% CO_2_ in humidified atmosphere. Growth media was RPMI-1640 medium containing 10% fetal calf serum (FCS, Lonza, Cologne, Germany) and 1% penicillin/streptomycin (Sigma, Munich, Germany). We used no commonly mischaracterized cell lines. Moreover, cells were confirmed to be free of mycoplasma every 1–4 months. The p53 status of the cells is wild-type for MV4-11 cells (Yan et al. 2020) and MOLM-13 cells (Thompson et al. 2010), mutant for RS4-11 cells (Demir et al. 2020), null for K562 cells (Pons et al. 2021). Marbotinib has been patented (Mahboobi et al. 2019; Sellmer et al. 2020). MV4-11 cells with an inducible shRNA for HDAC3 were engineered by lentiviral transduction with the Tet-on^®^-inducible lentiviral vector pTRIPZ (Thermo Scientific) encoding the miR-shRNA shHDAC3, clone Id: V3THS_380877; *AGAAGTCCACTACCTGGTT*. Lentiviral particles were produced by co-transfection of 293FT cells with the psPAX2, pMD2.G, and the lentiviral shRNA HDAC3 vector. Transfections were carried out using TransIT^®^ (Mirus) as per the manufacturer’s instructions in the presence of 5 μg/mL polybrene. Following transduction, cells were selected with 1.0 μg/mL puromycin (Sigma-Aldrich).

### Flow cytometry

For viability measurements using DAPI, cells were resuspended and incubated for 5 min at room temperature in PBS containing 2 mM EDTA and 1 µg/mL DAPI. Cell cycle staining was performed using hypotonic fluorochrome solution (HFS) buffer (3.88 mM sodium citrate, 0.1% (v/v) triton X-100) containing 50 mM PI. Cells were incubated for 15 min in HFS-buffer prior measurement. Cell cycle, subG1 fraction, and annexin-V/propidium iodine (PI) analyses were done according to our recent protocols, see (Beyer et al. 2019; Kiweler et al. 2018, 2020; Pons et al. 2018). Flow cytometry analyses were performed with a FACS Canto II (BD Bioscience, Heidelberg, Germany). FACSDiva 7.0 and FlowJo were used as software tools to evaluate the flow cytometry data.

### Synergy calculation

Synergism was determined using a free software that was designed by the Cancer Research Institute Cambridge, UK. The drug interaction models highest single agent (HSA), Loewe, and Bliss were used to determine synergism scores. Scores are interpreted as antagonistic effects: *x* ≤ − 10; no interaction effects: − 10 ≤ *x* ≤ 10; synergistic effects: *x* ≥ 10.

### Protein analyses and immunofluorescence staining for histone H2AX with phosphorylated S139 (ɣH2AX)

Immunoblot and immunofluorescence analyses were carried out as described by us recently (Beyer et al. 2019; Pons et al. 2018). The antibodies that we used are listed in Table 1.

**Table 1:** List of antibodies used in this study

Antibody	Order number	Company
ac-H4 (Lys5)	#86475	Cell Signaling
ac-H3 (Lys14)	06-599	Millipore
ac-tubulin (Lys40)	T7451	Sigma
β-actin	sc-47778	Santa Cruz
CHK1	#2360	Cell Signaling
p-CHK1 (Ser345)	#2344	Cell Signaling
cleaved caspase-3	#9661	Cell Signaling
ERK1	sc-271269 or #271269	Santa Cruz Cell Signaling
p-ERK1(Thr202)/p-ERK2 (Tyr204)	#9101	Cell Signaling
FLT3	sc-480	Santa Cruz
p-FLT3	#3461	Cell Signaling
HSP90	sc-13119	Santa Cruz
ɣH2AX	#9718	Cell Signaling
PCNA	sc-56	Santa Cruz
p21 (CIP1/WAF1)	ab109520	Abcam
P53	sc-126	Santa Cruz
RAD51	ab6380	Abcam
p-RPA (Thr21)	ab661065	Abcam
STAT5	sc-74442 or BD616191	Santa Cruz BD Biosciences
p-STAT5 (Tyr694)	ma5-14973	Thermo Fisher
thymidylate synthase	#9045	Cell Signaling
α-tubulin	Ab176560	Abcam
UBCH8	AP2118a	Abgent
Vinculin	sc-7336	Santa Cruz
WEE1	sc-5285	Santa Cruz

### Detection of replicating (S phase) cells by 5-ethynyl-2′-deoxyuridine (EdU) incorporation

EdU incorporation for the detection of S phase cells was performed using the EdU Click-It Imaging Kit (Thermo Fisher Scientific) according to the manufacturer’s instructions, with slight modifications. We used the FAM azide click-it dye from Lumiprobe (Hannover, Germany), as described in (Berte et al. 2016). EdU was given to the cell cultures at a final concentration of 10 µM. After 1 h incubation in the incubator (light-protected), cells were washed in PBS and fixed for 10 min onto coverslips at − 20 °C in an ice-cold methanol:aceton mixture (7:3, v/v). Cells were washed and rehydrated in PBS, and EdU detection was performed according to the manufacturer. Nuclei were counterstained with TO-PRO3 (Invitrogen), which was added to the antifade medium Vectashield (Vector Labs). Representative confocal images were captured by the ZEN2009 software for a laser scanning microscope LSM710 (Carl Zeiss, Germany). Cells with the Click-It fluorescent dye bound to EdU in the DNA were counted on LSM images, using the ImageJ software (version 1.51M9).

## Results

### Synergistic cytotoxic interaction of AC220 and FK228

**Fig. 1:**
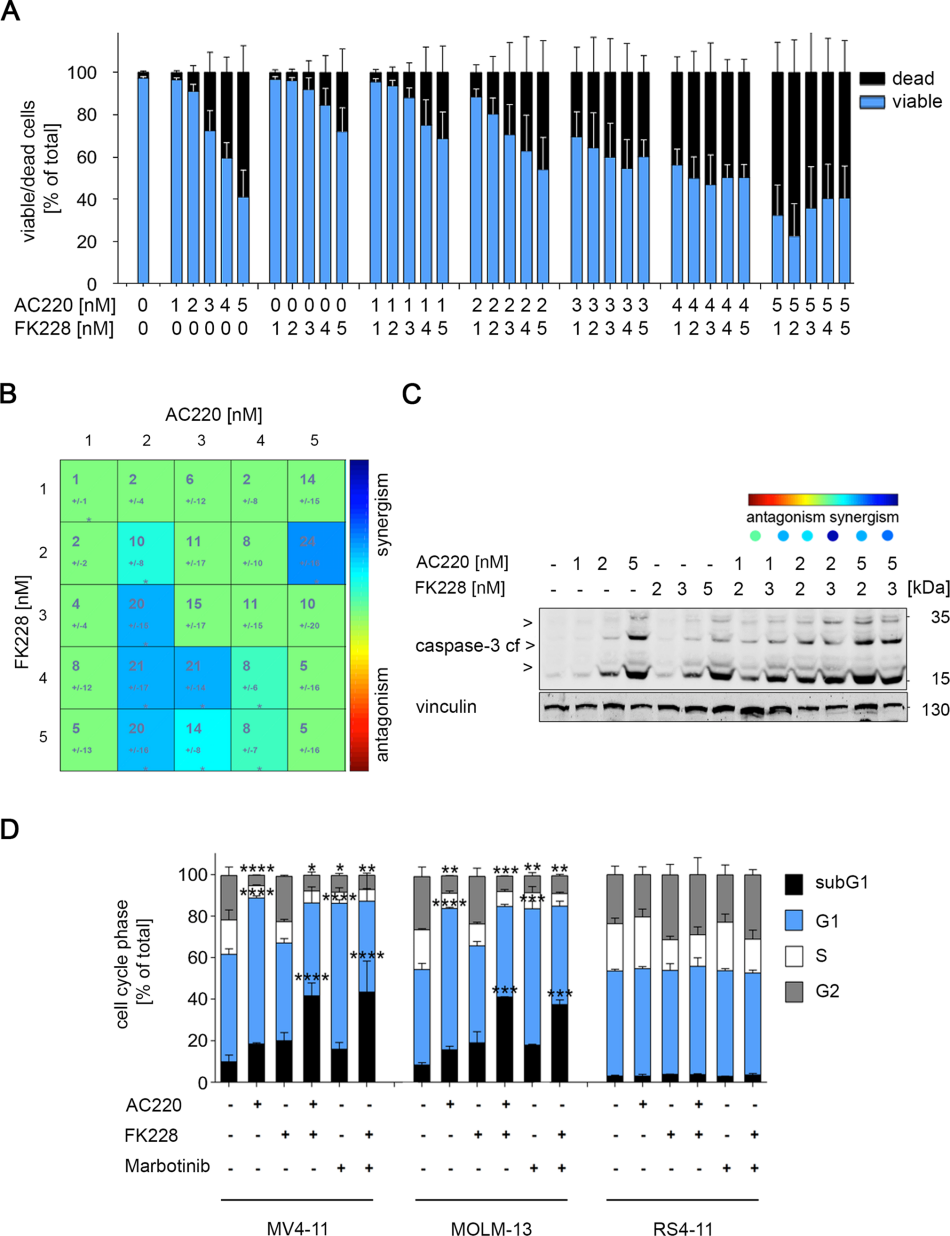
FLT3i and FK228 synergistically increase cell death of human leukemic cells with FLT3-ITD. **a** MV4-11 cells were treated for 24 h with either AC220 and/or FK228 in doses as indicated. Cells were then stained with DAPI and analyzed by flow cytometry for DAPI incorporation. Bars represent the mean ± SD; *n* = 6. **b** Determination of synergistic mechanism by combining AC220 and FK228 treatment in MV4-11. Synergistic effects were determined by using combenefit software. Using the HSA model, we determined the mean synergistic scores. Calculated scores determine drugs interaction as followed (antagonistic effects: *x* ≤ − 10; no interaction effects: − 10 ≤ *x* ≤ 10; synergistic effects: *x* ≥ 10). Results represent the mean ± SD (*n* = 6; HSA). **c** MV4-11 cells were incubated for 24 h with either AC220 and/or FK228 in doses as indicated. Immunoblot analysis of whole cell lysates was performed to detect cleaved caspase-3; vinculin served as loading control (*n* = 3). **d** MV4-11 cells, MOLM-13 cells, and RS4-11 cells were treated with FK228, AC220, or marbotinib for 24 h. Cells were then subjected to flow cytometry analyses for cell cycle distribution and DNA fragmentation (*n* = 3; −, untreated; + , treated with 2 nM of the above-mentioned drugs)

We incubated human AML cells with FLT3-ITD (MV4-11 cells, from a biphenotypic B myelomonocytic leukemia in a 10-year-old male child) with 1–5 nM AC220 and 1–5 nM FK228 for 24 h (36 conditions, 25 combinations of AC220 plus FK228). Tests for cell viability, by flow cytometry for DAPI incorporation as a sign of cytotoxicity, demonstrated that doses starting from 2 nM AC220 and 3 nM FK228 as well as 3 nM AC220 plus 1 nM FK228 and 1 nM FK228 plus 2 nM AC220 induced cell death (Fig. 1A).

We used the HSA algorithm to investigate whether the cytotoxic effects of AC220 and FK228 against MV4-11 cells are synergistic. We noted that 5 of the 25 combinations synergistically induced DNA-fragmentation. The remaining combinations caused additive cytotoxic effects and no antagonistic effects occurred. 2 nM AC220 and 3 nM FK228 were the lowest drug concentrations that interacted synergistically against MV4-11 cells (Fig. 1B).

Caspase-3 is activated by limited proteolysis in apoptotic cells and cleaves key survival proteins (Boice and Bouchier-Hayes 2020). Immunoblot analyses verified that cleaved caspase-3 accumulated dose-dependently in MV4-11 cells that were incubated with 1, 2, and 5 nM AC220 and 3 and 5 nM FK228. Cells that were treated with synergistically active combinations of AC220 plus FK228, accumulated higher levels of cleaved caspase-3 than cells that were treated with the single drugs (Fig. 1C).

Next, we assessed the impact of AC220 and FK228 on cell cycle alterations and apoptosis-associated DNA cleavage by flow cytometry. Depending on cell cycle progression, cells have increased DNA content and subsequently increased incorporation of PI. Cells with reduced DNA content are a hallmark of apoptosis and stem from the activation of the caspase-activated DNase CAD. Such cells accumulate as subG1 fraction in flow cytometry analyses (Marx-Blümel et al. 2017). We noted that 2 nM AC220 stalled MV4-11 cells in G1 phase and reduced the number of cells in the S and G2/M phases. 2 nM FK228 reduced the G1 and S phase populations. Combination of the drugs significantly increased the subG1 fraction populations and decreased cells in G2/M phase (Fig. 1D). We observed these cell cycle alterations and the induction of the subG1 fractions similarly in MOLM-13 cells (express FLT3-ITD and wild-type FLT3, from a 20-year-old man with AML cells after initial myelodysplastic syndrome) that we treated with 2 nM AC220 ± 2 nM FK228 (Fig. 1D).

To control whether these cytotoxic effects are specific for AML cells with mutant FLT3, we incubated human leukemic cells with wild-type FLT3 (RS4-11 cells from a 32-year-old woman with acute lymphoblastic leukemia) with FK228 and AC220. Flow cytometry showed that FK228 increased the number of RS4-11 cells in G2/M phase, without an increase of cells in the subG1 phase. These data confirm that RS4-11 cells have a lower sensitivity to HDACi than MV4-11 cells and MOLM-13 cells (Buchwald et al. 2013). Also as expected for a cell line with wild-type FLT3 (Zarrinkar et al. 2009), AC220 had no discernable effect on RS4-11 cells. The combination of FK228 and AC220 did not increase the effects of FK228 (Fig. 1D). We validated our flow cytometry technology with RS4-11 cells that were treated with hydroxyurea. Hydroxyurea induced apoptosis in RS4-11 cells (Supplementary Fig. S1), verifying that the low levels of apoptosis induction by AC220 plus FK228 (Fig. 1D) are not due to a technical limitation.

To corroborate these data with another FLT3i, we treated the three leukemic cell types with 2 nM FK228 and 2 nM marbotinib (Sellmer et al. 2020). As seen with AC220 plus FK228, combinations of FK228 and marbotinib were not toxic for RS4-11 cells (Fig. 1D). Similar as AC220, marbotinib decreased the numbers of cells in S phase and G2/M phase. In MV4-11 cells and MOLM-13 cells, marbotinib plus FK228 induced cytotoxic DNA fragmentation significantly and more effectively than the single drugs (Fig. 1D).

These data illustrate that AC220 and FK228 are low nanomolar inducers of cell death in FLT3-ITD positive leukemic cells and that combinations thereof are superior to the single treatments.

### AC220 and FK228 induce different types of cell cycle arrest

**Fig. 2:**
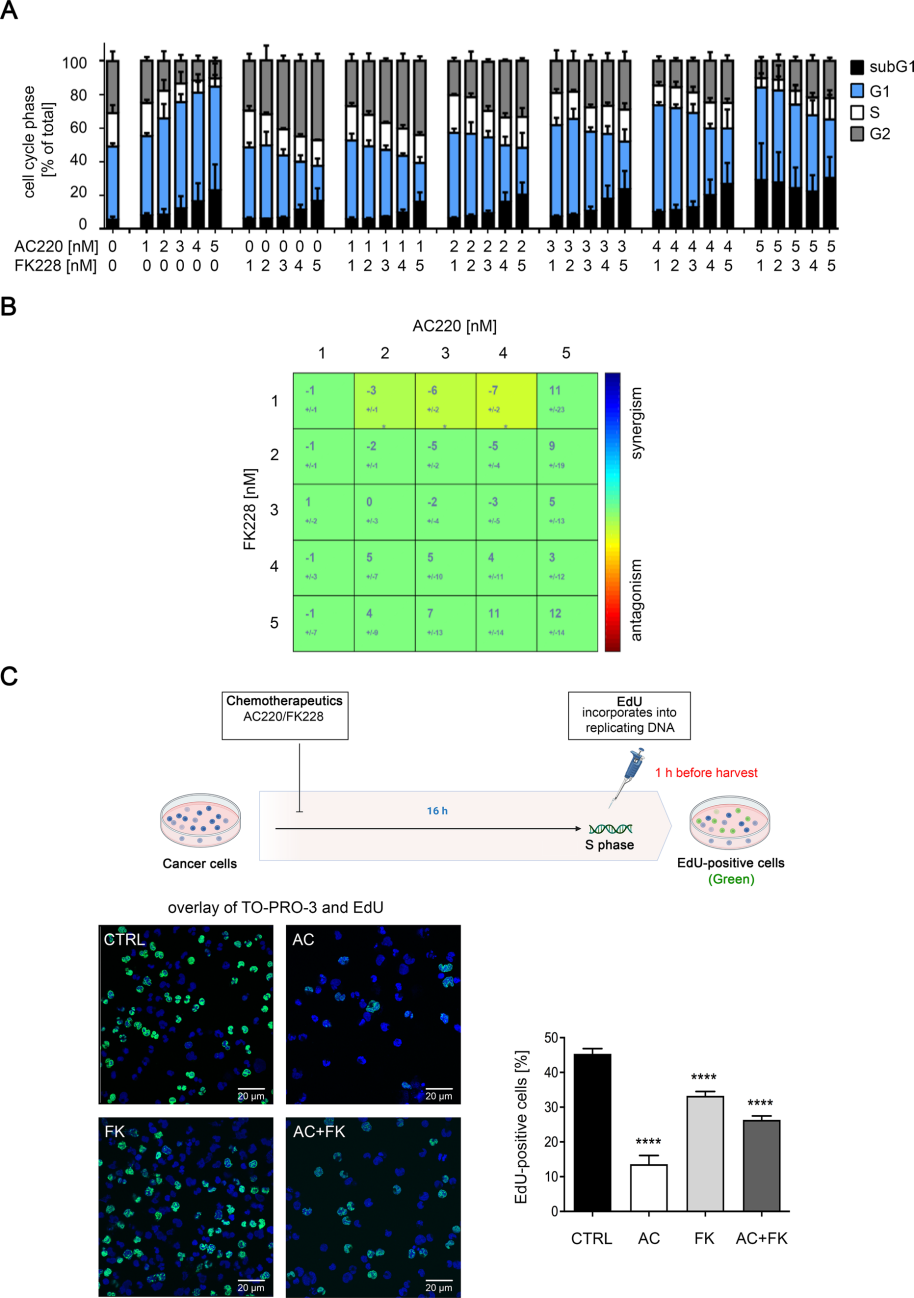
FLT3i and HDACi dysregulate cell cycle progression and DNA replication. **a** MV4-11 cells were treated with single and combined doses of AC220 and FK228 for 16 h and analyzed for cell cycle distribution. Bars show mean ± SD (*n* = 6). **b** Lack of synergistic mechanism by combining AC220 and FK228 for 16 h in MV4-11**.** Effects were determined using combenefit software and the HSA model. Calculated scores determine drugs interaction as followed (antagonistic effects: *x* ≤ − 10; no interaction effects: − 10 ≤ *x* ≤ 10; synergistic effects: *x* ≥ 10). Results represent the mean ± SD (*n* = 3). **c** We analyzed EdU incorporation as indicated in the scheme. We treated MV4-11 cells for 16 h with 2 nM AC220 ± 3 nM FK228. Cells were pulse labeled with 10 µM EdU for 1 h before harvest. Cells were counterstained using TO-PRO3; scale bar represents 20 µm. Images are representative for three independent experiments with a similar outcome. The graphs show the EdU-positive cells; ****p < 0.0001

Since the high rates of cell death induction in AC220 plus FK228 treated MV4-11 cells (Fig. 1A–C) may blur drug-induced cell cycle effects, we tried to identify time points at which the drugs produce cell cycle alterations without excessive killing. Flow cytometry showed that after 16 h-exposures, most single and combinatorial drug applications caused less than 20% accumulation of subG1 fractions in MV4-11 cell cultures (Fig. 2A) and there were no synergistic cytotoxic drug effects (Fig. 2B).

We additionally analyzed cell cycle effects of the drugs by the analysis of the incorporation of the thymidine analog EdU, which occurs during S phase (Fig. 2C). Immunofluorescence demonstrated EdU incorporation in 45% of untreated MV4-11 cells. Cells treated with 3 nM AC220 for 16 h incorporated significantly less EdU, reaching 14% EdU-positive cells (Fig. 2C). This is consistent with the observed G1 phase arrest of MV4-11 cells that are exposed to AC220 (Fig. 2A). Upon FK228 treatment, we noted that 33% of MV4-11 cells incorporated EdU but also larger cells with a particularly bright EdU staining (Fig. 2C). The occurrence of such cells corresponds to an increase in cells that are in G2/M phase after S phase upon the treatment with FK228 (Fig. 2A). In the cotreatment scheme, we observed 26% of EdU-positive MV4-11 cells (Fig. 2C), corresponding to the differing effects of AC220 and FK228 on cell cycle arrest in G1 phase or G2/M phase (Fig. 2A).

We conclude that AC220 and FK228 have opposite effects on cell cycle progression, with AC220 inducing a G1 phase arrest and FK228 inducing a G2/M phase arrest. Combinations of these drugs dose-dependently antagonize the cell cycle arrests that the single drugs cause.

### AC220 and FK228 promote their target engagement and attenuate DNA repair proteins

**Fig. 3:**
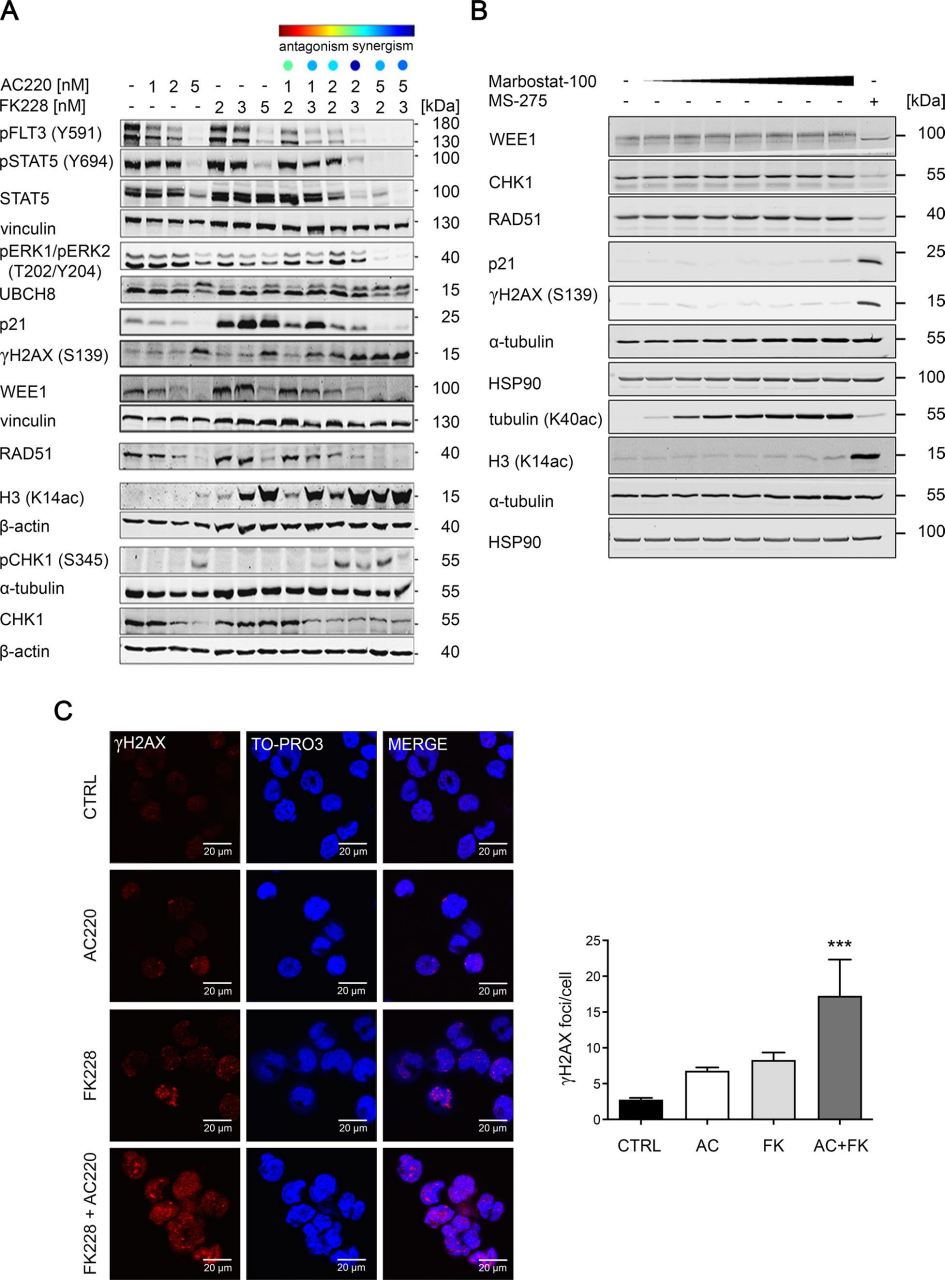
Combining FLT3i and HDACi disrupts FLT3 signaling, attenuates DNA repair protein expression, and increases histone H3 acetylation. **a** MV4-11 cells were incubated for 24 h with either AC220 or FK228 as indicated. Immunoblot analyses were performed to detect FLT3-ITD and its downstream signaling, UBCH8, RAD51, ɣH2AX (S139), histone H3 acetylation, WEE1, phosphorylated CHK1 (S345), and CHK1; Vinculin, α-tubulin and β-actin served as loading control (*n* = 3). **b** MV4-11 cells were incubated with 5, 20, 50, 100, 250, 500, or 1000 nM marbostat-100 or 5 µM entinostat for 24 h. Immunoblot was done as indicated (*n* = 2). **c** MV4-11 cells were left untreated or subjected to with 2 nM AC220 ± 3 nM FK228 for 16 h. Cells were stained for ɣH2AX foci and nuclei were counterstained by TO-PRO3; scale bar represents 20 µm. Foci were quantified using Image J software. Images are representative for 3 independent experiments; ***p < 0.001

Having assessed how AC220 and FK228 affect cell cycle progression and the survival of FLT3-ITD positive cells, we tested how these drugs modulate their targets with the immunoblot method. We first analyzed FLT3-ITD signaling. AC220 and FK228 dose-dependently suppressed the auto-phosphorylation of FLT3-ITD at Y591 and the phosphorylation of its downstream targets STAT5 at Y694 (p-STAT5) and ERK1/ERK2 at T202 and Y204 (p-ERK1/p-ERK2). These effects were potentiated in the combination schemes (Fig. 3A). This finding reflects the synergistic drug interaction that we observed by flow cytometry. The reduction of FLT3-ITD is associated with an accumulation of UBCH8 (Fig. 3A) which accelerates the proteasomal degradation of phosphorylated FLT3 (Buchwald et al. 2013). We further noted a decrease in STAT5 protein expression (Fig. 3A) which is likely due to its caspase-mediated cleavage (Kosan et al. 2013).

The cyclin-dependent kinase inhibitor p21, which controls cell cycle progression, is induced by FK228 in leukemic and solid tumor cells (Sasakawa et al. 2002) and reduced by AC220 in FLT3-ITD positive leukemic cells (Abe et al. 2016). Consistent with these reports, we found that AC220 reduced p21 expression dose-dependently in MV4-11 cells. FK228 increased p21 levels and AC220 dose-dependently rescinded this effect (Fig. 3A). This correlates with a decrease in the FK228-induced G2/M phase arrest when AC220 is co-added (Fig. 2).

An accumulation of ɣH2AX is a marker for replication stress, DNA damage, and apoptosis (Kopp et al. 2019; Rogakou et al. 2000). Our data show an increase of ɣH2AX in MV4-11 cells that were treated with 5 nM AC220 or 5 nM FK228. Synergistic combinations of AC220 and FK228 augmented the accumulation of ɣH2AX by the single agents. We noted this detectably with 1–2 nM AC220 plus 2–3 nM FK228 and 2 nM AC220 plus 3 nM FK228. These drug combinations induced the accumulation of ɣH2AX like 5 nM AC220 and 5 nM FK228 did (Fig. 3A).

These data let us consider a dysregulation of DNA repair protein expression. The tyrosine kinase WEE1 controls S phase and prevents premature mitosis in the presence of damaged DNA (Elbaek et al. 2020; Ghelli Luserna di Rora et al. 2020), the filament protein RAD51 promotes DNA repair through HR (Grundy et al. 2020), and CHK1 is a primary sensor of replication stress and DNA damage (Boudny and Trbusek 2020; Yuan et al. 2014). We noted that single and combined application of AC220 and FK228 reduced WEE1 and RAD51 dose-dependently. The synergistic combination of 2 nM AC220 and 3 nM FK228 attenuated WEE1 and RAD51 as effectively as 5 nM AC220 and 5 nM FK228 did (Fig. 3A). AC220 also reduced CHK1 expression, while FK228 had no significant impact on CHK1. Synergistically toxic combinations of AC220 and FK228 likewise reduced CHK1. This reduction of total CHK1 was associated with its activating phosphorylation at S345 (Fig. 3A). This posttranslational modification is catalyzed by the replication stress/DNA damage activated checkpoint kinase ATR (Boudny and Trbusek 2020; Yuan et al. 2014).

As expected from the literature (Beyer et al. 2017; Li et al. 2019b), FK228 dose-dependently induced the hyperacetylation of histone H3 when given alone and in combination with AC220. Curiously, the accumulation of acetylated histone H3 by FK228 was augmented by AC220 (Fig. 3A).

FK228 preferentially inhibits class I HDACs, but it is also active against HDAC6 (IC_50_ HDAC1/2/3 = 0.00005; IC_50_ HDAC8 = 0.003; IC_50_ HDAC6 = 0.01) (Bradner et al. 2010; Noack et al. 2017). The class IIB deacetylase HDAC6 is frequently dysregulated in leukemic cells (Krämer et al. 2014). To test whether the attenuation of CHK1, WEE1, and RAD51 and the accumulation of p21 and ɣH2AX are due to class I or class IIB HDAC inhibition by FK228, we applied the novel, highly specific HDAC6 inhibitor marbostat-100 (Beyer et al. 2019; Noack et al. 2017; Sellmer et al. 2018) to MV4-11 cells. 5 nM to 1 µM marbostat-100 dose-dependently induced a hyperacetylation of the HDAC6 target tubulin, but marbostat-100 did not modulate CHK1, WEE1, RAD51, p21, and ɣH2AX. In contrast, the HDACi entinostat, which specifically inhibits HDAC1, HDAC2, and HDAC3 (Bradner et al. 2010; Noack et al. 2017) attenuated CHK1, WEE1, and RAD51, and induced an accumulation of acetylated histone H3, p21, and ɣH2AX (Fig. 3B). Hence, HDAC1, HDAC2, and HDAC3 appear as key, druggable regulators of these proteins.

The accumulation of ɣH2AX foci indicates DNA damage foci and pan-staining for ɣH2AX occurs during apoptosis (Kopp et al. 2019; Rogakou et al. 2000). Confocal immunofluorescence microscopy revealed an accumulation of ɣH2AX foci after a 16 h treatment with 2 nM AC220 and 3 nM FK228. This effect became significant in the cotreatment scheme (Fig. 3C). There is no significant induction of apoptosis-associated DNA fragmentation after 16 h (Fig. 2A). This shows that the accumulation of ɣH2AX in response to 2 nM AC220 and 3 nM FK228 is a typical marker for DNA double strand breaks and not a mere readout of apoptosis.

These results show that AC220 and FK228 act on-target. A dysregulation of WEE1, RAD51, and CHK1, together with CHK1 activation, correlates with the accumulation of the DNA damage marker ɣH2AX in leukemic cells that are treated with AC220 and FK228.

### AC220 and RGFP966 synergistically kill FLT3-ITD positive leukemic cells

**Fig. 4:**
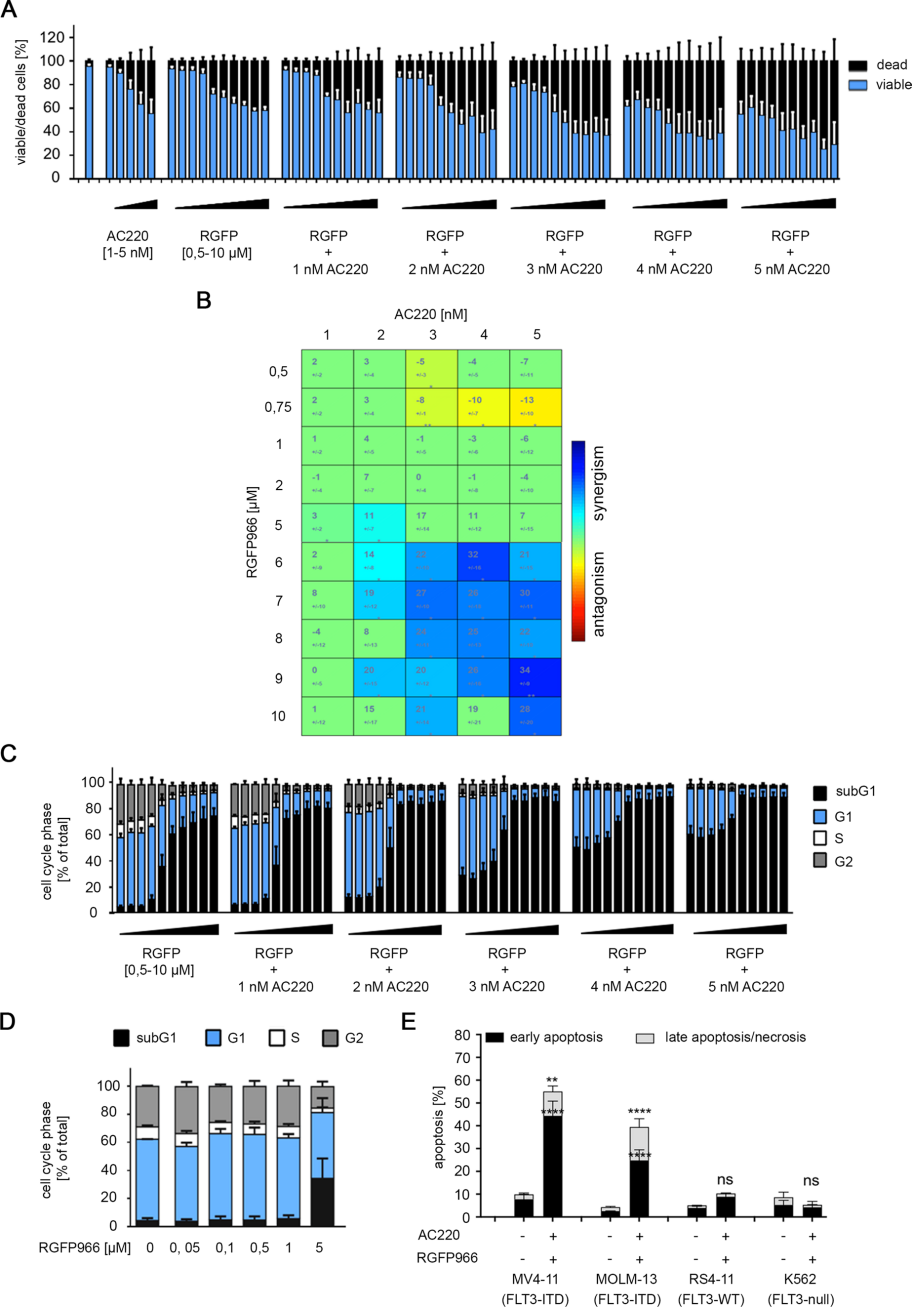
Inhibition of HDAC3 and AC220 are synergistically lethal for FLT3-ITD positive leukemic cells. **a** MV4-11 cells were incubated for 24 h with either 1, 2, 3, 4, or 5 nM AC220 and/or 0.5, 0.75, 1, 2, 5, 7, 8, 9, or 10 µM RGFP966 (increasing doses from left to right shown by the black triangles). Cells were then stained with DAPI and analyzed by flow cytometry for DAPI incorporation. Results represent the mean ± SD (*n* = 5). **b** Determination of synergistic mechanism by combining AC220 and RGFP966 treatment in MV4-11**.** Synergistic effects were determined by using combenefit software. Using the HSA model, we determined the mean synergistic scores. Calculated scores determine drugs interaction as followed (antagonistic effects: *x* ≤ − 10; no interaction effects: − 10 ≤ *x* ≤ 10; synergistic effects: *x* ≥ 10). Results represent the mean ± SD (*n* = 5; HSA). **c** MV4-11 cells were treated with 0.5, 0.75, 1, 2, 5, 6, 7, 8, 9, or 10 µM RGFP966 ± 1, 2, 3, 4, or 5 nM AC220 for 24 h (increasing doses from left to right shown by the black triangles). Cells were stained with PI and analyzed by flow cytometry for different cell cycle phases. Bars show mean ± SD (*n* = 5). **d** MV4-11 cells were treated with 0.05, 0.1, 0.5, 1, or 5 µM RGFP966 for 24 h and were analyzed as mentioned in **a**; ****p < 0.0001 (*n* = 2). **e** MV4-11, MOLM-13, RS4-11, and K562 cells were treated with 2 nM AC220 + 1 µM RGFP966 for 24 h (−, untreated). Cells were stained for annexin-V/PI and analyzed by flow cytometry. Graph represents mean values ± SD of independent experiments; **p < 0.01; ****p < 0.0001 (*n* = 3)

The benzamide HDACi RGFP966 preferentially inhibits HDAC3, and this is sufficient to induce apoptosis of leukemic cells (Beyer et al. 2019). We investigated whether a preferential inhibition of HDAC3 interacts with AC220 against MV4-11 cells. We applied 1–5 nM AC220 and 0.5–10 µM RGFP966 and 50 combinations thereof (66 conditions). Flow cytometry analyses for live/dead cells with DAPI demonstrated that AC220 and RGFP966 dose-dependently killed MV4-11 cells and that this was potentiated when the compounds were applied together (Fig. 4A). 15 of 50 combination killed MV4-11 cells highly synergistically. 3 nM AC220 plus 6 µM RGFP966 were the lowest drug concentrations that interacted synergistically (Fig. 4B).

The measurement of cell cycle distributions and subG1 fractions showed that RGFP966 dose-dependently reduced the numbers of G2/M phase cells and caused the accumulation of cells in subG1 (Fig. 4C). Addition of AC220 dose-dependently increased the subG1 fractions (Fig. 4C). This is coherent with the results that are shown in Fig. 4A.

While FK228 caused an accumulation of MV4-11 cells in G2/M phase (Fig. 2A), RGFP966 stalled these cells in G1 phase (Fig. 4C). We asked whether lower doses of RGFP966 may induce an arrest of MV4-11 cells in the G2/M phase. 50 nM RGFP966 slightly increased an accumulation of MV4-11 cells in G2/M phase, but this was not seen with any of the other concentrations that we applied to these cells (Fig. 4D).

We tested the apoptosis induction by AC220 and RGFP966 with an independent apoptosis assay. We used annexin-V/PI staining to detect early apoptosis (annexin-V positive due to a binding to phosphatidyl-serine at the plasma membrane of apoptotic cells) and late apoptosis (annexin-V and PI positive) (Marx-Blümel et al. 2017). We incubated MV4-11 cells, MOLM-13 cells, RS4-11 cells, and K562 cells (from a 53-year-old woman with chronic myeloid leukemia, FLT3 null) with 2 nM AC220 and 1 µM RGFP966 for 24 h. AC220 and RGFP966 induced apoptosis significantly in MV4-11 and MOLM-13 cells but not in RS4-11 and K562 cells (Fig. 4E).

These data illustrate, that combinations of AC220 plus RGFP966 combine favorably against leukemic cells with FLT3-ITD.

### AC220 and RGFP966 promote their target engagement and attenuate DNA repair proteins

**Fig. 5:**
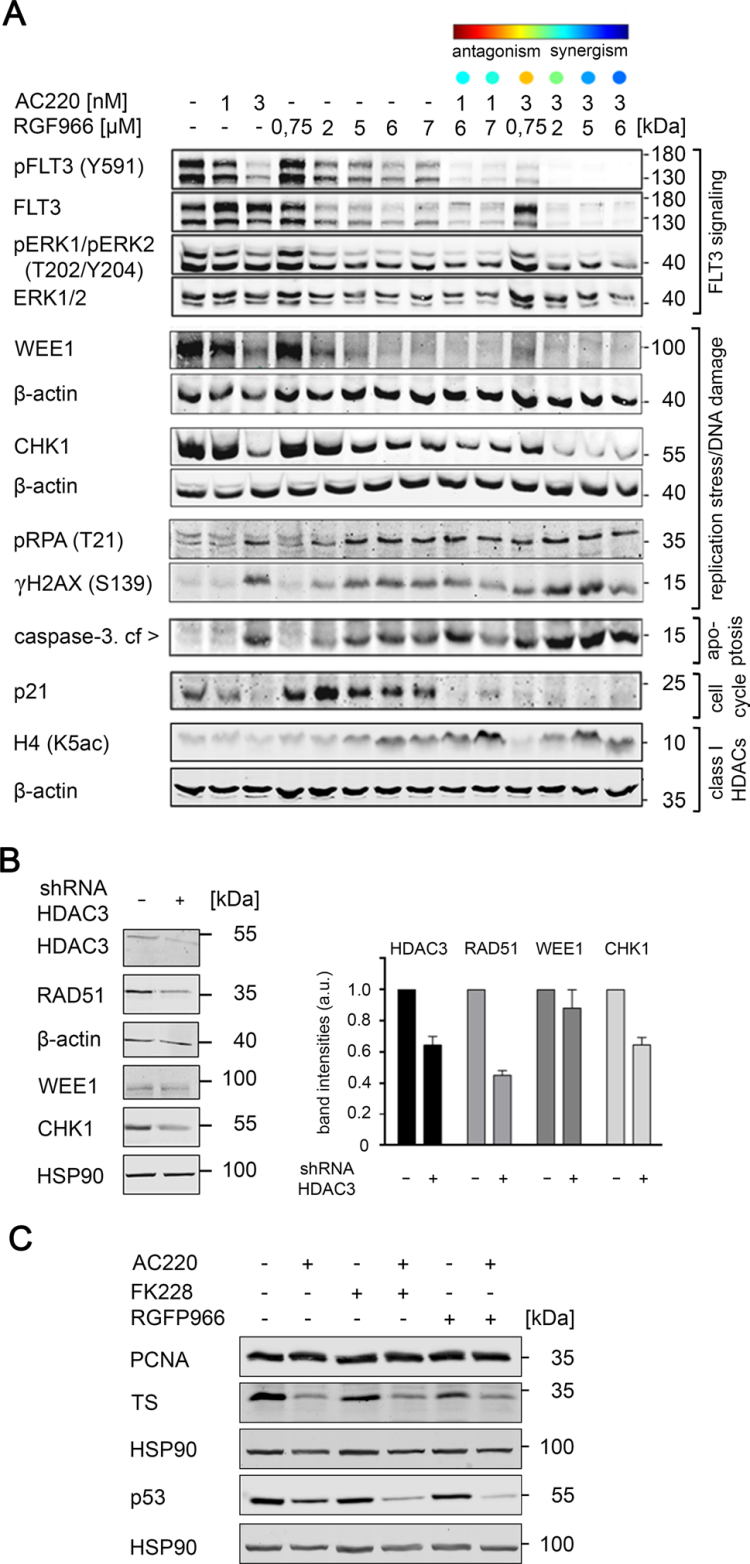
Simultaneous Inhibition of FLT3-ITD and HDAC3 disrupts FLT3 signaling, DNA repair and increases DNA damage. **a** MV4-11 cells were incubated for 24 h with either AC220 and/or RGFP966 in the stated doses. Immunoblot analyses were performed to detect the indicated proteins of FLT3 signaling pathway; β-actin served as loading control for all blots (*n* = 3). **b** MV4-11 cells with an inducible knockdown of HDAC3 by shRNAs were treated with doxycycline for 48 h (−, untreated; + , treated). Lysates of these cells were analyzed as indicated. Band intensities were measured from 2–3 independent immunoblot experiments (a.u., arbitrary units). **c** MV4-11 cells were left untreated (−) or treated with 2 nM AC220 ± 3 nM FK228 or 1 µM RGFP966 for 24 h. Cell lysates were analyzed by immunoblot for PCNA, TS, p53, with HSP90 as loading controls. Results are representatives of two independent experiments

Next, we carried out a biochemical assessment of the interactions of AC220 and RGFP966. We used two concentrations of AC220, five concentrations of RGFP966, and six combinations thereof. AC220 suppressed the phosphorylation of FLT3-ITD at Y591, without reducing total FLT3-ITD. The ratio between the upper, hyperglycosylated and the lower, hypoglycosylated and hyperactive form of FLT3-ITD increased towards the upper band (Fig. 5A). This finding and the suppressed phosphorylation of FLT3-ITD validate the anticipated effects of AC220 (Majothi et al. 2020; Marensi et al. 2021; Scholl et al. 2020). RGFP966 reduced the levels of FLT3-ITD, and this was associated with a reduction of pY591-FLT3-ITD. Combinations of AC220 and RGFP966 potentiated their suppressive effects on FLT3-ITD and the levels of total and phosphorylated FLT3-ITD tied in with the degree of synergistic pro-apoptotic drug interactions. The inhibitory effects of these agents on FLT3-ITD led to a decreased phosphorylation of its targets ERK1/ERK2 (Fig. 5A).

Regarding their effects on replication stress/DNA damage proteins, AC220 and RGFP966 reduced WEE1 and CHK1 dose-dependently and this was potentiated in the cotreatment scheme. The strongest attenuation of WEE1 and CHK1 correlated with the highest levels of synergistic killing of MV4-11 cells (Fig. 5A). The accumulation of single-stranded DNA activates checkpoint kinases which activates the single strand DNA binding protein RPA by phosphorylation on T21 (Anantha et al. 2007). Both RPA phosphorylated on T21 and ɣH2AX were increased dose-dependently by RGFP966 and AC220. All combinations thereof led to increased signals of phosphorylated RPA and H2AX as well as cleaved caspase-3 (Fig. 5A). This confirms that these agents induce DNA damage and apoptosis.

Like FK228, RGFP966 dose-dependently increased p21 and 1 nM AC220 was sufficient to block the RGFP966 induced accumulation of p21 (Fig. 5A). As seen with FK228 and AC220 for histone H3 acetylation (Fig. 3A), the dose-dependent accumulation of acetylated histone H4 by RGFP966 was augmented by AC220 (Fig. 5A).

To corroborate these data with a genetic model, we transduced MV4-11 cells with an inducible shRNA against HDAC3. The knockdown of HDAC3 attenuated RAD51, WEE1, and CHK1 (Fig. 5B).

Since RAD51 and CHK1 are regulated by mutant FLT3 (Bagrintseva et al. 2005; Kurosu et al. 2013) and by the cell cycle (Nikolova et al. 2017), we additionally probed immunoblots for the general DNA synthesis protein proliferating cell nuclear antigen (PCNA) and for thymidylate synthase (TS). Like RAD51, CHK1, and WEE1 (Figs. 3A, 5A), TS was also decreased by AC220, but this was not potentiated by FK228 and RGFP966. PCNA was not affected by any of these drugs (Fig. 5C).

Given that the transcription factor p53 promotes the expression of p21 in leukemic cells (Yan et al. 2020), we speculated that the reduction of p21 expression by AC220 (Figs. 3A, 5A) is associated with a decrease of p53. Indeed, AC220 decreased p53. FK228 and RGFP966 did not alter p53 levels but augmented the suppressive effect of AC220 on p53 (Fig. 5C).

These data illustrate that AC220 and RGFP966 act on-target and that they dysregulate cell cycle progression and proteins that control cellular responses to replication stress and DNA damage. AC220 and RGFP966 potentiate their specifically effects on signaling pathways and HDAC3 is necessary for the expression of DNA repair proteins in leukemic cells.

## Discussion

AML with FLT3-ITD mutations shows poor responses to current therapeutic approaches. Improved TKi as well as rationally defined combinatorial treatments can be milestones for the management of this disease. This could render FLT3-ITD positive AML into a curable disease. This has been achieved for most cases of acute promyelocytic leukemia which is driven by the leukemia fusion protein PML-RARα (Perl 2019). In FLT3-ITD positive leukemic cells that are treated with AC220 or marbotinib plus FK228 or RGFP966, we observe an increase of the cell populations that accumulate DAPI and PI, that have phosphatidylserine on their cell surface, and that display cleaved caspase-3. These are typical markers of cell death, with the activation of caspase-3 being a point of no return for the demise of cells by apoptosis (Boice and Bouchier-Hayes 2020). Thus, our data demonstrate that such drug combinations eliminate cells with mutated FLT3 through apoptosis. Leukemic cells with wild-type FLT3 are thought unaffected by these drugs, suggesting selectivity for cells with mutant FLT3. Analysis of a larger cell line panel is necessary to verify this hypothesis.

Inhibitors of FLT3-ITD and HDACi are known to cause cell cycle arrest (Li et al. 2020; Luo and Li 2020; Majothi et al. 2020; Marensi et al. 2021; Scholl et al. 2020). FK228 induced p21 and cell cycle arrest in G2/M phase. AC220 suppresses p21 expression and arrests cells in G1 phase. Moreover, AC220 antagonizes the induction of p21 in FK228-treated MV4-11 and attenuates the FK228-induced G2/M phase arrest. These data suggest that p21 is required to stall FK228-treated MV4-11 cells in G2/M phase. In contrast to FK228, RGFP966 induces p21 and an accumulation of cells in G1 phase. These differences may be due to the differences in the inhibitor profiles. While the benzamide RGFP966 preferentially inhibits HDAC3 over HDAC1/HDAC2, FK228 additionally blocks HDAC8 (Beyer et al. 2019; Matthews et al. 2015; Pojani and Barlocco 2020). Inhibition of HDAC8 increases the acetylation of the structural maintenance of chromosomes protein-3 (Deardorff et al. 2012) and this may contribute to the G2/M arrest in FK228-treated MV4-11 cells. This idea is supported by the finding that a specific inhibition of HDAC8 with the compound PCI-34051 can delay cell cycle progression of breast cancer cells (Dasgupta et al. 2016).

AC220 suppresses p21 expression and prevents the accumulation of p21 in response to HDACi. This mechanism could be pro-apoptotic because p21 suppresses caspase-3 activation in HDACi-treated tumor cells (Krämer et al. 2008; Rosato et al. 2003, 2004). Consistent herewith, we show that a reduction of p21 is associated with increased apoptosis induction in leukemic cells. This does not imply that an induction of p21 by FK228 and RGFP966 prevents apoptosis induction per se. Concomitant induction of p21 and of apoptosis by HDACi occurs in various other cancer cells; for example, in acute lymphoid leukemia and chronic myeloid leukemia cells that are treated with the depsipeptide spiruchostatin B (Kanno et al. 2012) and in solid tumor cells (Wang et al. 2012) and histiocytoma cells (Sasakawa et al. 2002) that are treated with FK228. The increase of p21 in MV4-11 cells is not linked to an increase in p53 levels. This can be explained by sufficient levels of basal p53, and it needs to be considered that class I HDACs critically control p53 by direct lysine acetylation (Marx et al. 2021).

The data above provide two not mutually exclusive explanations for the inhibitory effect of AC220 on p21 expression. First, we observed a loss of p53 which was recently shown to promote the expression of p21 in FK228-treated MV4-11 cells (Yan et al. 2020). Second, we noted that AC220 activates CHK1 which has a suppressive effect on the translation of the *p21* mRNA (Beckerman et al. 2009).

We further noted a pronounced target engagement of the AC220 and the HDACi. FK228 and RGFP966 potently reduce FLT3-ITD and AC220 augments this effect. These data are consistent with the reported degradation of FLT3-ITD upon a treatment with entinostat, which specifically inactivates HDAC1, HDAC2, and HDAC3 (Nishioka et al. 2008). Moreover, we here confirm our data for FLT3-ITD positive AML cells that are treated with combinations of panobinostat plus AC220 (Pietschmann et al. 2012) and extend them by showing a survival dependency of FLT3i-treated leukemic cells on HDAC3.

Secondary mutations of FLT3-ITD in its TKD are selected during the therapy with AC220. These mutated cells are more resistant than FLT3-ITD against AC220 and cause relapse and poor patient prognosis (Antar et al. 2020; Perl 2019). We recently presented marbotinib as specific inhibitor of FLT3-ITD and FLT3-TKD oncogenes (Sellmer et al. 2020). We show here that AC220 combines favorably with the FDA-approved HDACi FK228 against leukemic cells carrying FLT3-ITD. Hence, the administration of marbotinib and class I HDACi may be a valid strategy against leukemic cells with FLT3-TKD mutants.

Surprisingly, AC220 increased the HDACi-induced accumulation of acetylated histones H3/H4. We can only speculate whether this is a consequence of cell death, a modulation of HDACs and histone acetyltransferases, or alterations in the cellular pool of free acetyl-CoA due to altered mitochondrial metabolism.

This work further reveals that AC220 plus HDACi reduce the levels of CHK1, WEE1, RAD51, and TS. These findings support previous data on a loss of CHK1, WEE1, and RAD51 in leukemic cells in vitro and in vivo when they are treated with HDACi (vorinostat, panobinostat, FK228, fimepinostat) (Dai et al. 2013; Li et al. 2019a; Qi et al. 2015; Xie et al. 2013; Zhao et al. 2017; Zhou et al. 2015). As FLT3-ITD drives the cell cycle as oncogene in MV4-11 cells, its inhibition consequently stalls cells in G1 phase and this can decrease DNA repair proteins (Bagrintseva et al. 2005; Kurosu et al. 2013). However, since AC220 and FK228 cause different types of cell cycle arrest, their suppressive effects on CHK1, WEE1, RAD51, and TS are unlikely due to altered cell cycle progression. Moreover, the combined applications of the drugs decrease therese proteins more strongly than the single drugs and this is not associated with a more pronounced arrest in one cell cycle phase.

This genetic depletion of WEE1, CHK1, and RAD51 as well as pharmacological drugs that target CHK1 (MK-1775, MK-8776, AZD1775) sensitize leukemic cells to apoptosis induction by HDACi. This is linked to a loss of cell cycle control and genomic intactness (Dai et al. 2013; Li et al. 2019a; Qi et al. 2015; Xie et al. 2013; Zhao et al. 2017; Zhou et al. 2015). It should be considered that the reduction of CHK1 does not necessarily stand for its inactivation as we see an activation of CHK1 (verified by its phosphorylation at S345). This was similarly observed in FLT3-ITD positive and negative leukemic cells that were treated with vorinostat (Zhou et al. 2015). Since the genetic depletion of CHK1 sensitizes cells to HDACi-induced apoptosis (Dai et al. 2013; Li et al. 2019a; Qi et al. 2015; Xie et al. 2013; Zhao et al. 2017; Zhou et al. 2015), we speculate that the activation of CHK1 by HDACi is a cellular survival response to the HDACi-induced replication stress and DNA damage. It also appears that HDACs can have a differential impact on DNA repair proteins in various leukemic cell models. In THP1 leukemic cells (AML cells from a one-year-old male child), FK228 reduced CHK1 and RAD51. This was also seen upon a genetic depletion of HDAC1/HDAC2 but not with RGFP966 (Zhao et al. 2017). Irrespective of such cell type-specific details and the need to fully characterize how class I HDACs share the workload to maintain DNA repair protein expression, the attenuation of WEE1, CHK1, RAD51, and TS are general and functionally important effects that these epigenetic drugs induce. The reduction of these proteins may serve as a pharmacodynamic marker to assess the efficacy of HDACi treatment in patients. Such markers are relevant because the general accumulation of acetylated histones does not necessarily inform about whether the HDACi will give beneficial effects in vivo (Müller and Krämer 2010; Wang et al. 2018).

Also coherent with previous reports (Dai et al. 2013; Li et al. 2019a; Qi et al. 2015; Xie et al. 2013; Zhao et al. 2017; Zhou et al. 2015), we found that the HDACi-induced loss of CHK1, WEE1, p21, and RAD51 was associated with cell cycle dysregulation and an accumulation of DNA strand breaks in AML cells with FLT3-ITD. This is linked to apoptosis which is a typical consequence of the loss of cell cycle control, DNA integrity, and tumorigenic signaling (Lee et al. 2017; Yue et al. 2020).

Taken together, we reveal that inhibitors of class I HDACs and of mutant FLT3 combine favorably against AML cells. This beneficial effect is associated with DNA damage and a loss of FLT3-ITD signaling.

## Supplementary Information

Below is the link to the electronic supplementary material.

Supplementary file1 Supplementary Fig. S1. RS4-11 cells were treated with 5 mM hydroxyurea (HU) for 24 h. Cells were stained with PI and analyzed for cell cycle distribution by flow cytometry. Bars show mean ± SD; **p < 0.01; ****p < 0.0001. Graph represents data from different independent experiments (n=2). (PNG 607 KB)

Supplementary file2 (PPTX 18960 KB)

## Data Availability

Uncropped images of immunoblot analyses are provided as Supplementary Information. Further data supporting the findings of this study are available from the corresponding author on scientific request.
