# Supplementary figures and images for: Inhibitors of class I HDACs and of FLT3 combine synergistically against leukemia cells with mutant FLT3
Source: Arch Toxicol. 2021 Oct 19;96(1):177–93. doi: 10.1007/s00204-021-03174-1 (PMC8748367; doi:10.1007/s00204-021-03174-1)

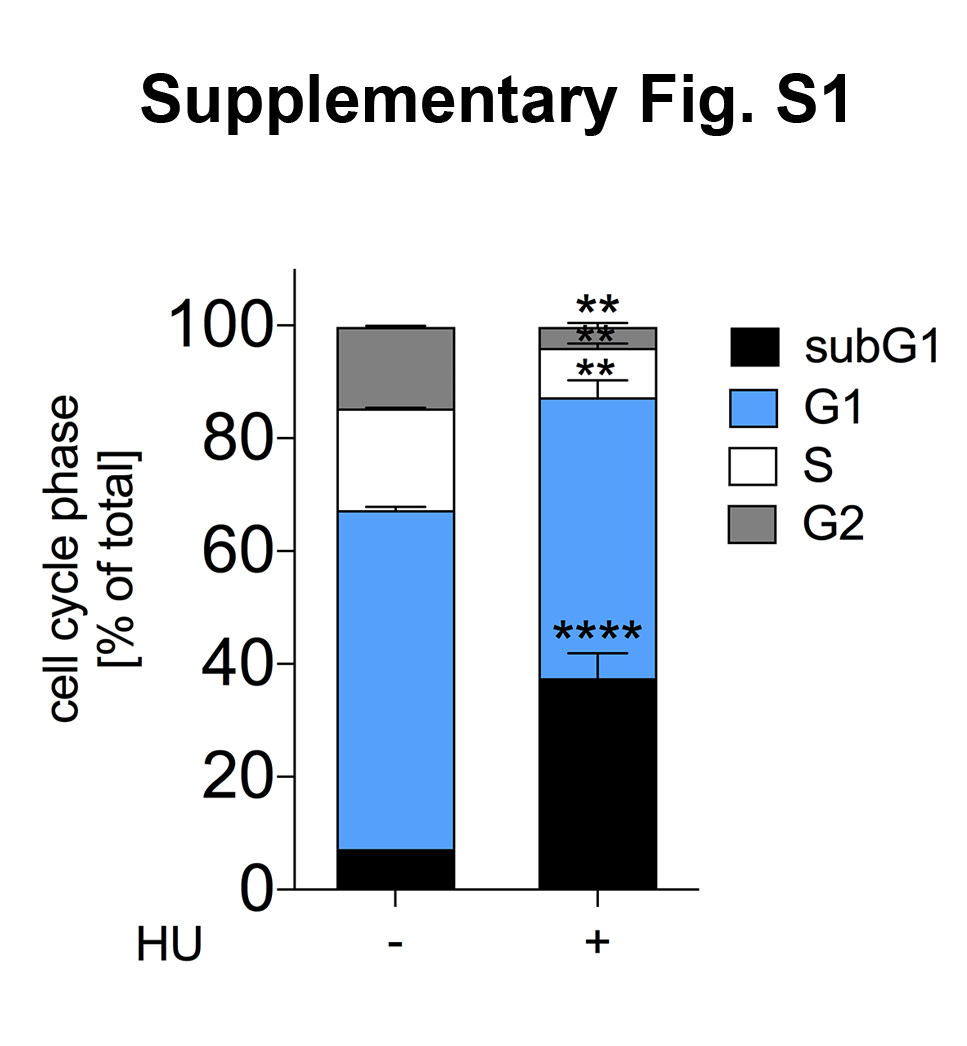

Supplement: Supplementary file 1 — Supplementary file1 Supplementary Fig. S1. RS4-11 cells were treated with 5 mM hydroxyurea (HU) for 24 h. Cells were stained with PI and analyzed for cell cycle distribution by flow cytometry. Bars show mean ± SD; **p < 0.01; ****p < 0.0001. Graph represents data from different independent experiments (n=2). (PNG 607 KB) [file 204_2021_3174_MOESM1_ESM.png]
