# Supplementary material for: Inhibitors of class I HDACs and of FLT3 combine synergistically against leukemia cells with mutant FLT3
Source: Arch Toxicol. 2021 Oct 19;96(1):177–93. doi: 10.1007/s00204-021-03174-1 (PMC8748367; doi:10.1007/s00204-021-03174-1)
Supplement: Supplementary file 2 — Supplementary file2 (PPTX 18960 KB) [file 204_2021_3174_MOESM2_ESM.pptx]

## Slide 1
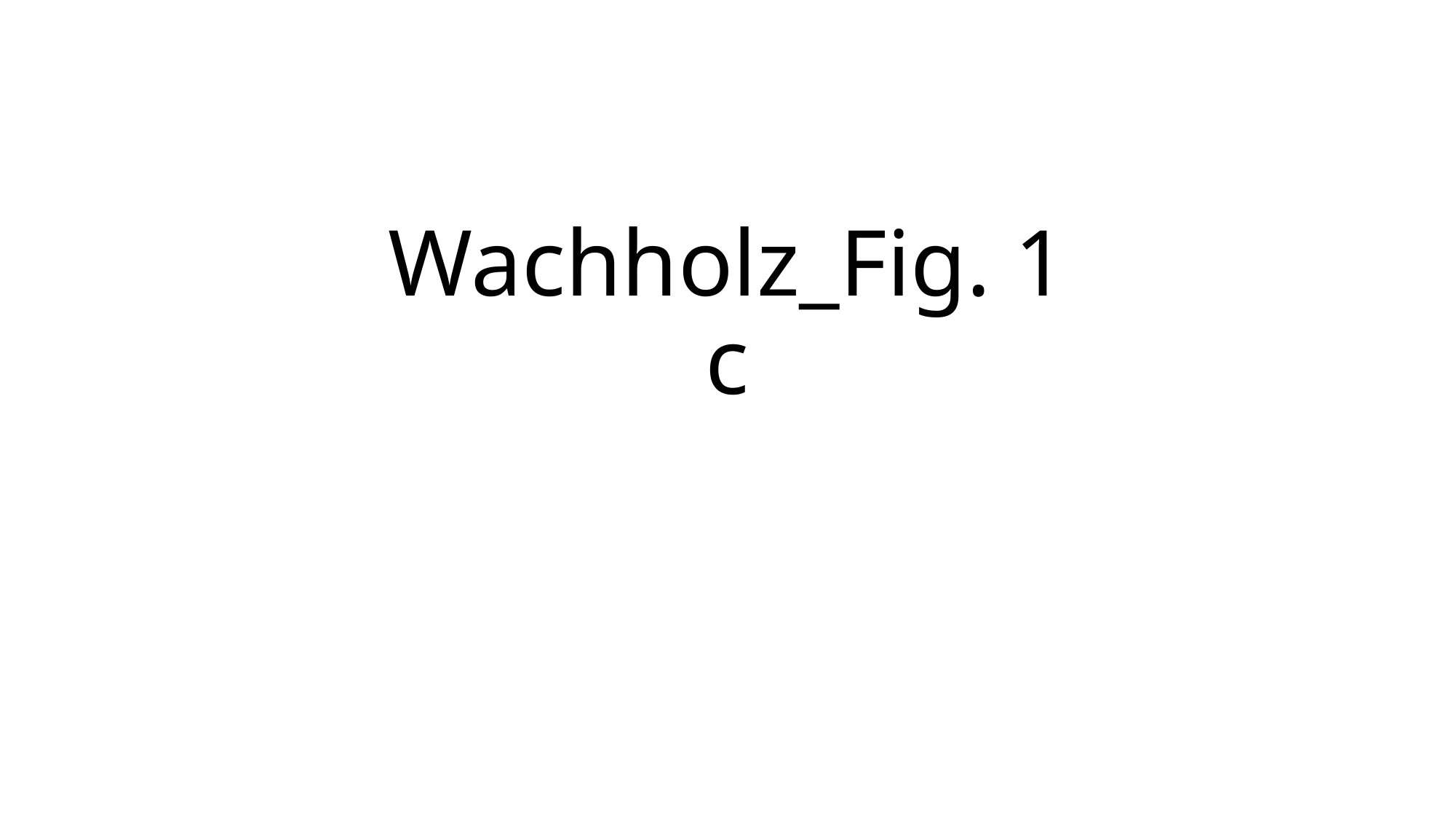

# Wachholz_Fig. 1 c

## Slide 2
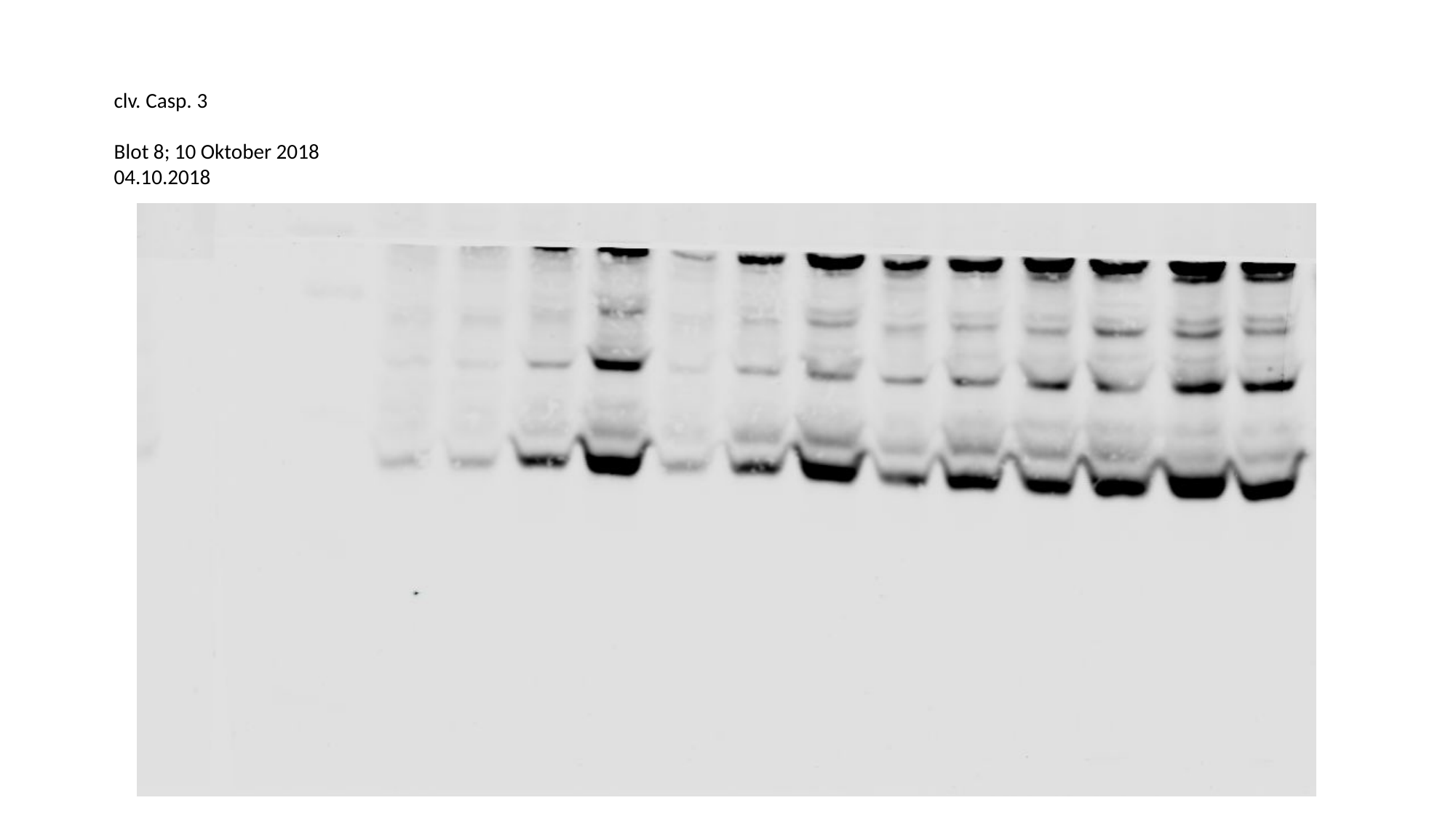

clv. Casp. 3
Blot 8; 10 Oktober 2018
04.10.2018

## Slide 3
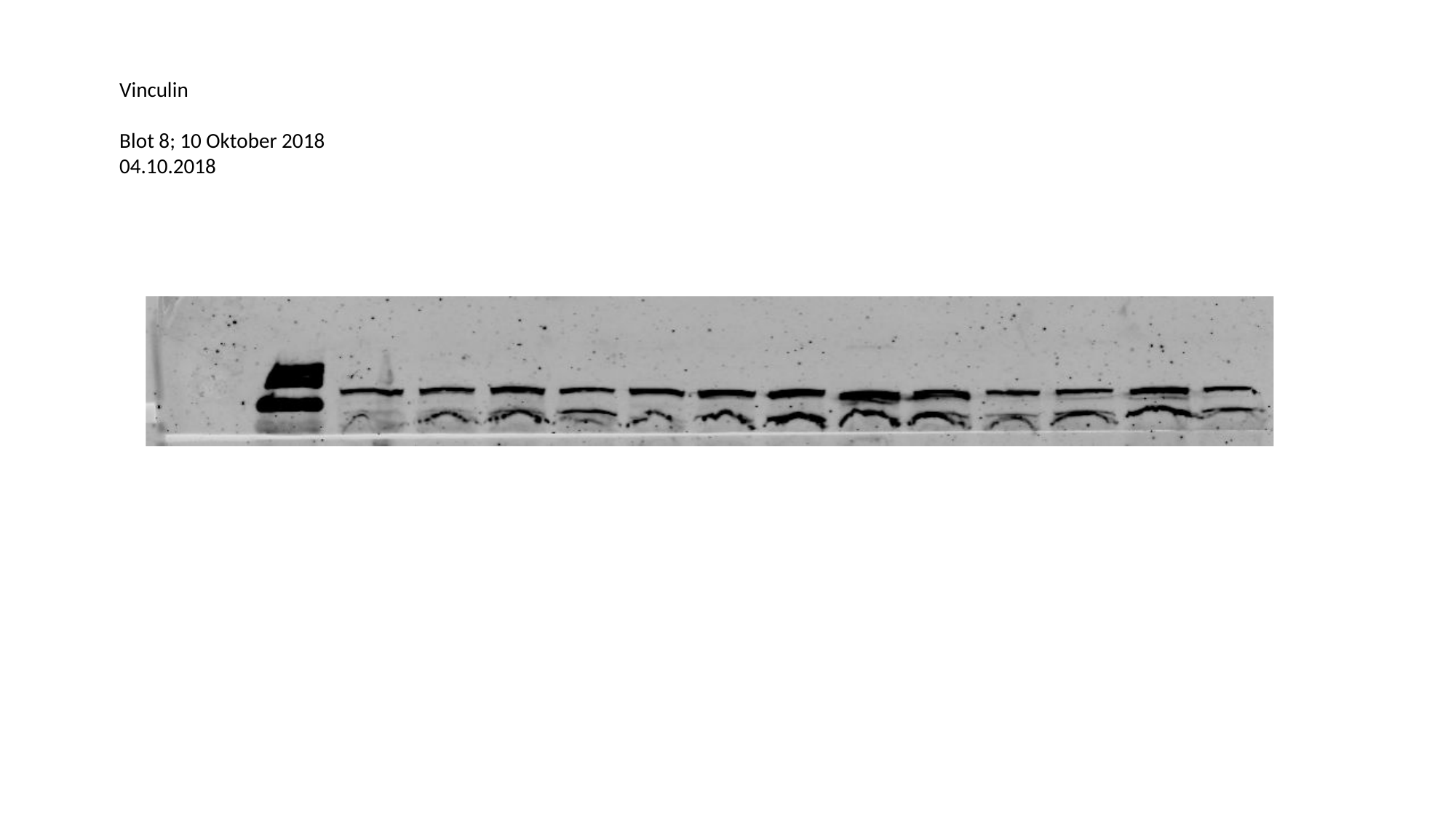

Vinculin
Blot 8; 10 Oktober 2018
04.10.2018

## Slide 4
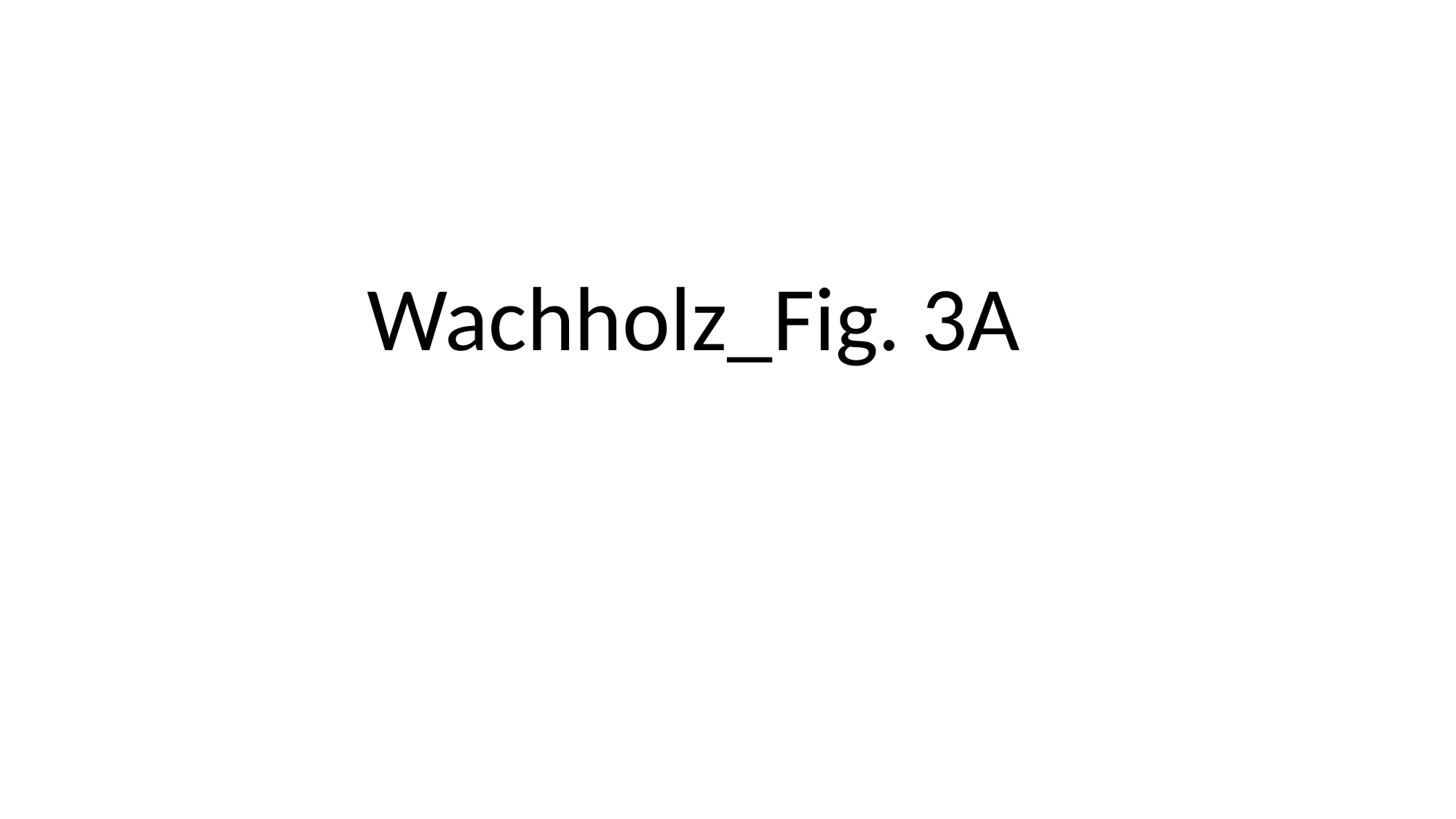

Wachholz_Fig. 3A

## Slide 5
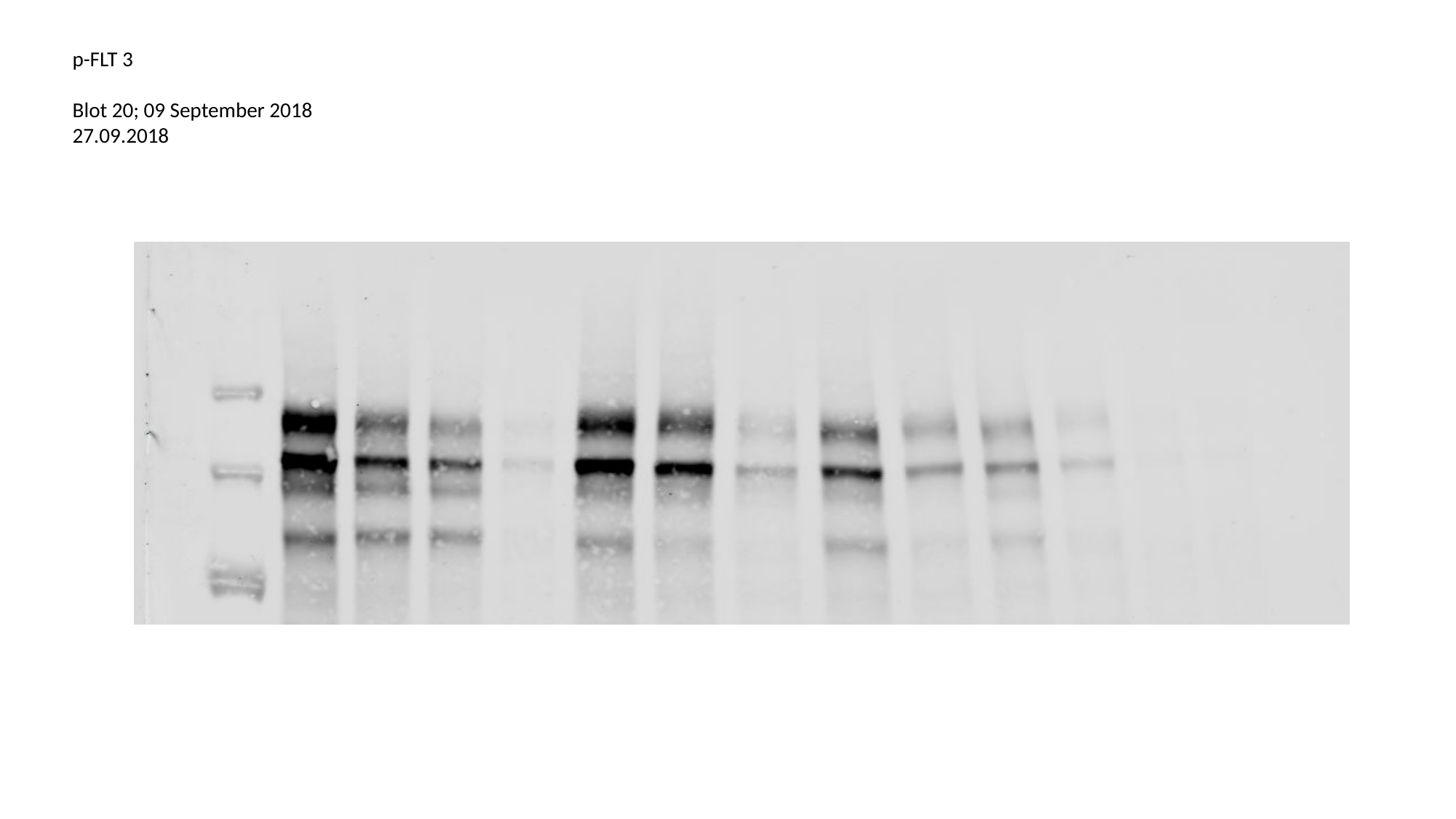

p-FLT 3
Blot 20; 09 September 2018
27.09.2018

## Slide 6
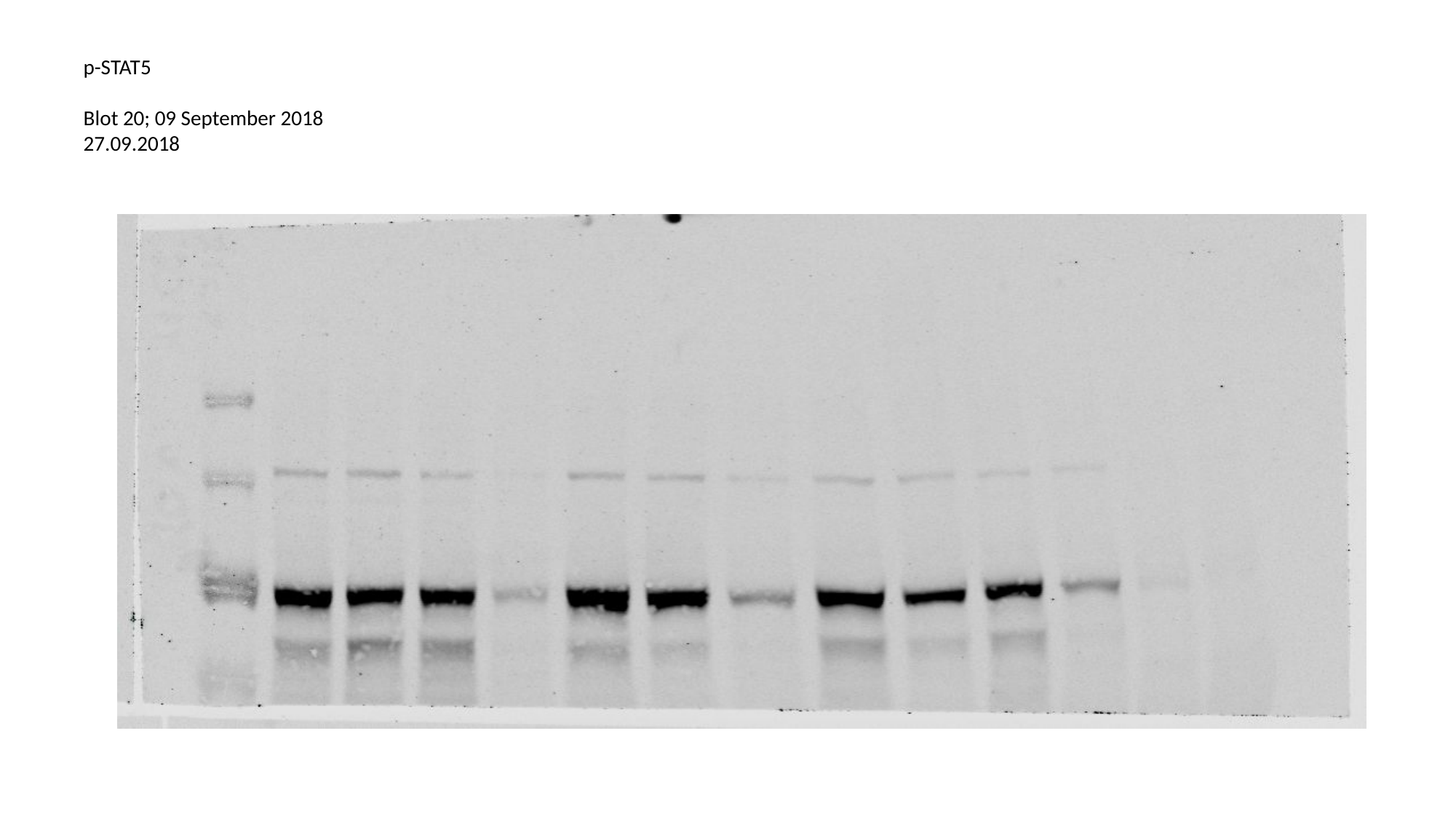

p-STAT5
Blot 20; 09 September 2018
27.09.2018

## Slide 7
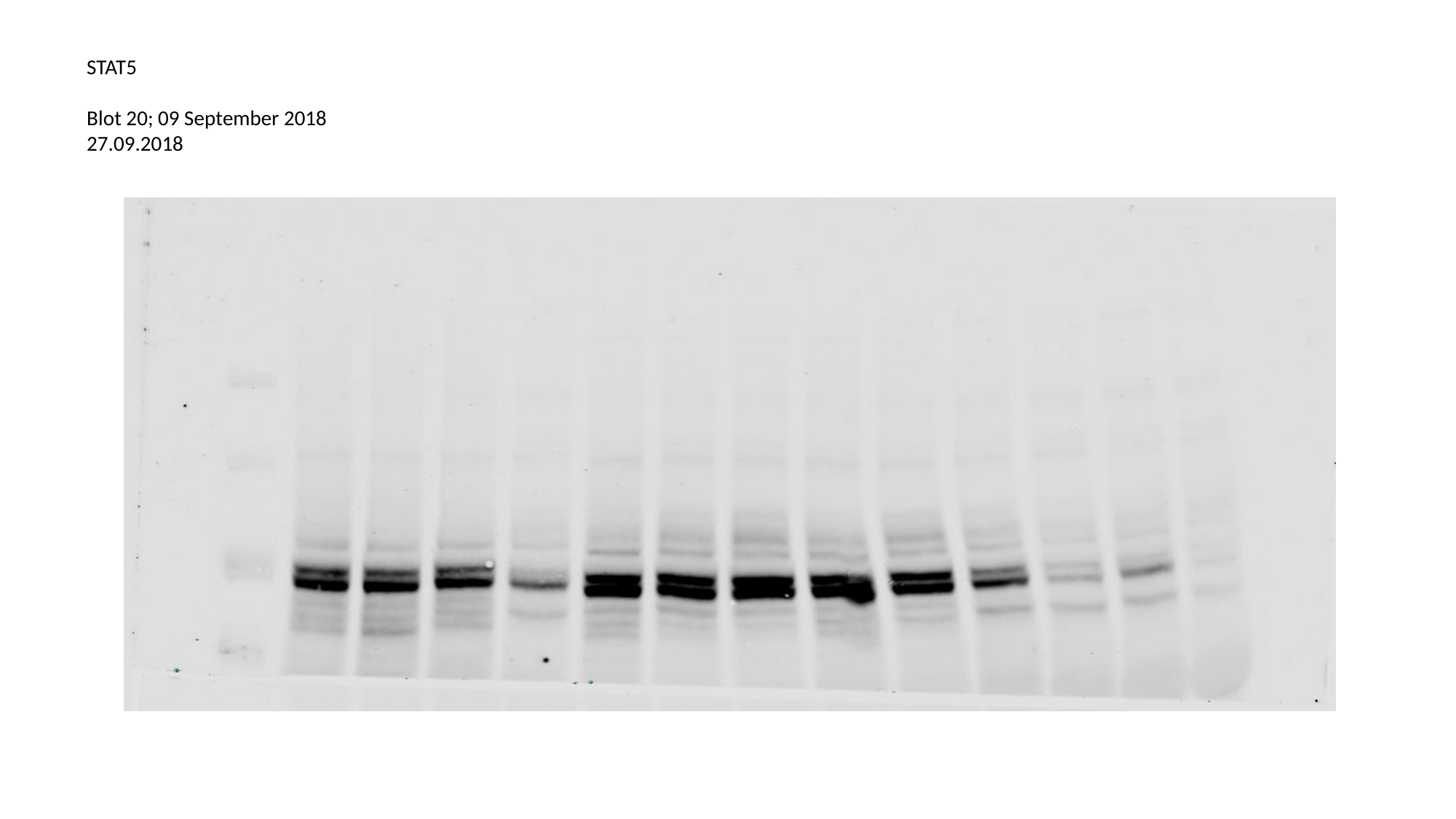

STAT5
Blot 20; 09 September 2018
27.09.2018

## Slide 8
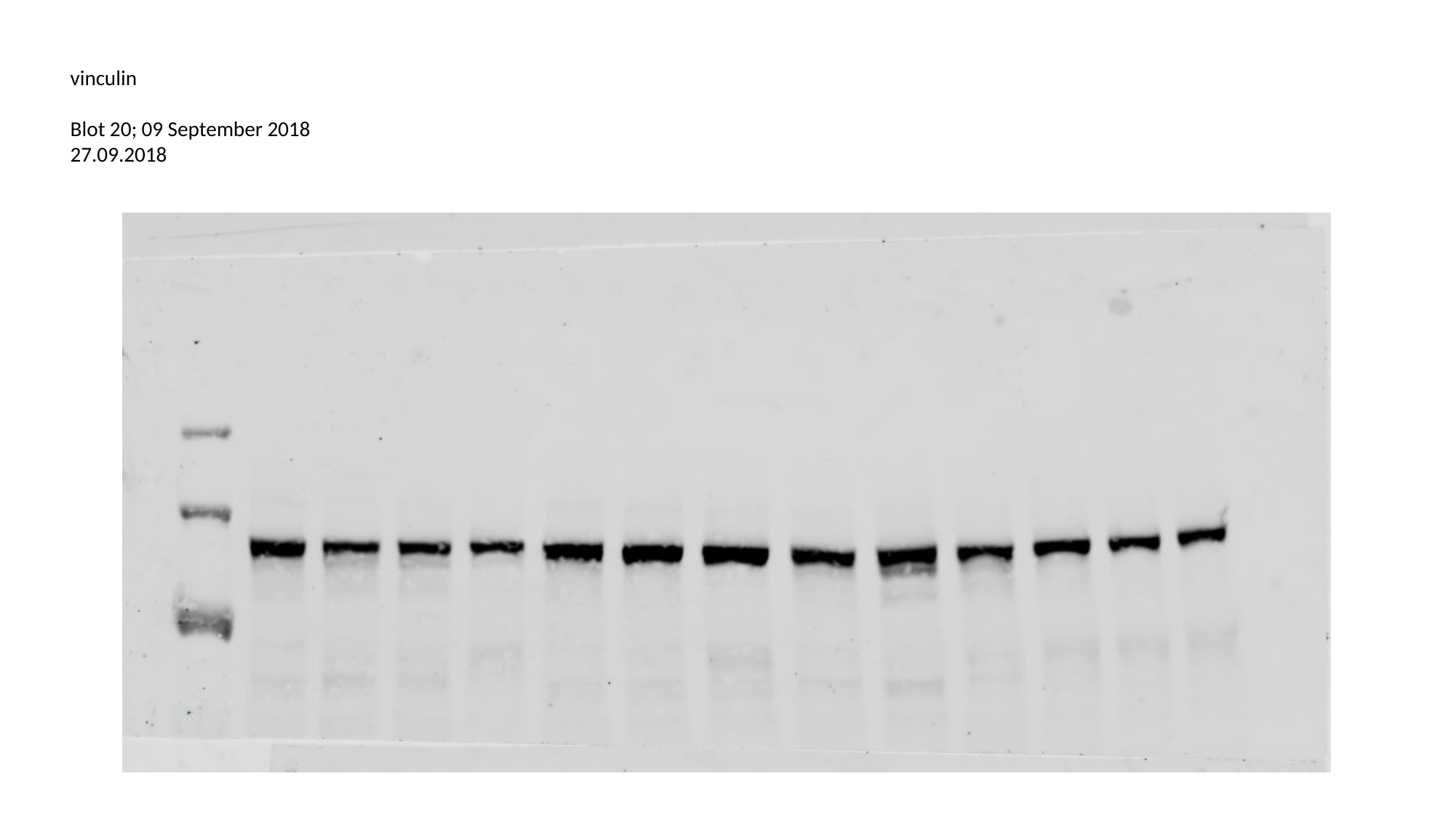

vinculin
Blot 20; 09 September 2018
27.09.2018

## Slide 9
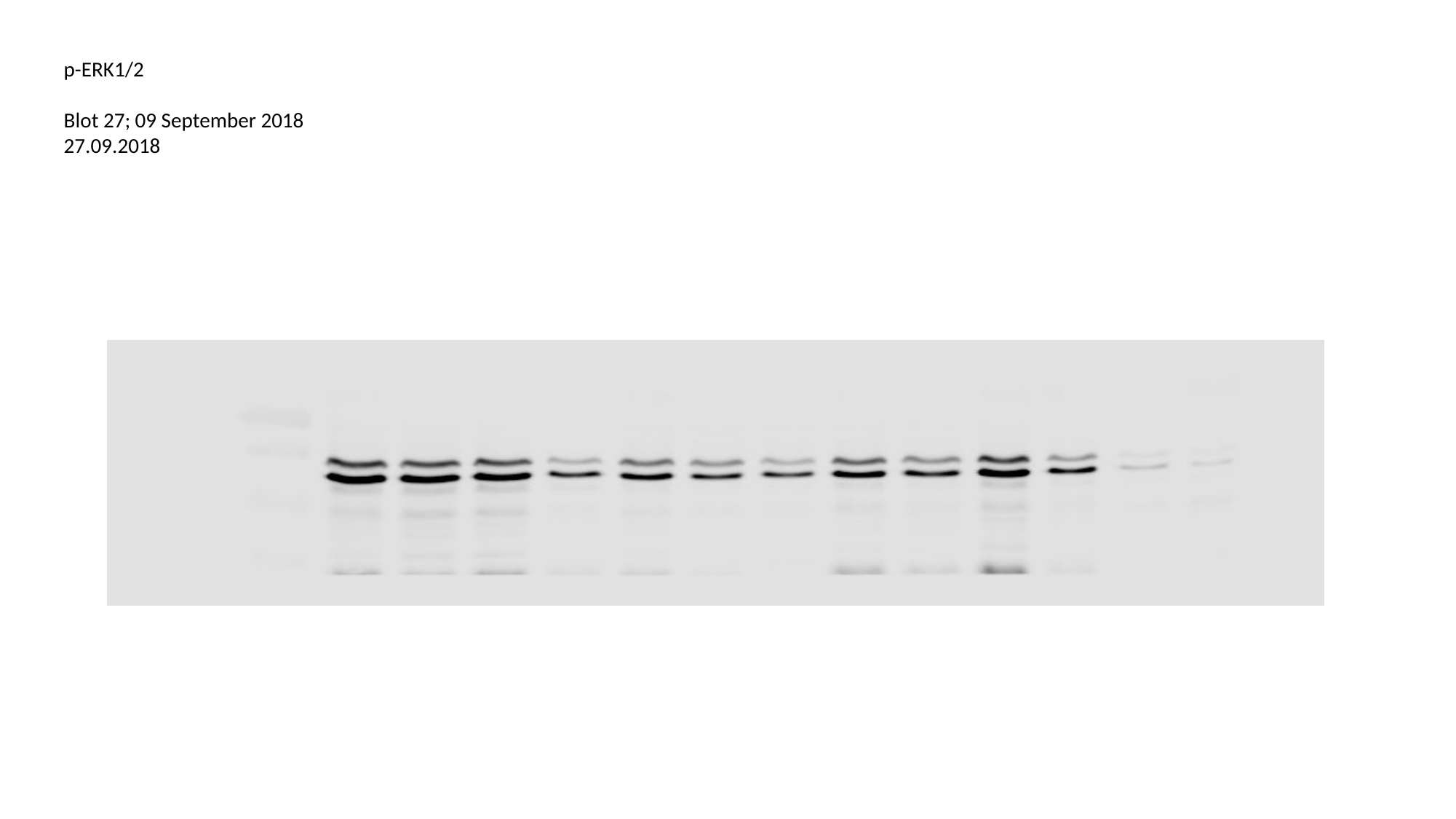

p-ERK1/2
Blot 27; 09 September 2018
27.09.2018

## Slide 10
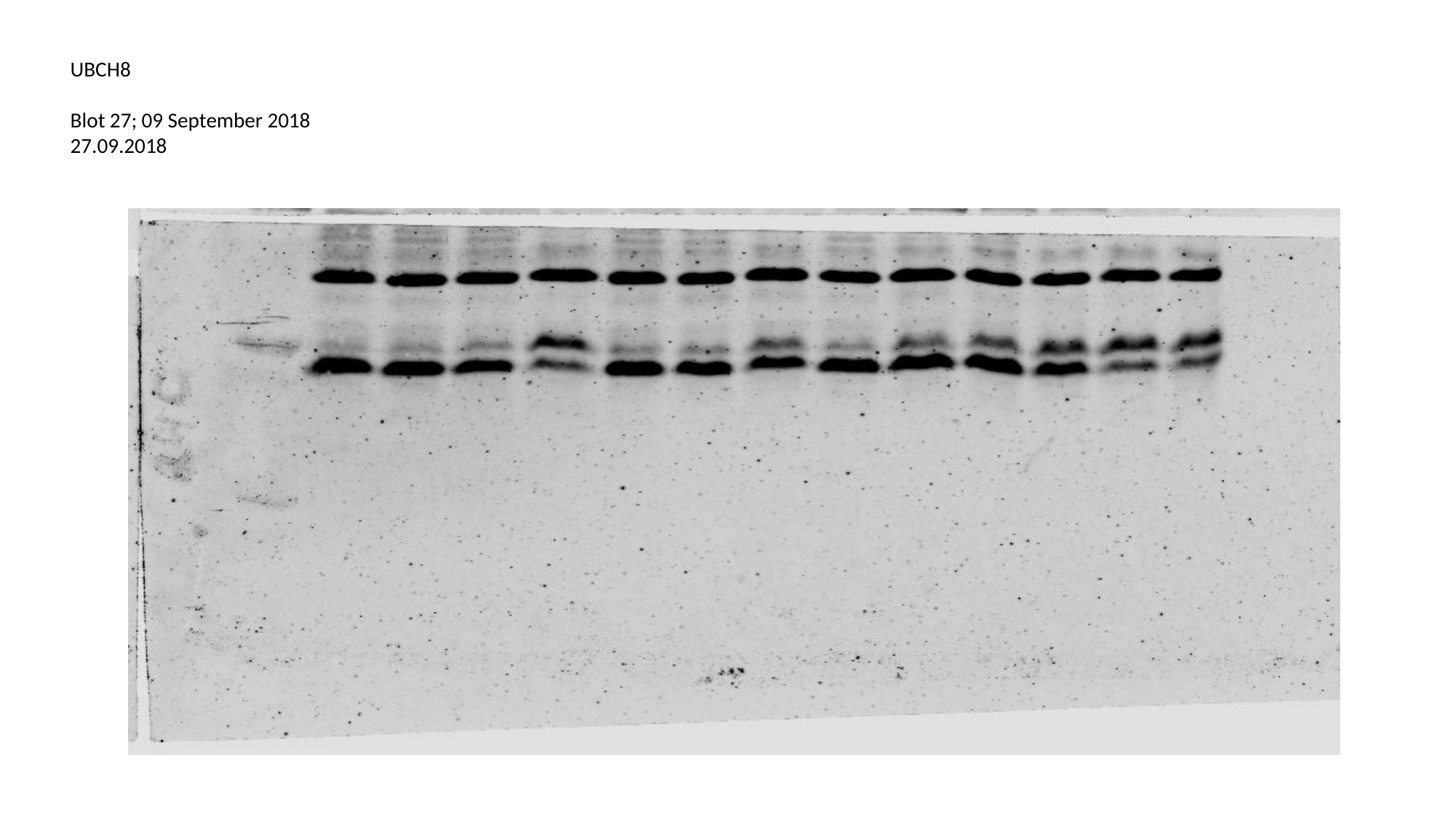

UBCH8
Blot 27; 09 September 2018
27.09.2018

## Slide 11
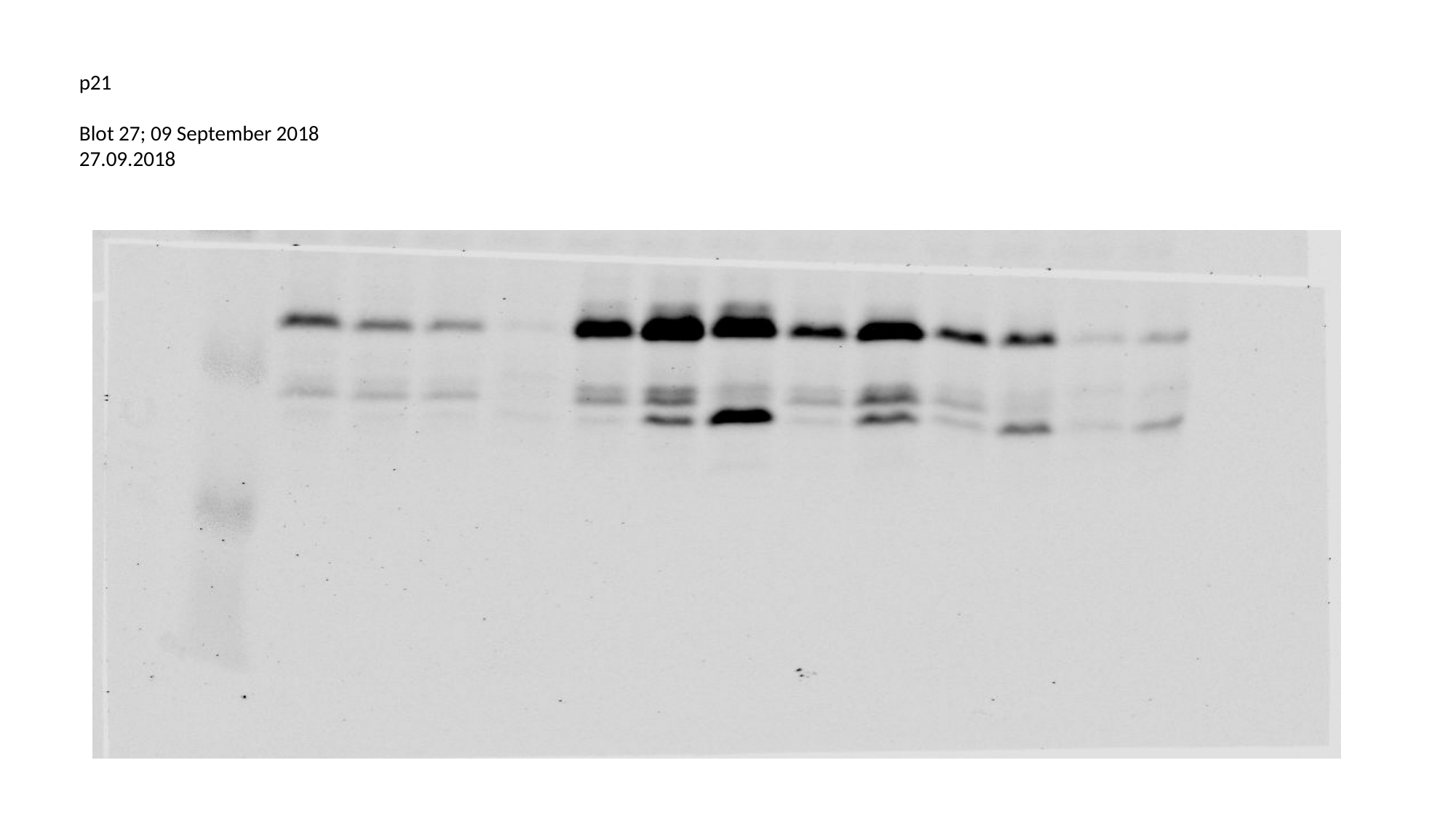

p21
Blot 27; 09 September 2018
27.09.2018

## Slide 12
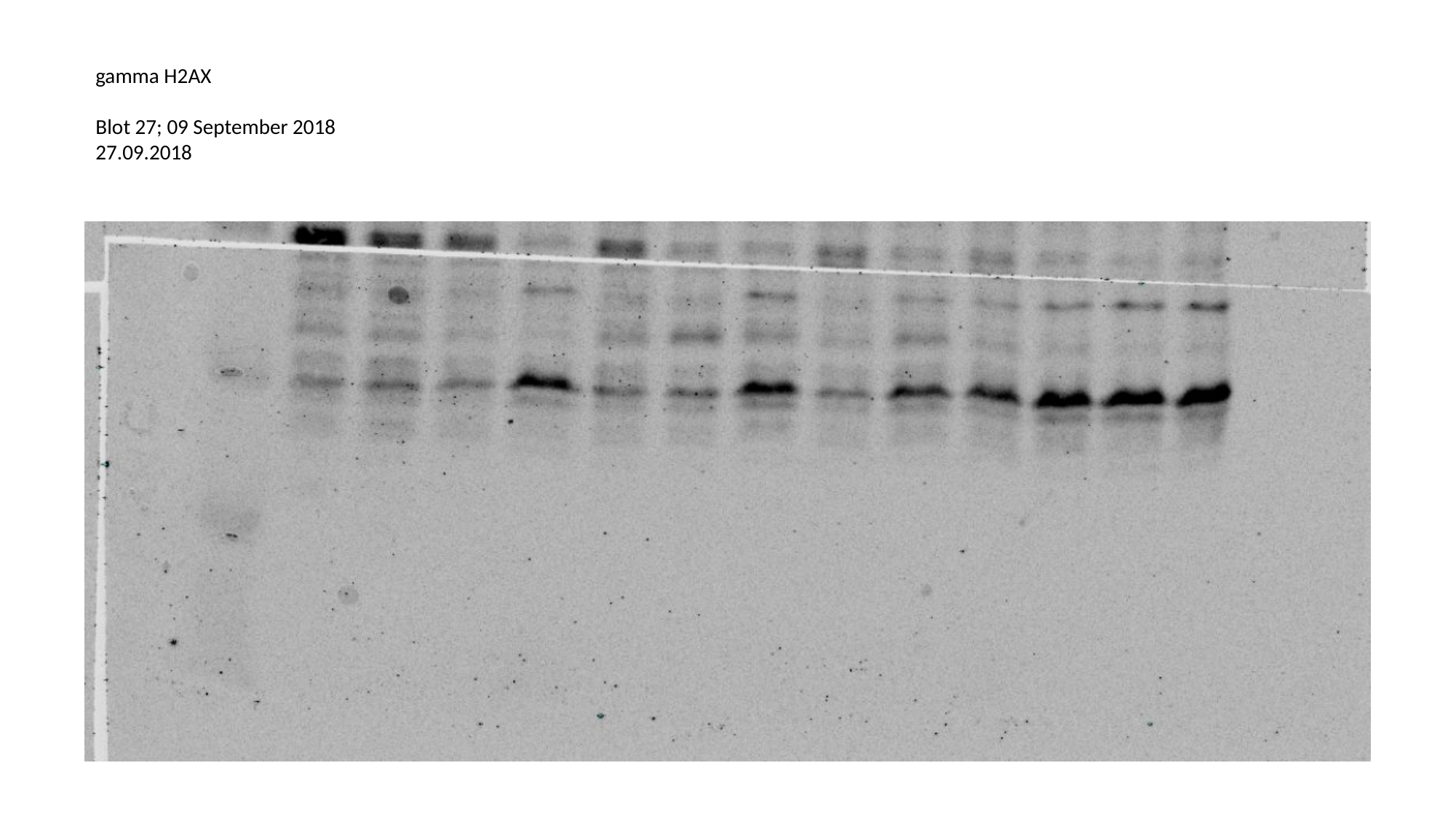

gamma H2AX
Blot 27; 09 September 2018
27.09.2018

## Slide 13
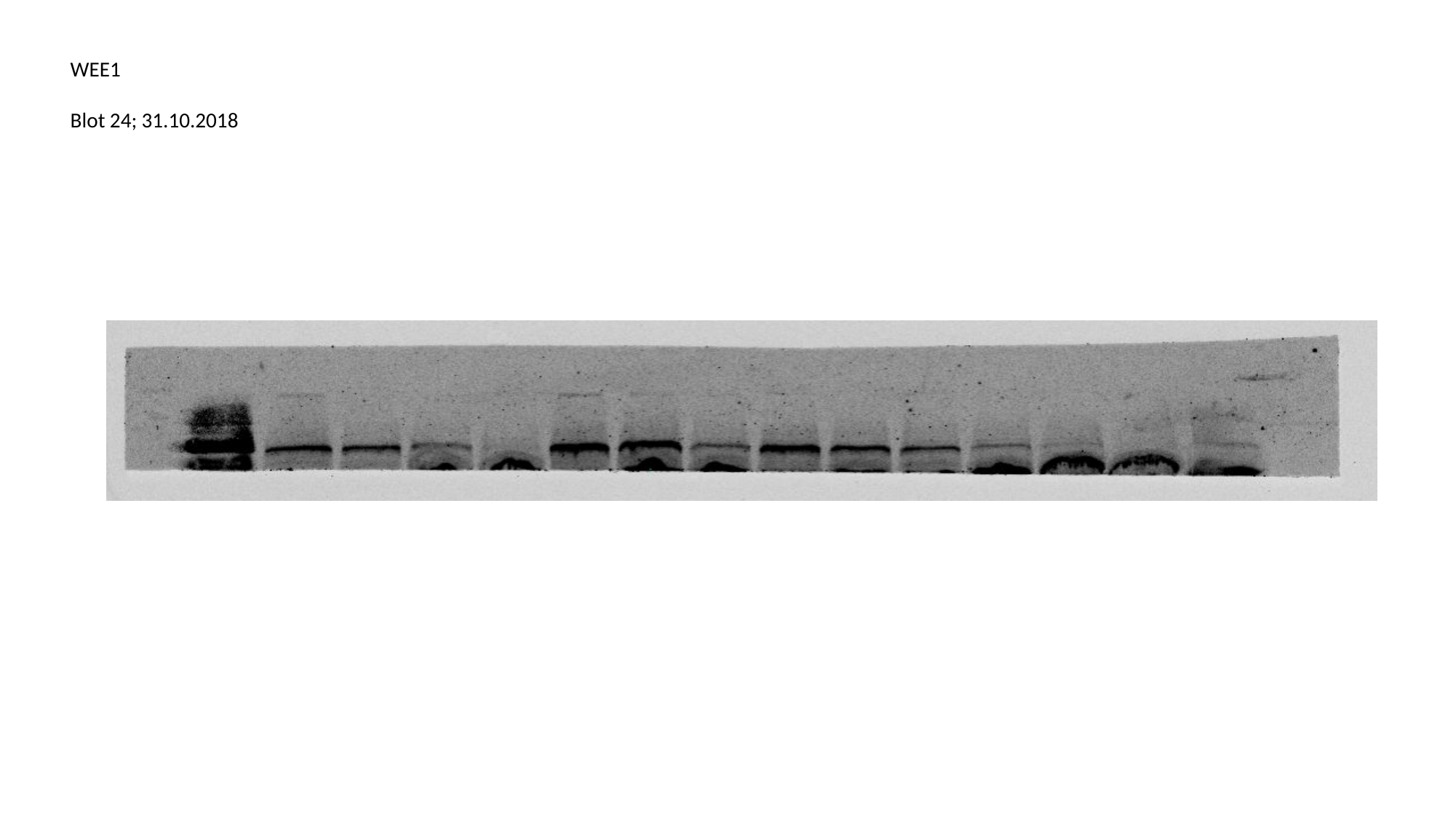

WEE1
Blot 24; 31.10.2018

## Slide 14
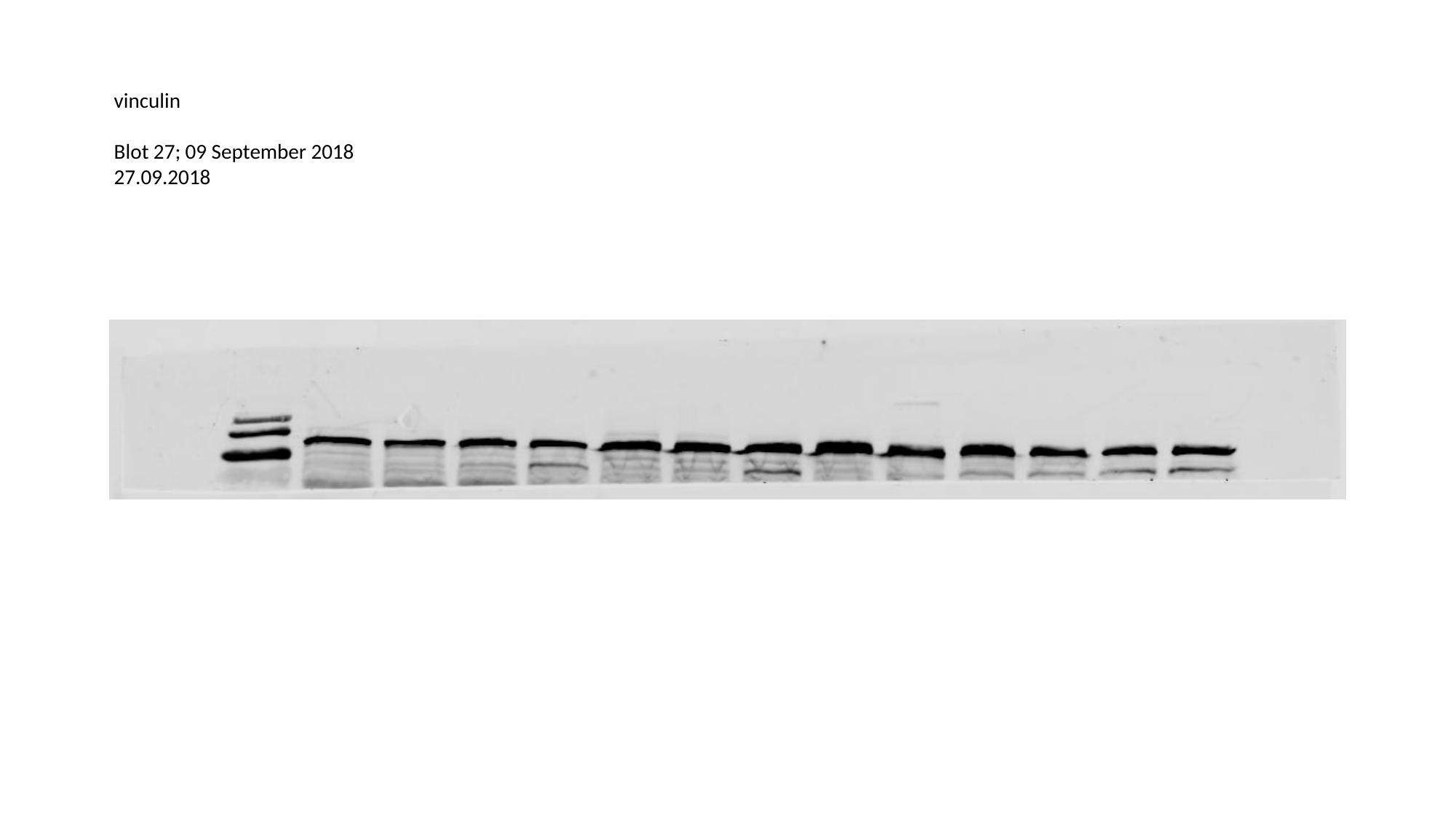

vinculin
Blot 27; 09 September 2018
27.09.2018

## Slide 15
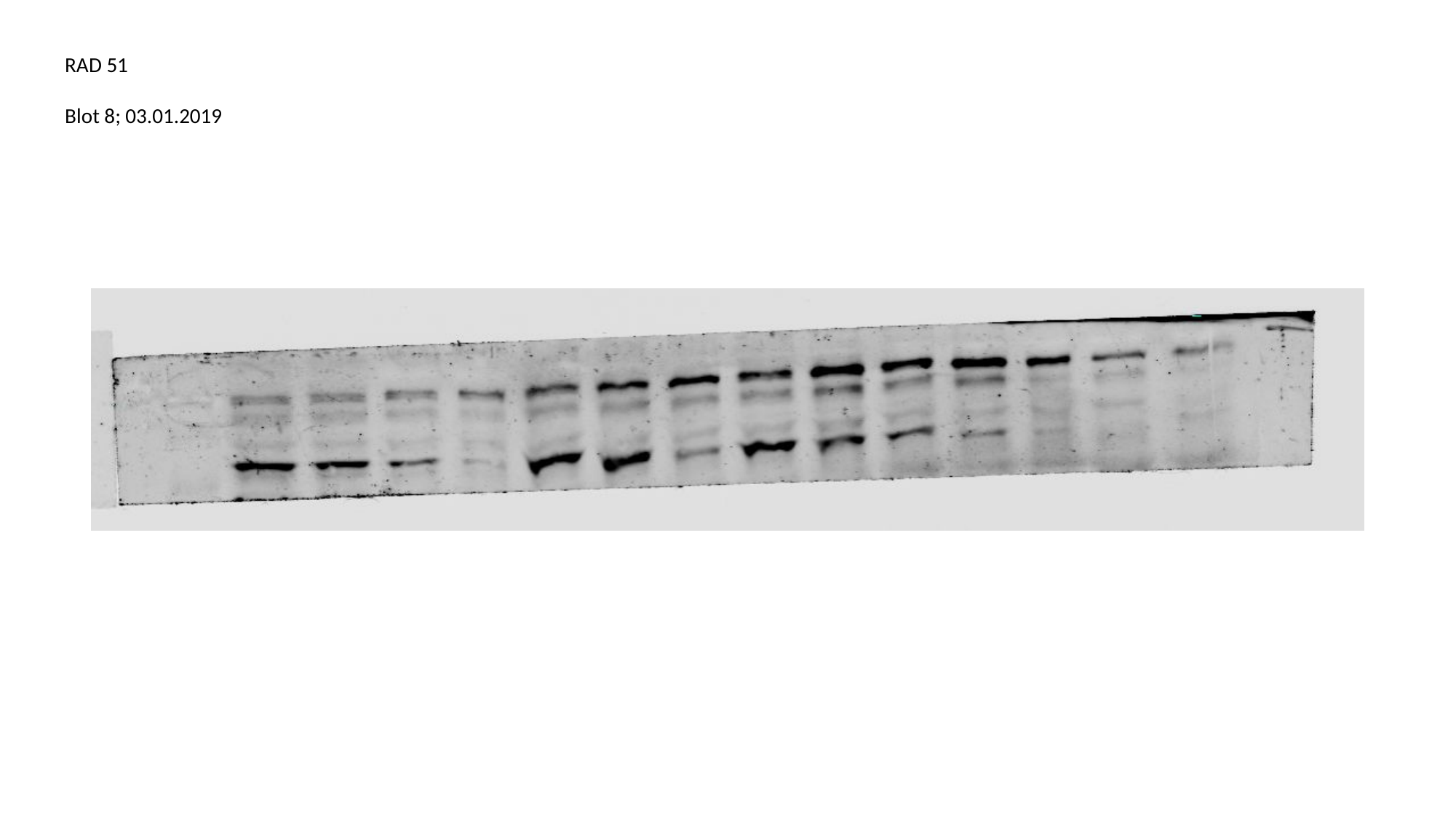

RAD 51
Blot 8; 03.01.2019

## Slide 16
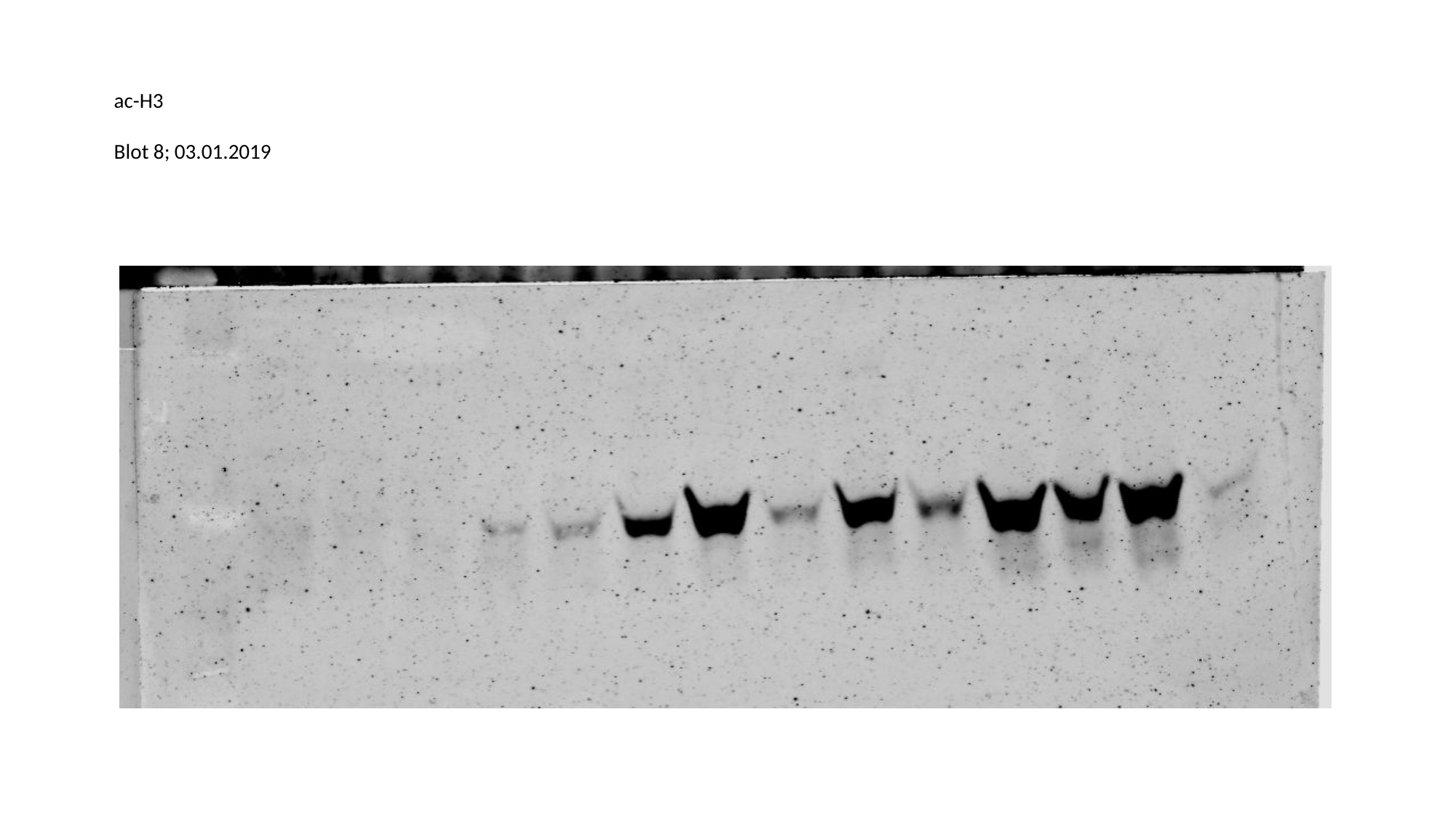

ac-H3
Blot 8; 03.01.2019

## Slide 17
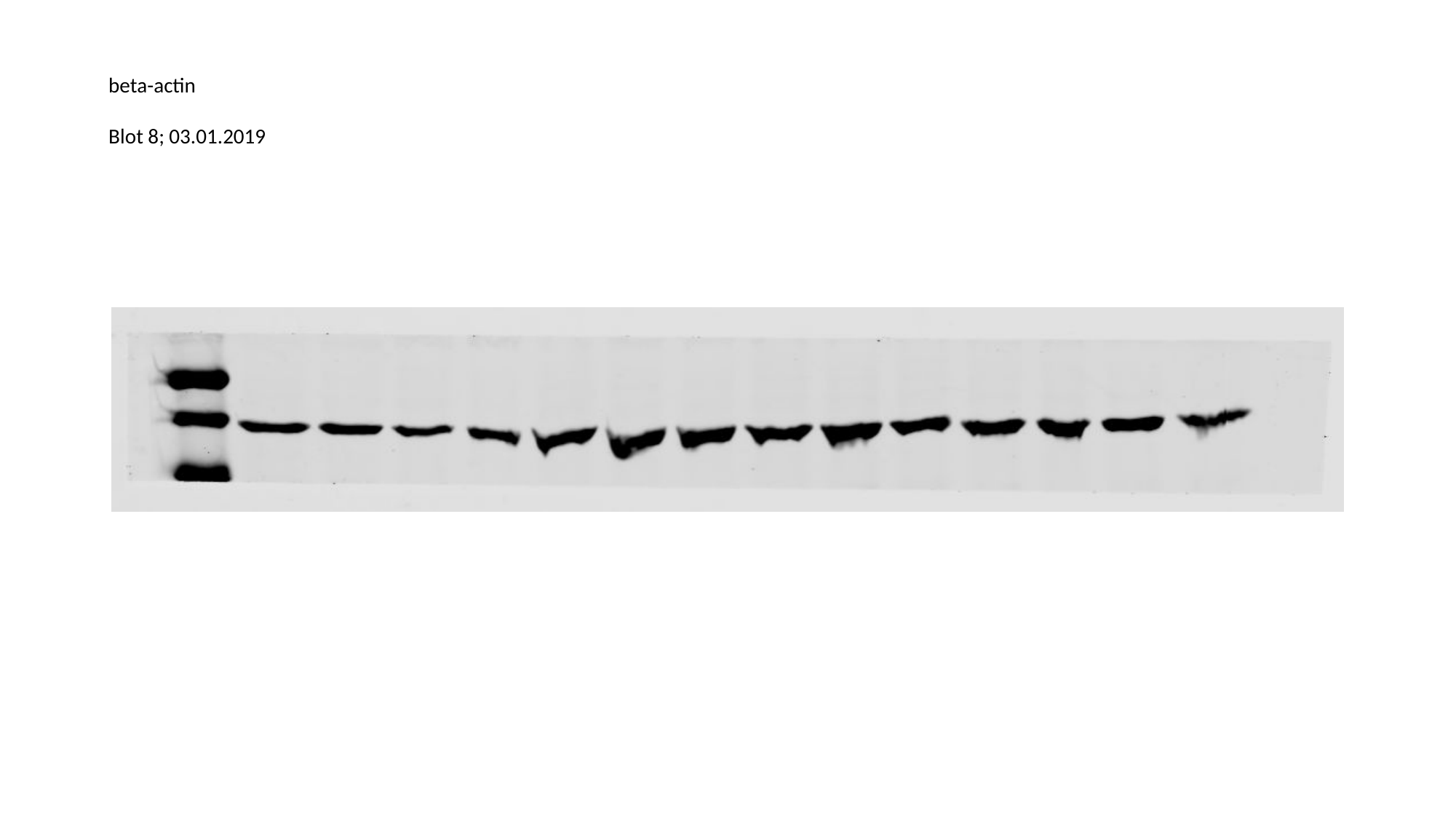

beta-actin
Blot 8; 03.01.2019

## Slide 18
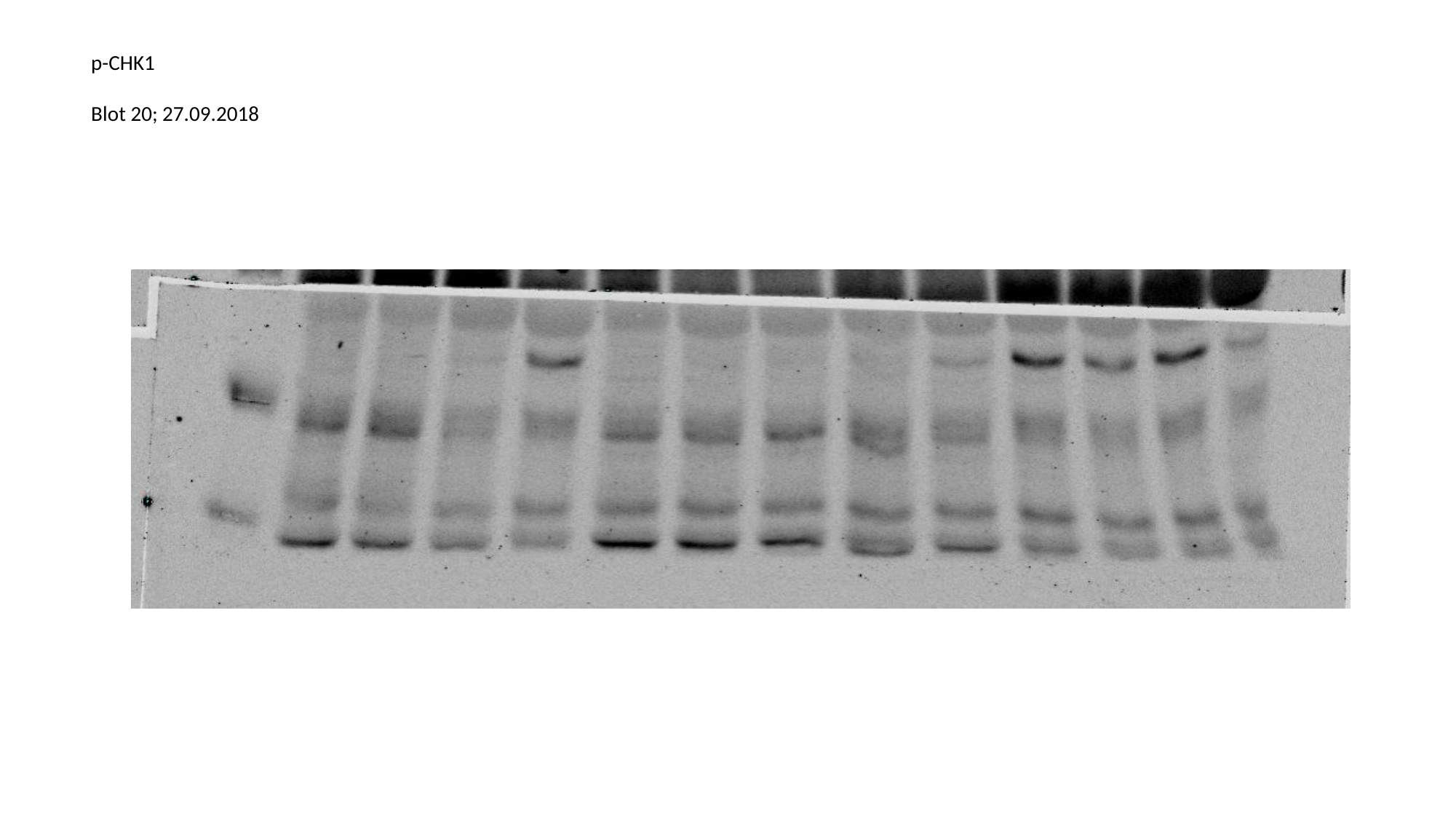

p-CHK1
Blot 20; 27.09.2018

## Slide 19
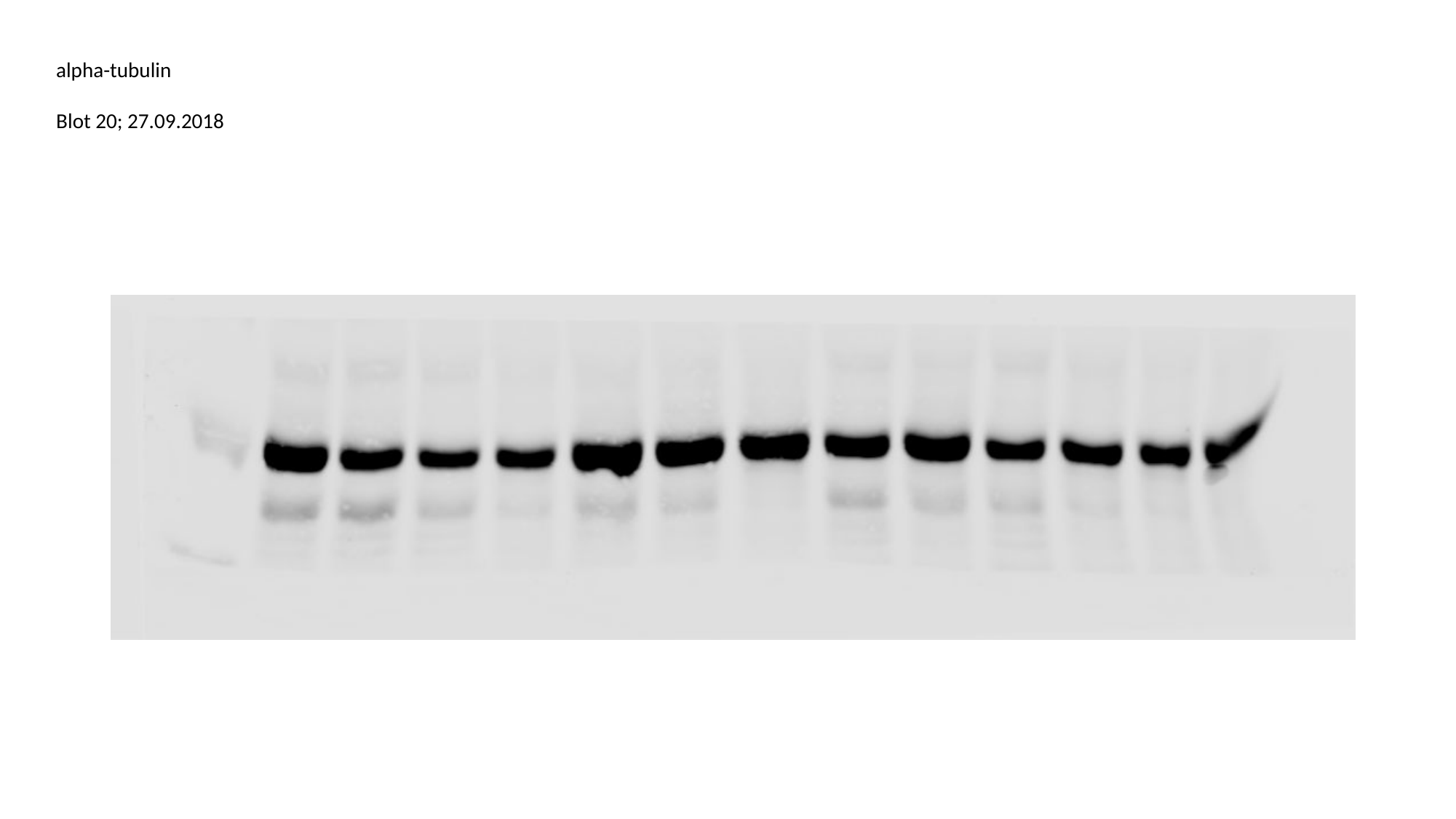

alpha-tubulin
Blot 20; 27.09.2018

## Slide 20
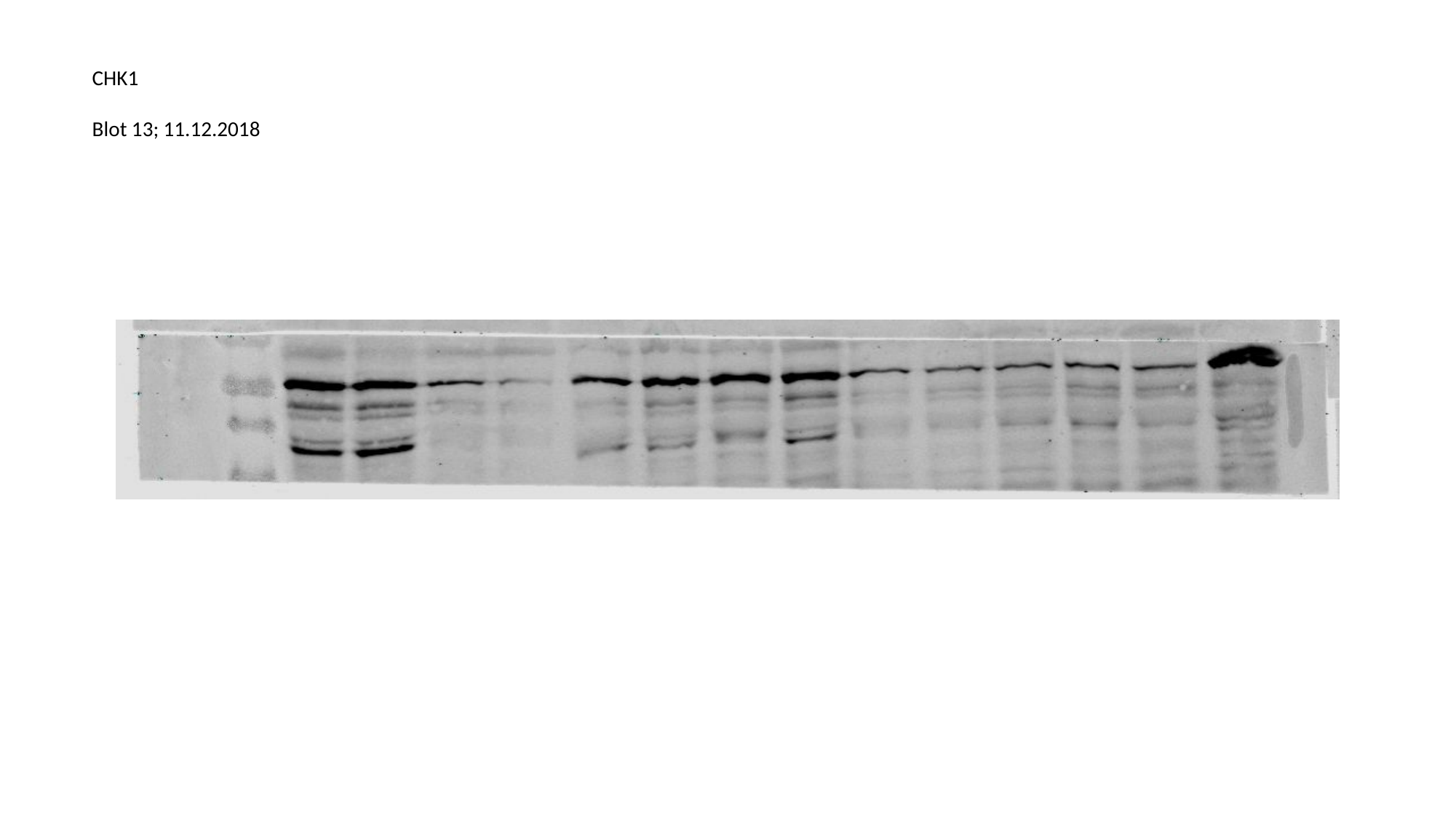

CHK1
Blot 13; 11.12.2018

## Slide 21
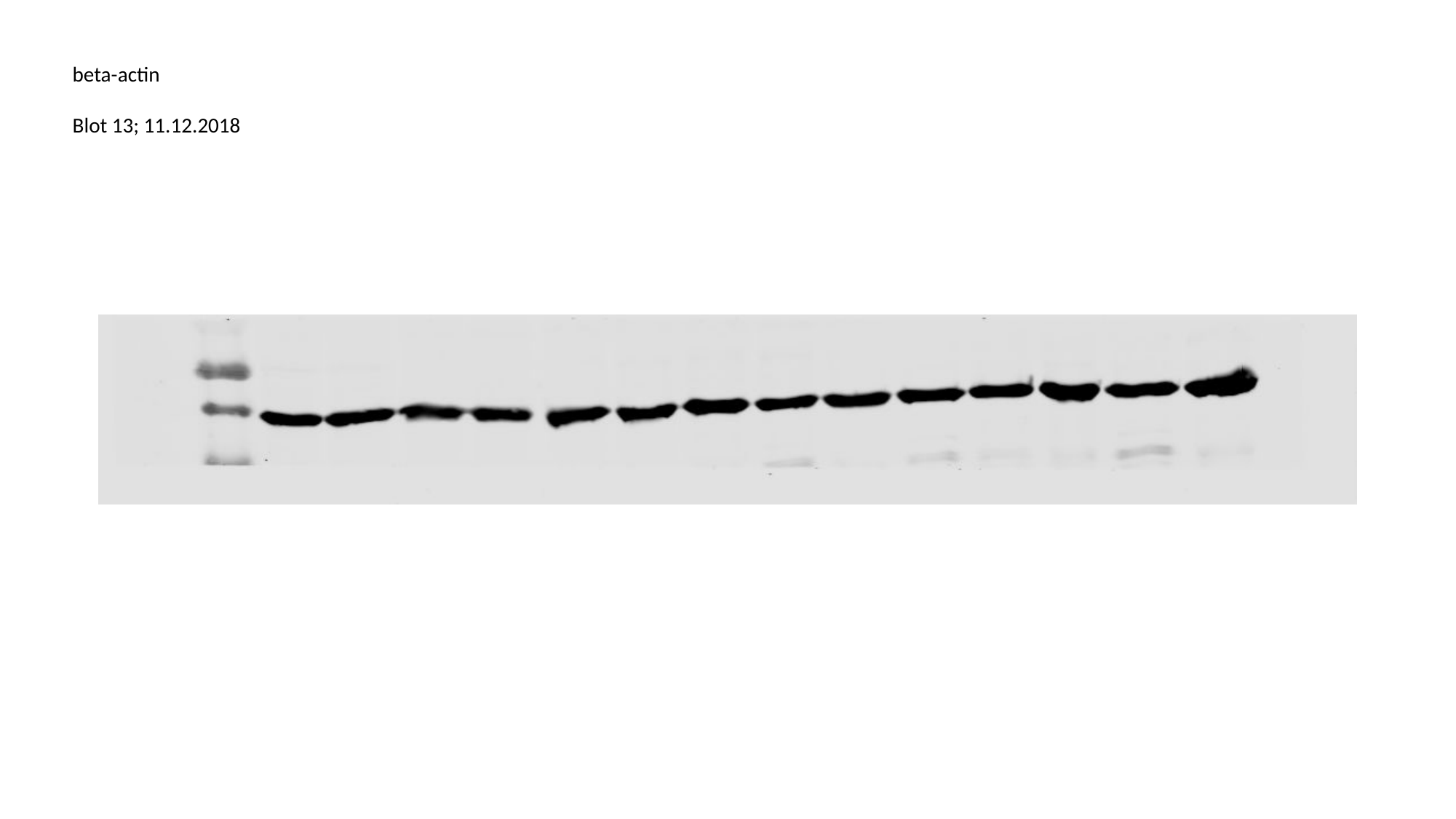

beta-actin
Blot 13; 11.12.2018

## Slide 22
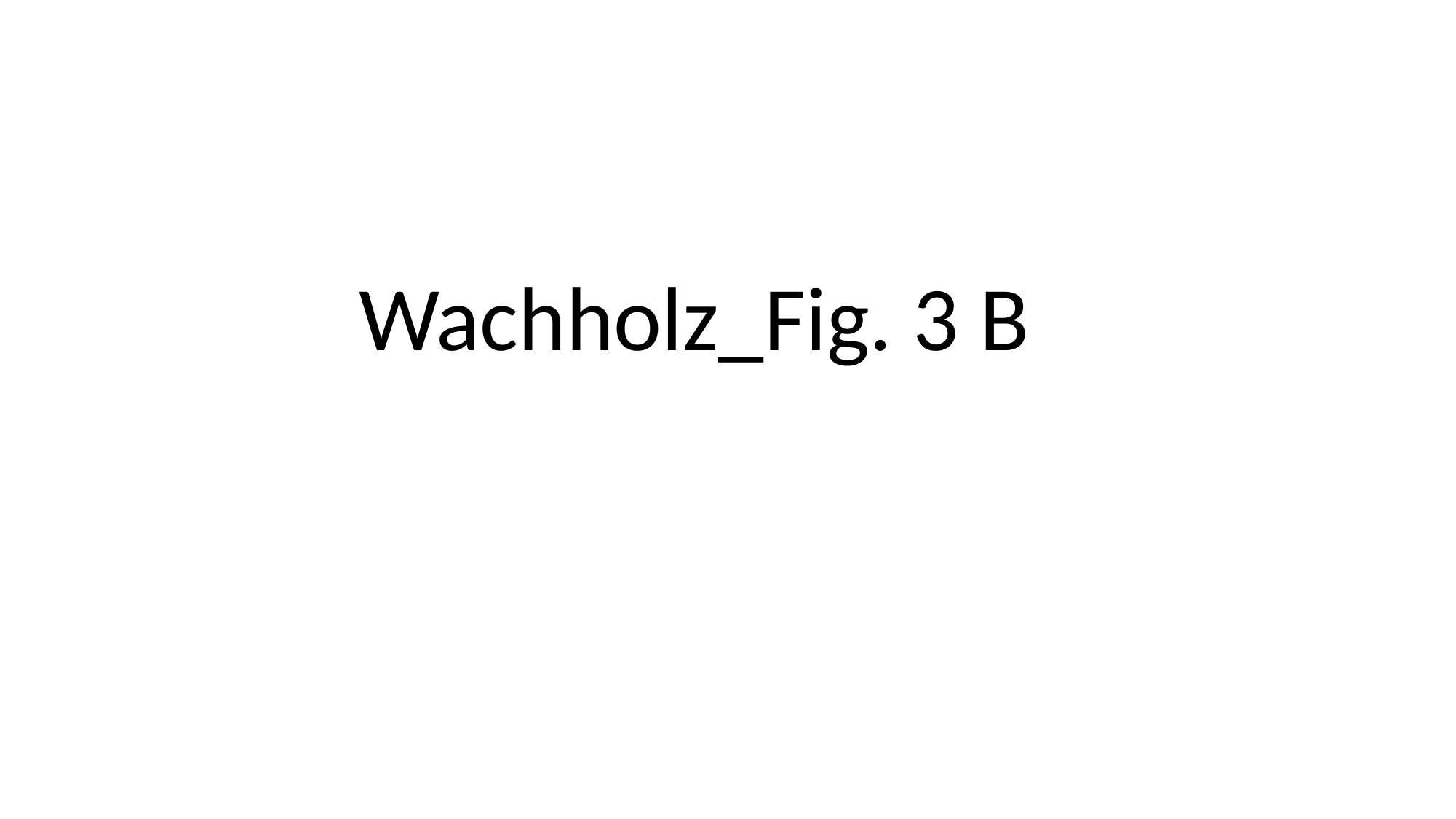

Wachholz_Fig. 3 B

## Slide 23
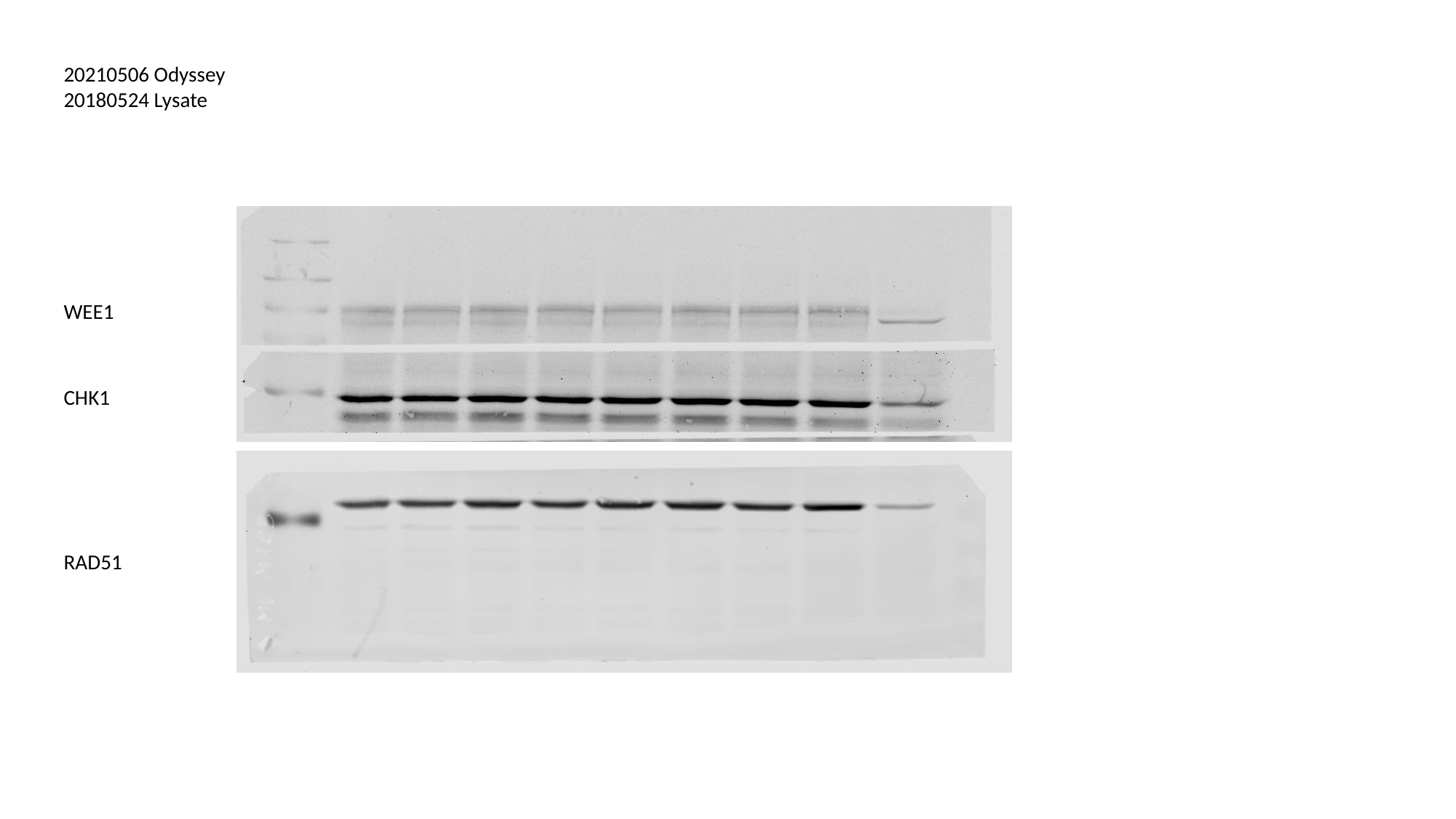

20210506 Odyssey
20180524 Lysate
WEE1
CHK1
RAD51

## Slide 24
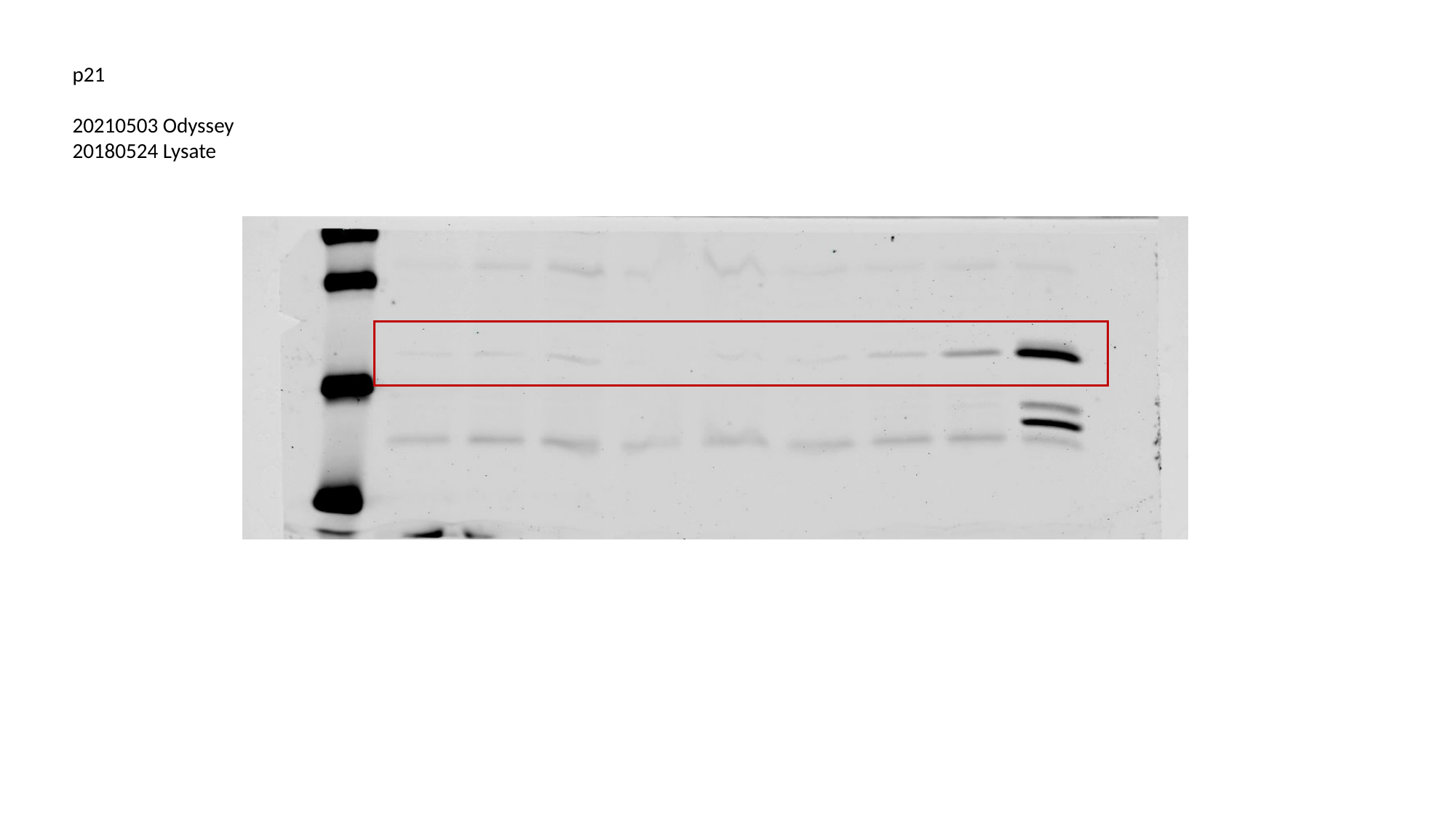

p21
20210503 Odyssey
20180524 Lysate

## Slide 25
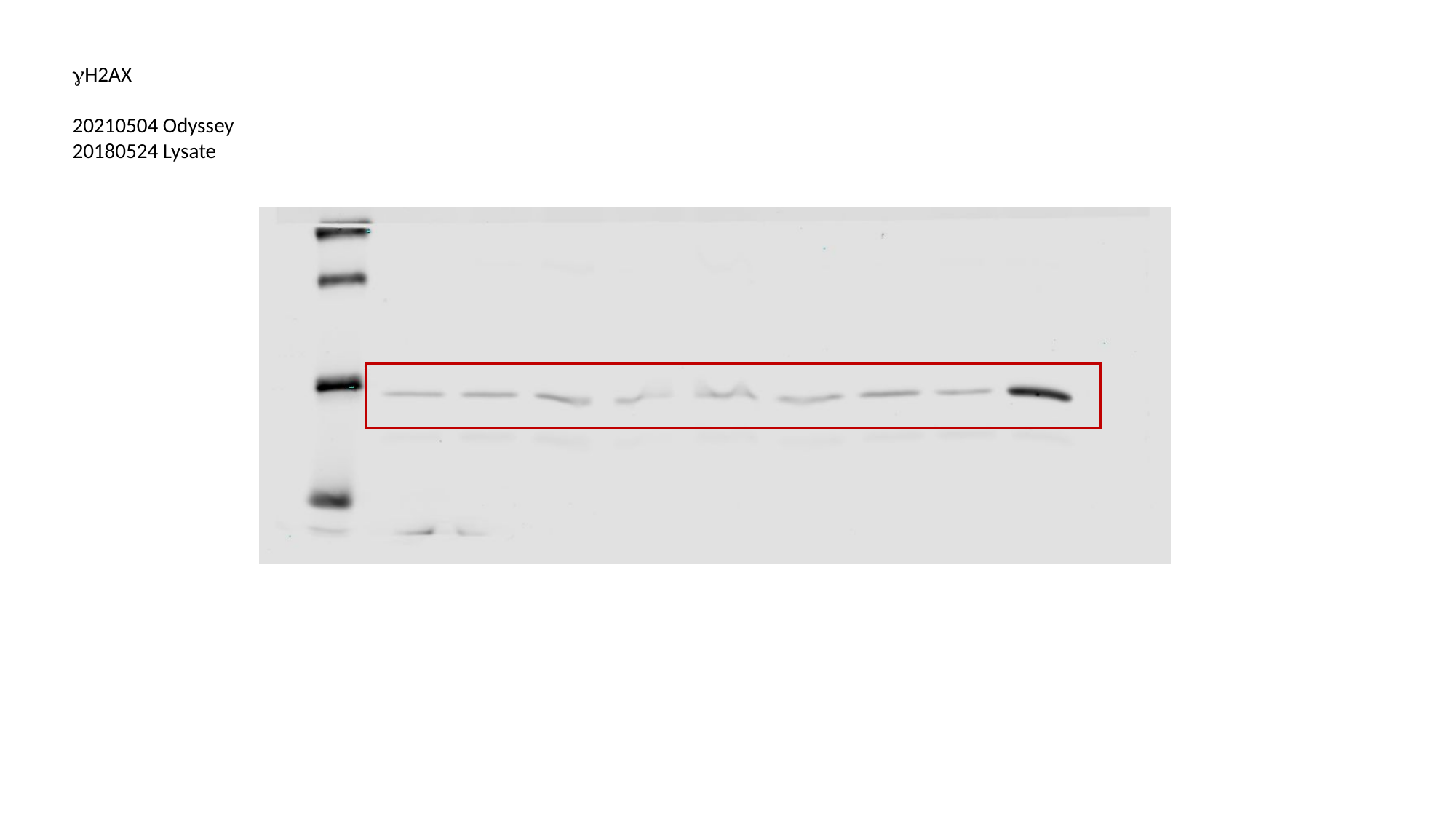

γH2AX
20210504 Odyssey
20180524 Lysate

## Slide 26
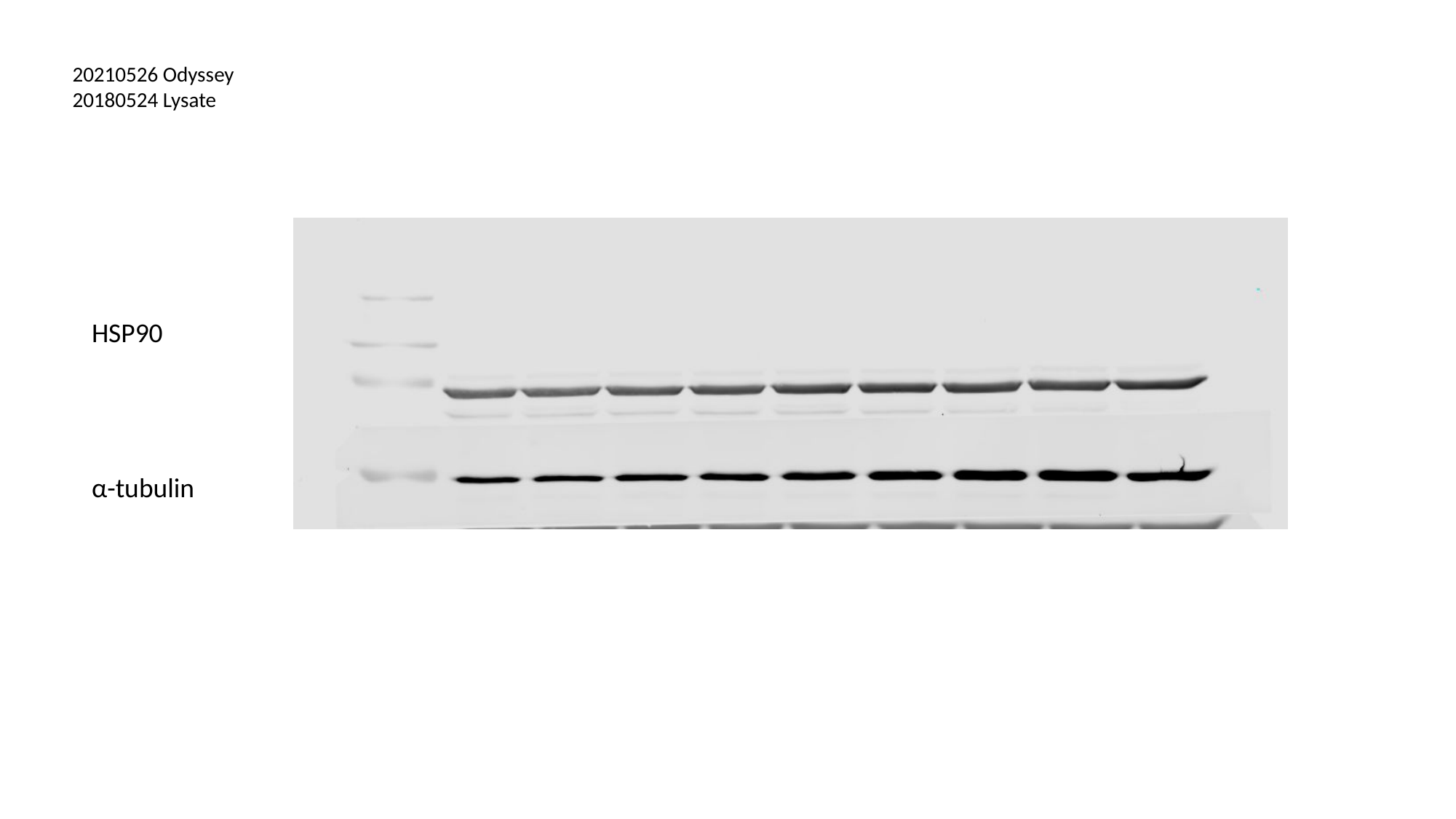

20210526 Odyssey
20180524 Lysate
HSP90
α-tubulin

## Slide 27
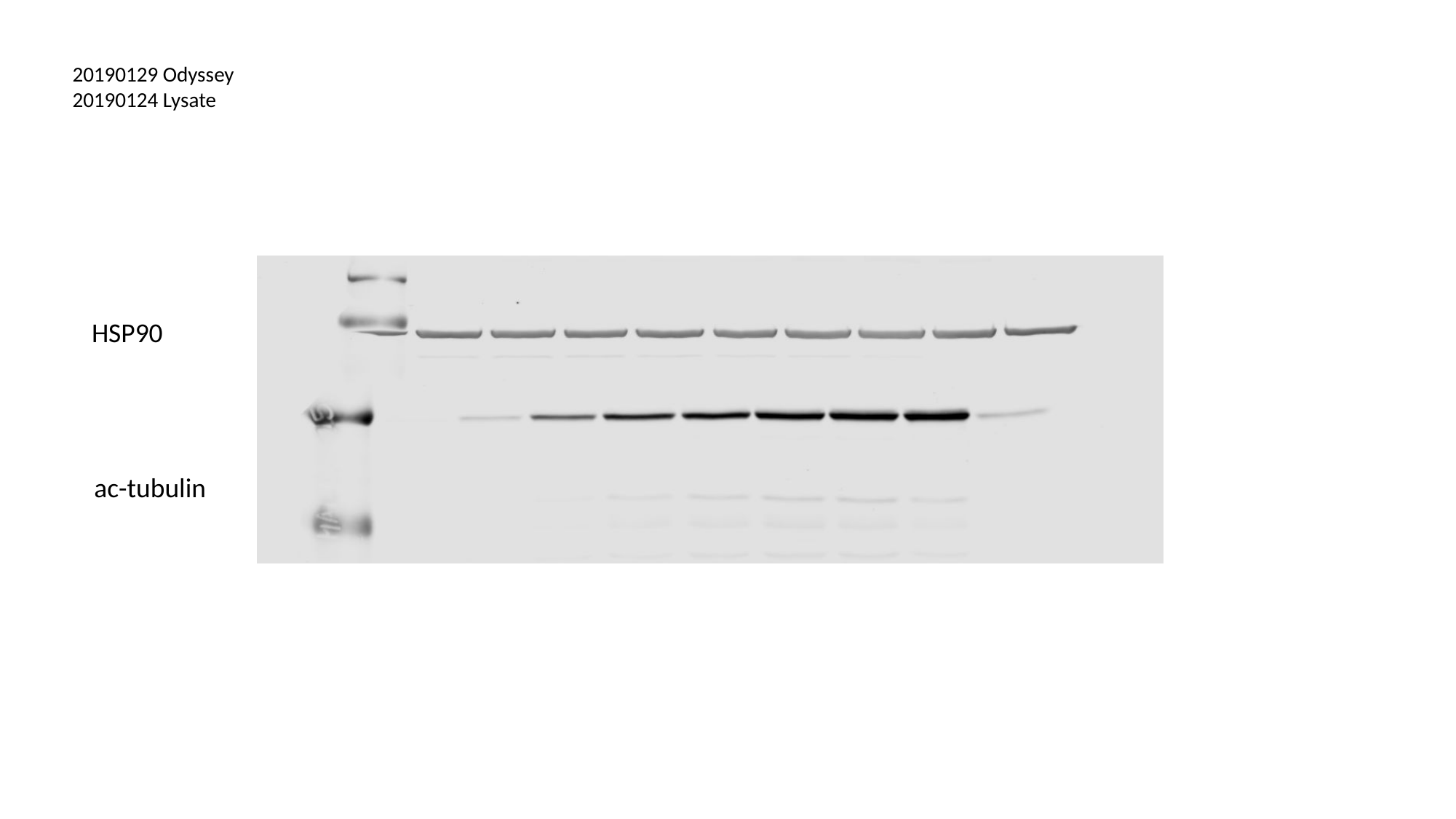

20190129 Odyssey
20190124 Lysate
HSP90
ac-tubulin

## Slide 28
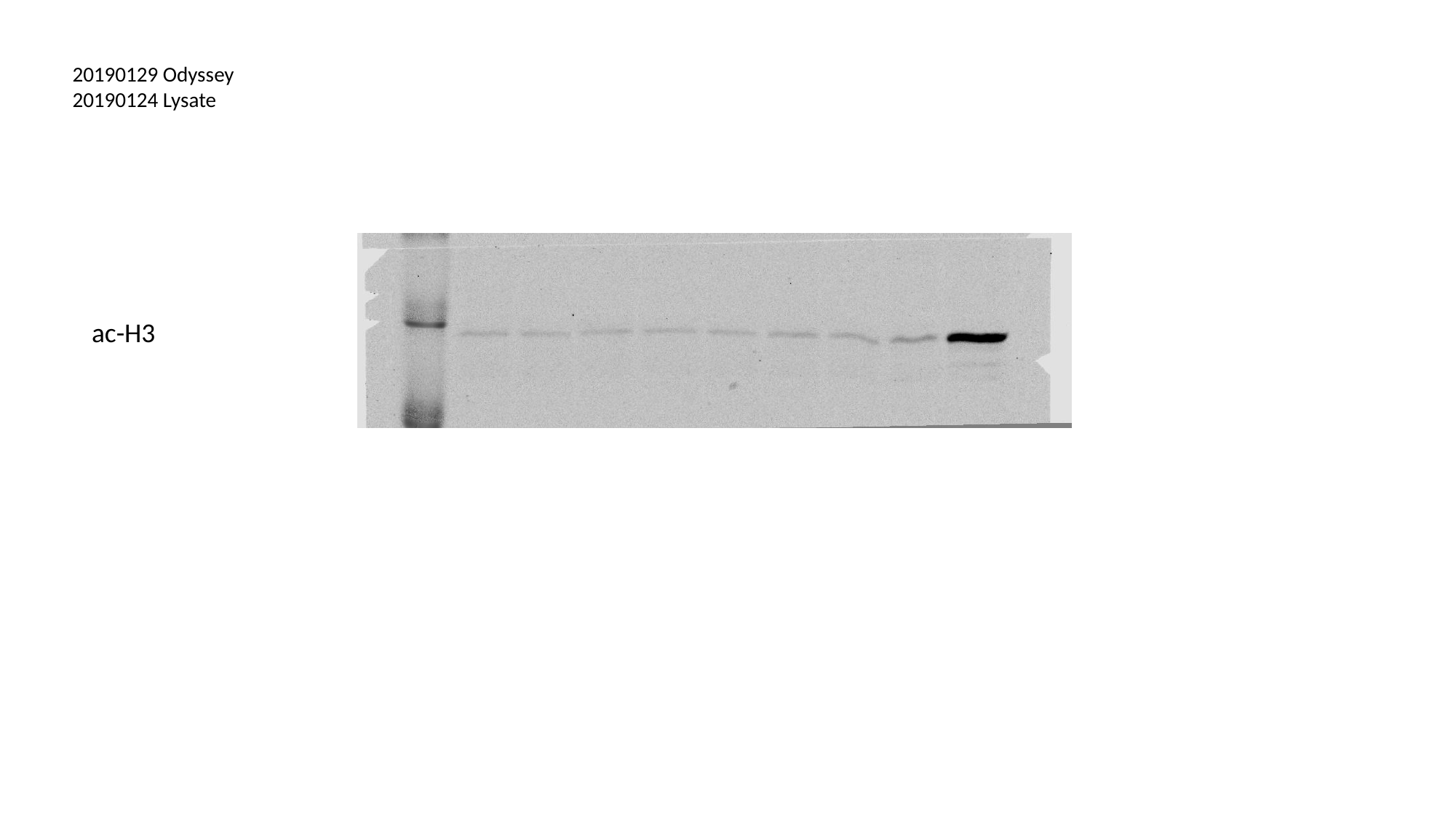

20190129 Odyssey
20190124 Lysate
ac-H3

## Slide 29
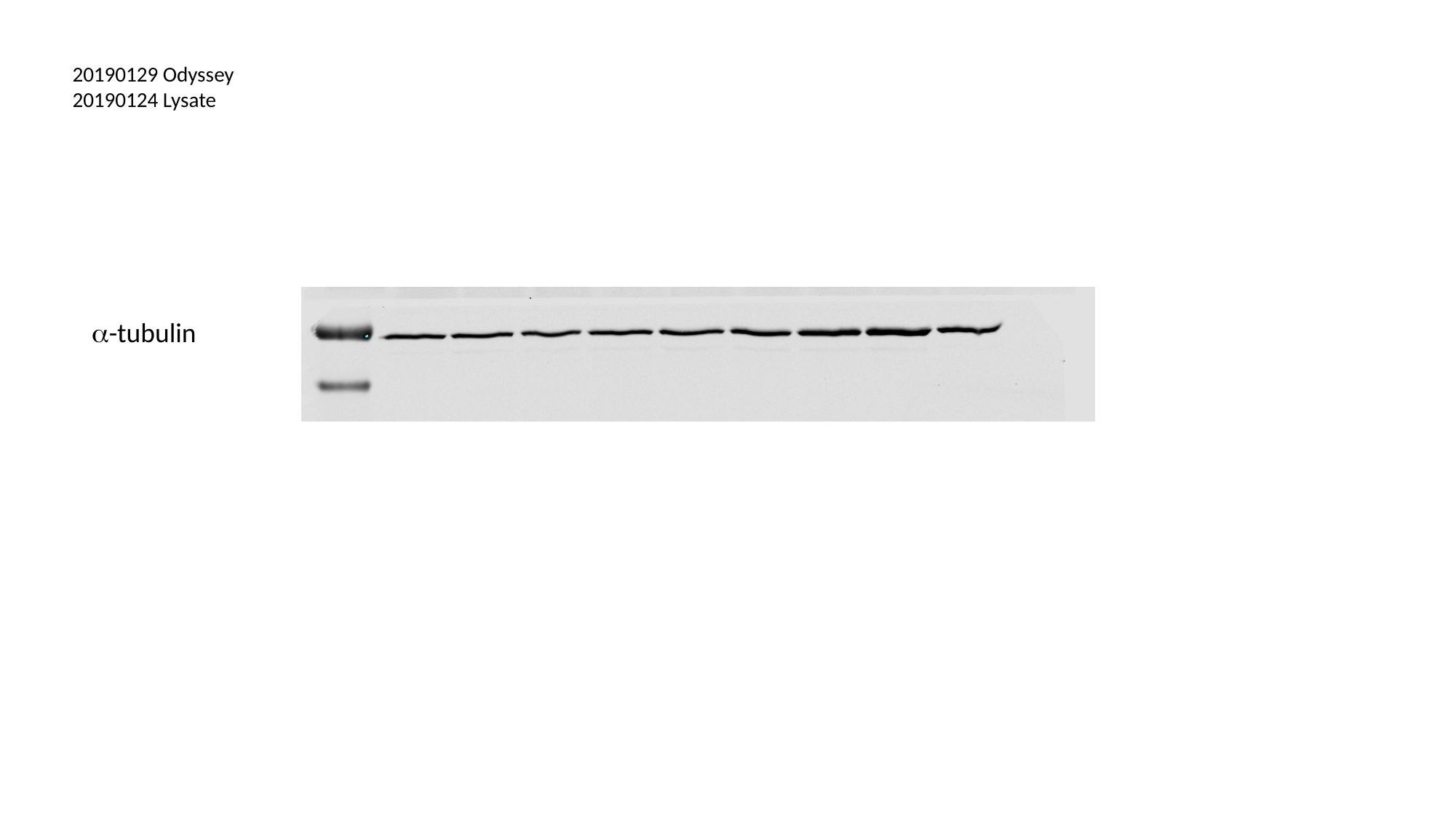

20190129 Odyssey
20190124 Lysate
a-tubulin

## Slide 30
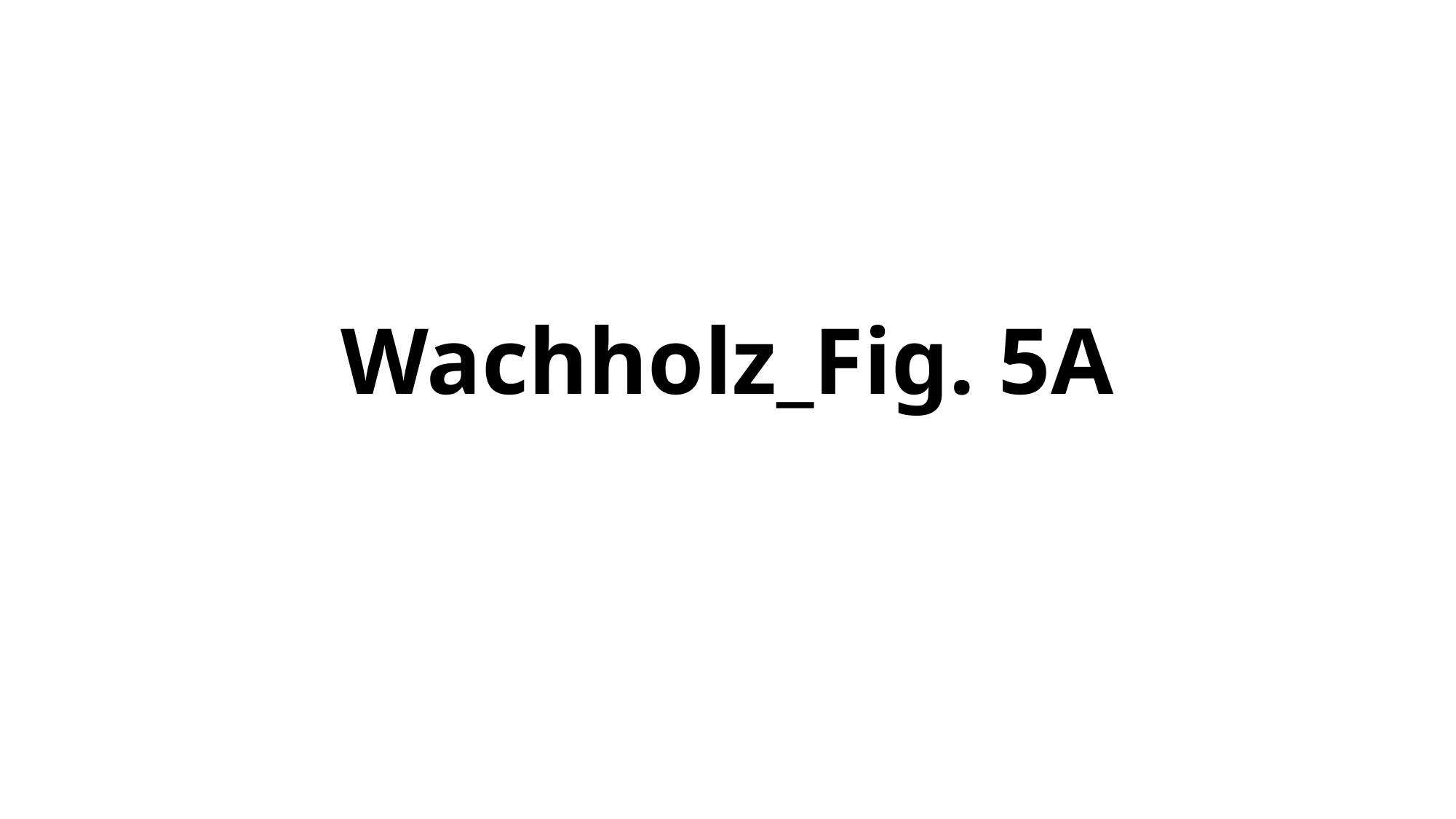

# Wachholz_Fig. 5A

## Slide 31
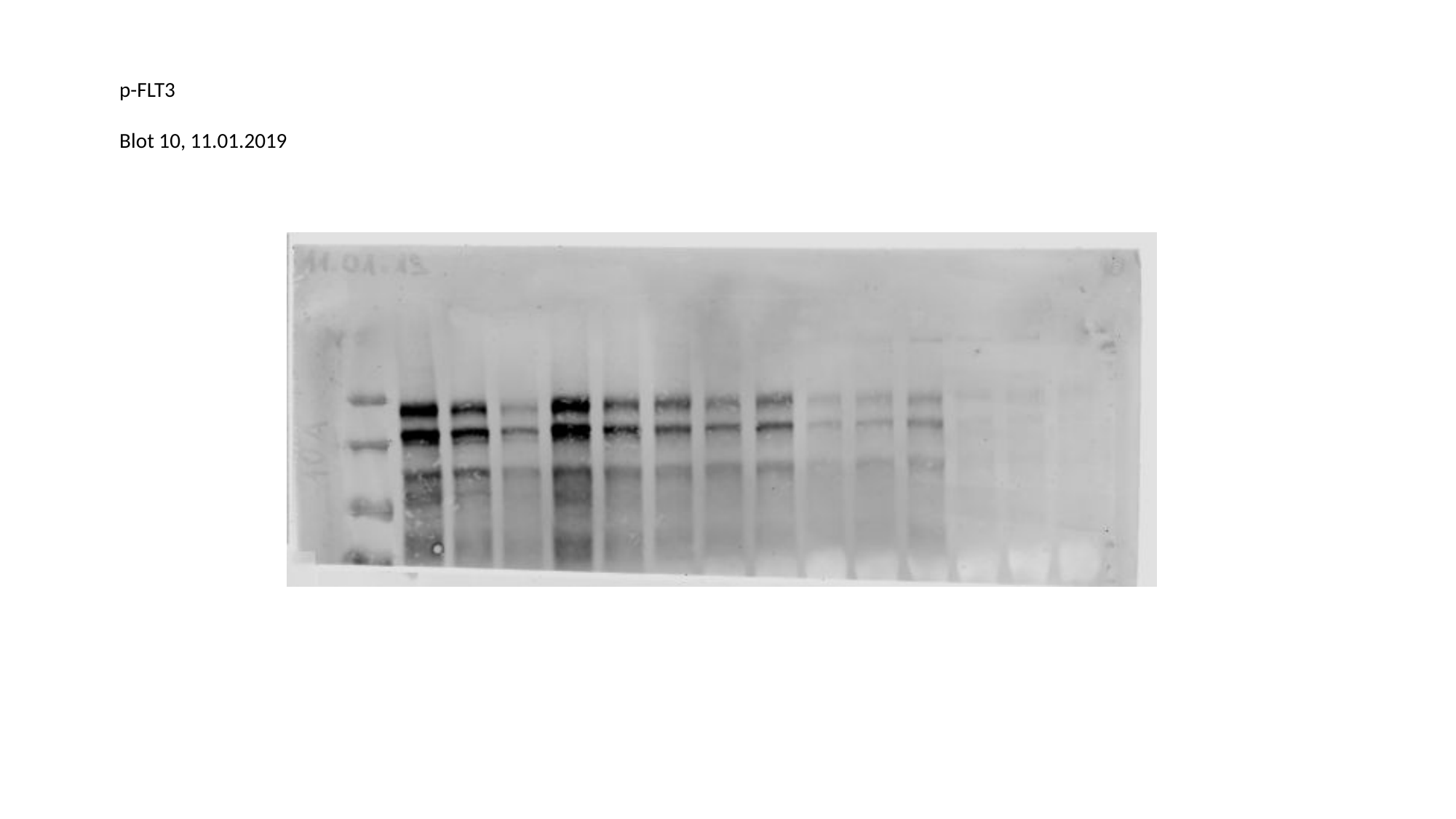

p-FLT3
Blot 10, 11.01.2019

## Slide 32
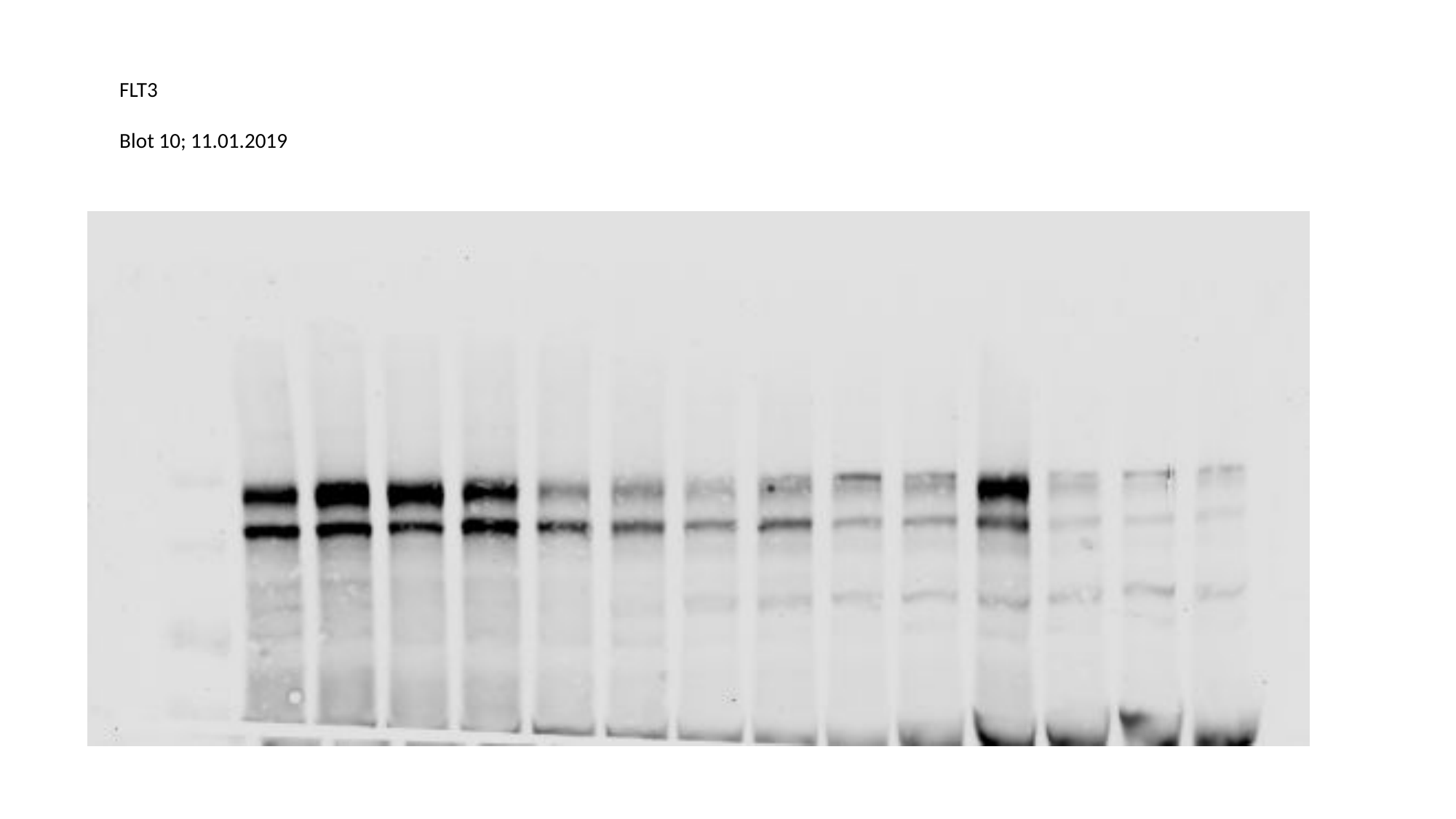

FLT3
Blot 10; 11.01.2019

## Slide 33
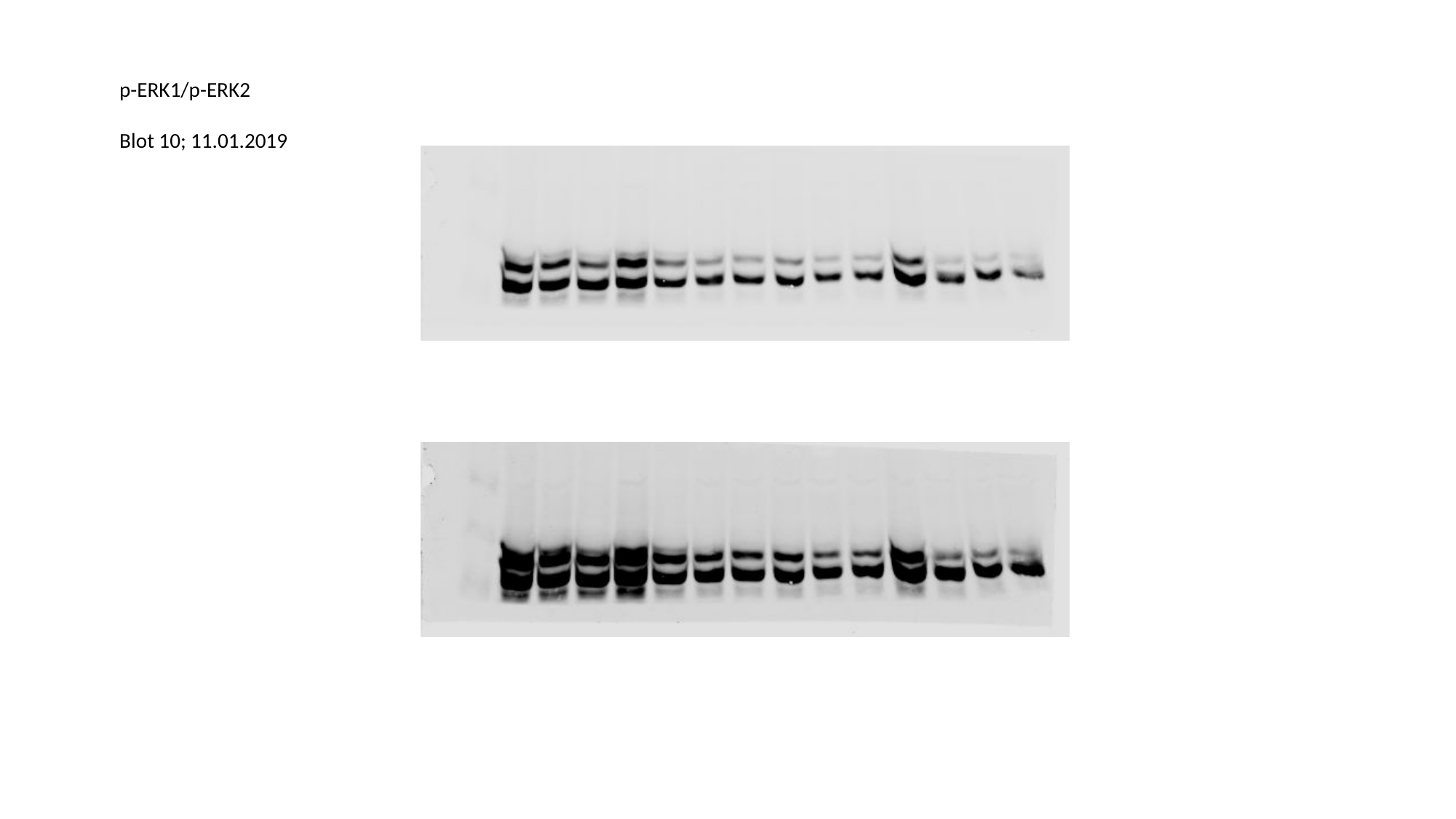

p-ERK1/p-ERK2
Blot 10; 11.01.2019

## Slide 34
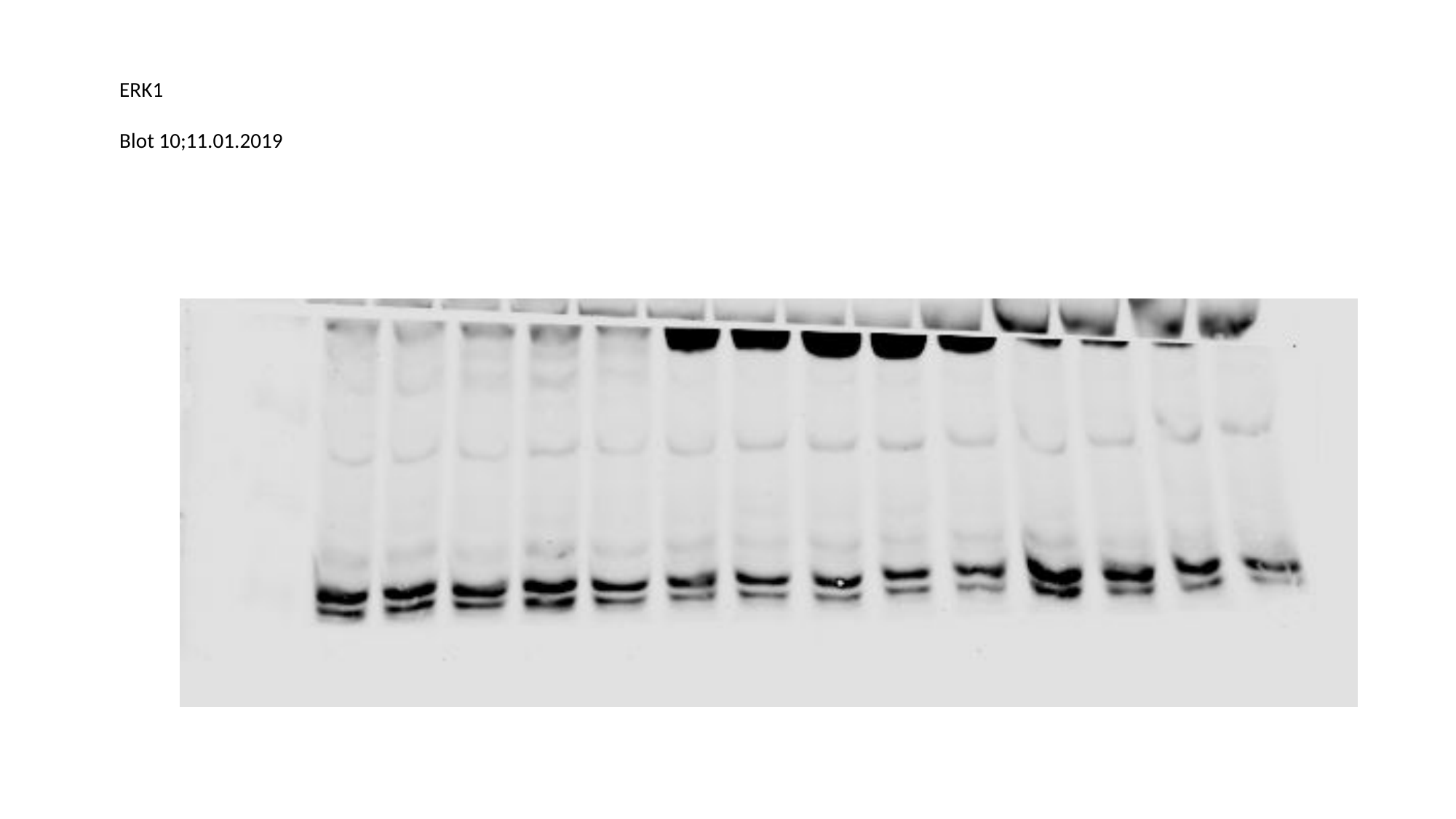

ERK1
Blot 10;11.01.2019

## Slide 35
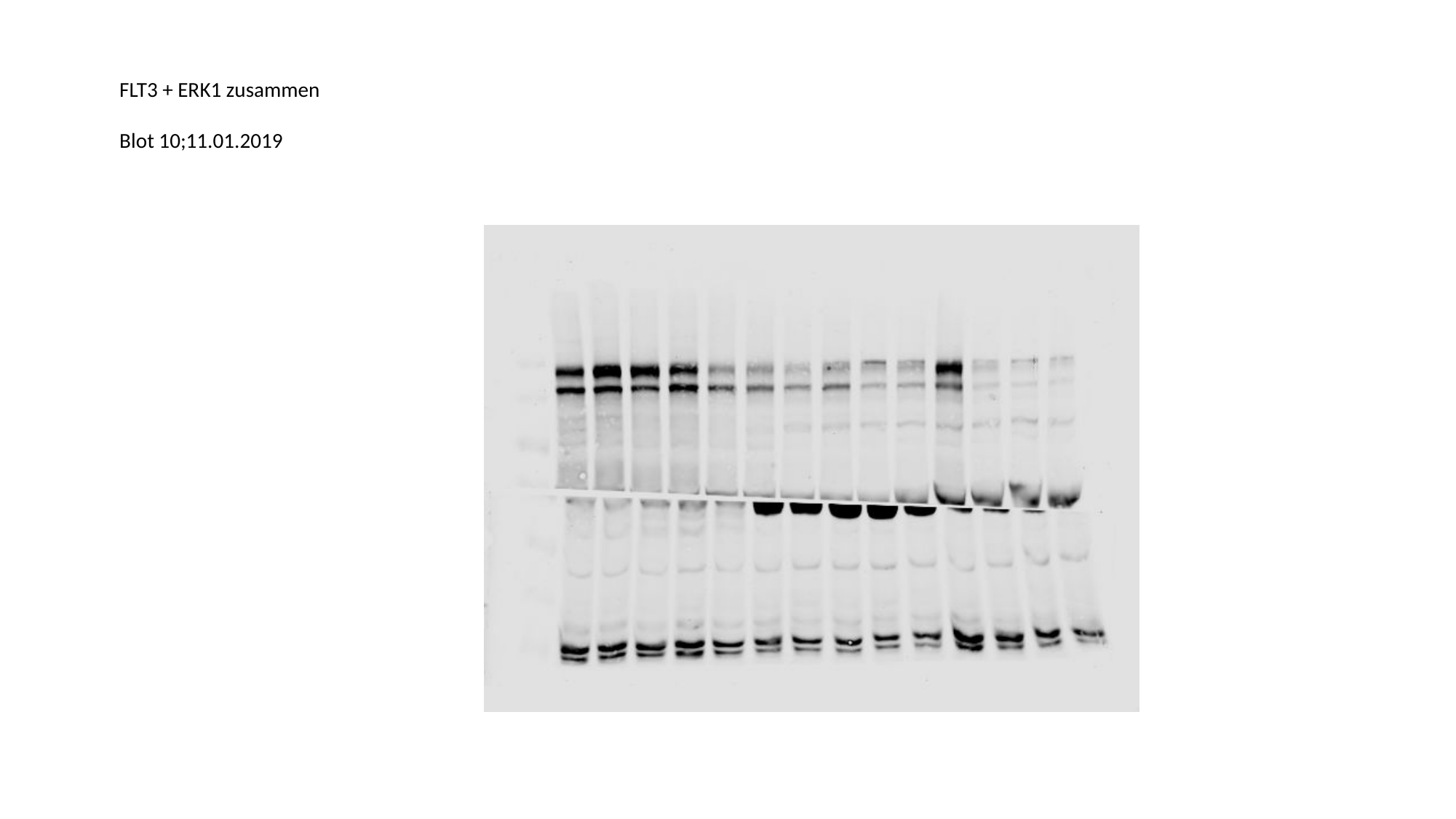

FLT3 + ERK1 zusammen
Blot 10;11.01.2019

## Slide 36
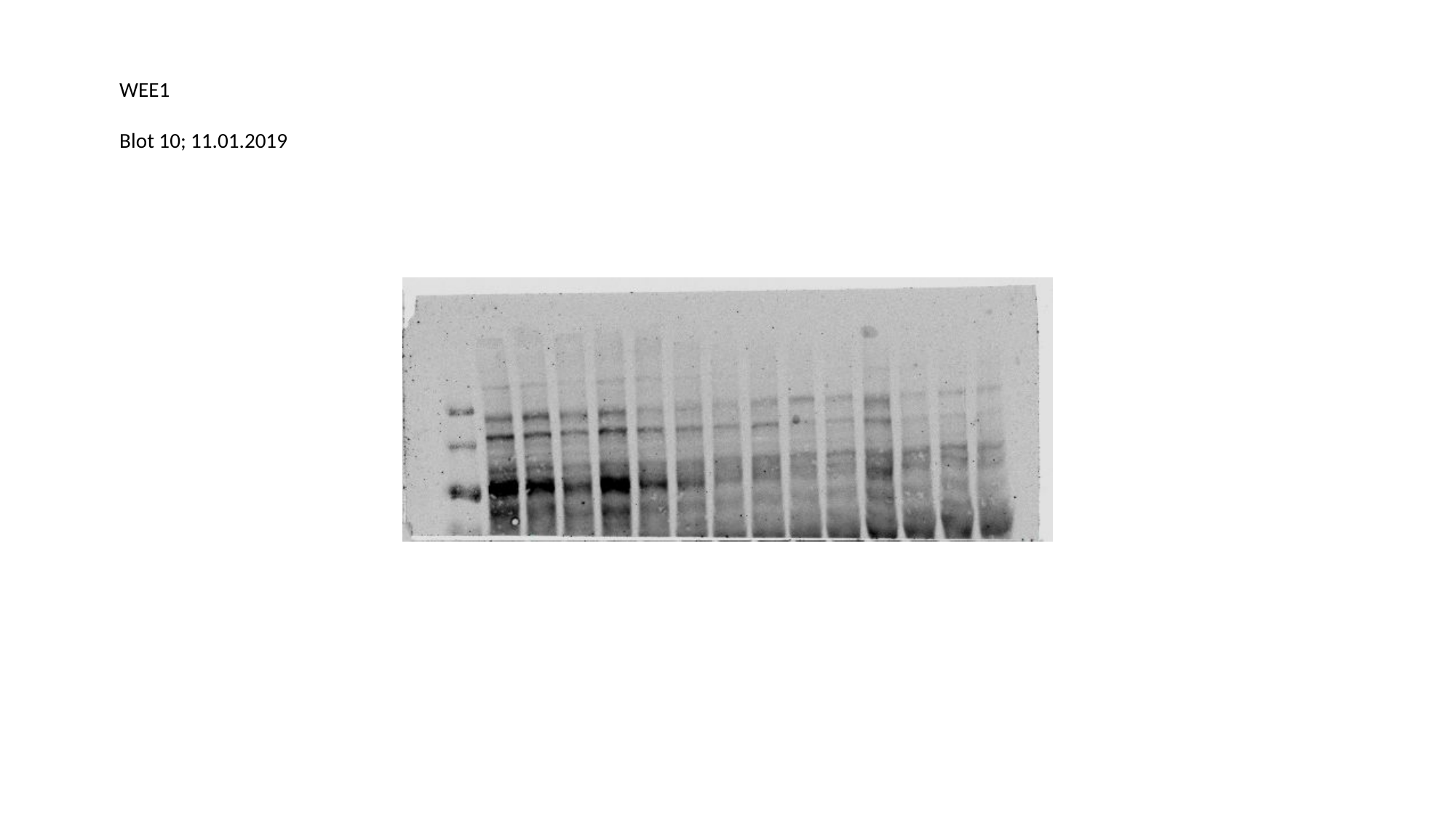

WEE1
Blot 10; 11.01.2019

## Slide 37
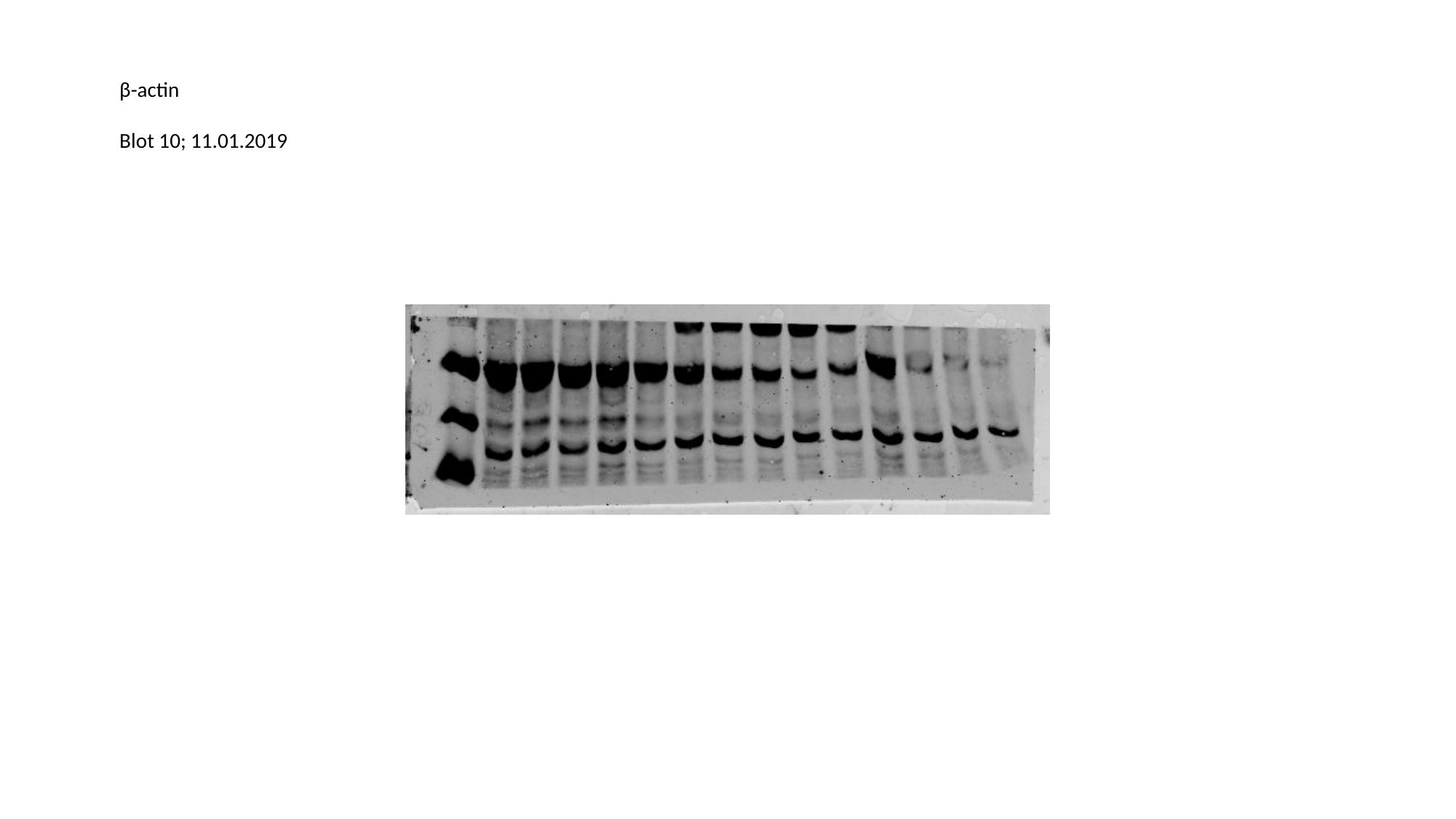

β-actin
Blot 10; 11.01.2019

## Slide 38
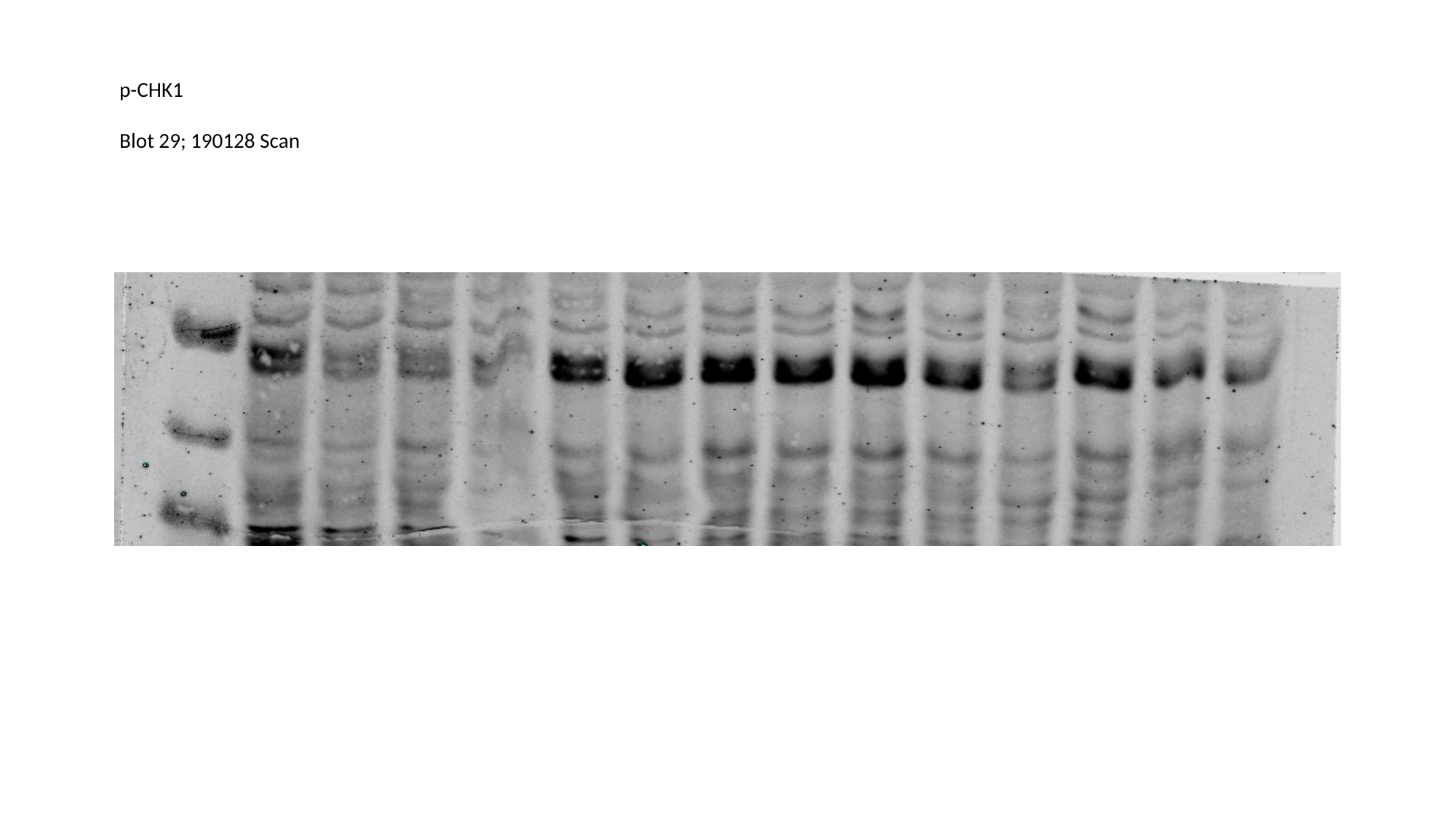

p-CHK1
Blot 29; 190128 Scan

## Slide 39
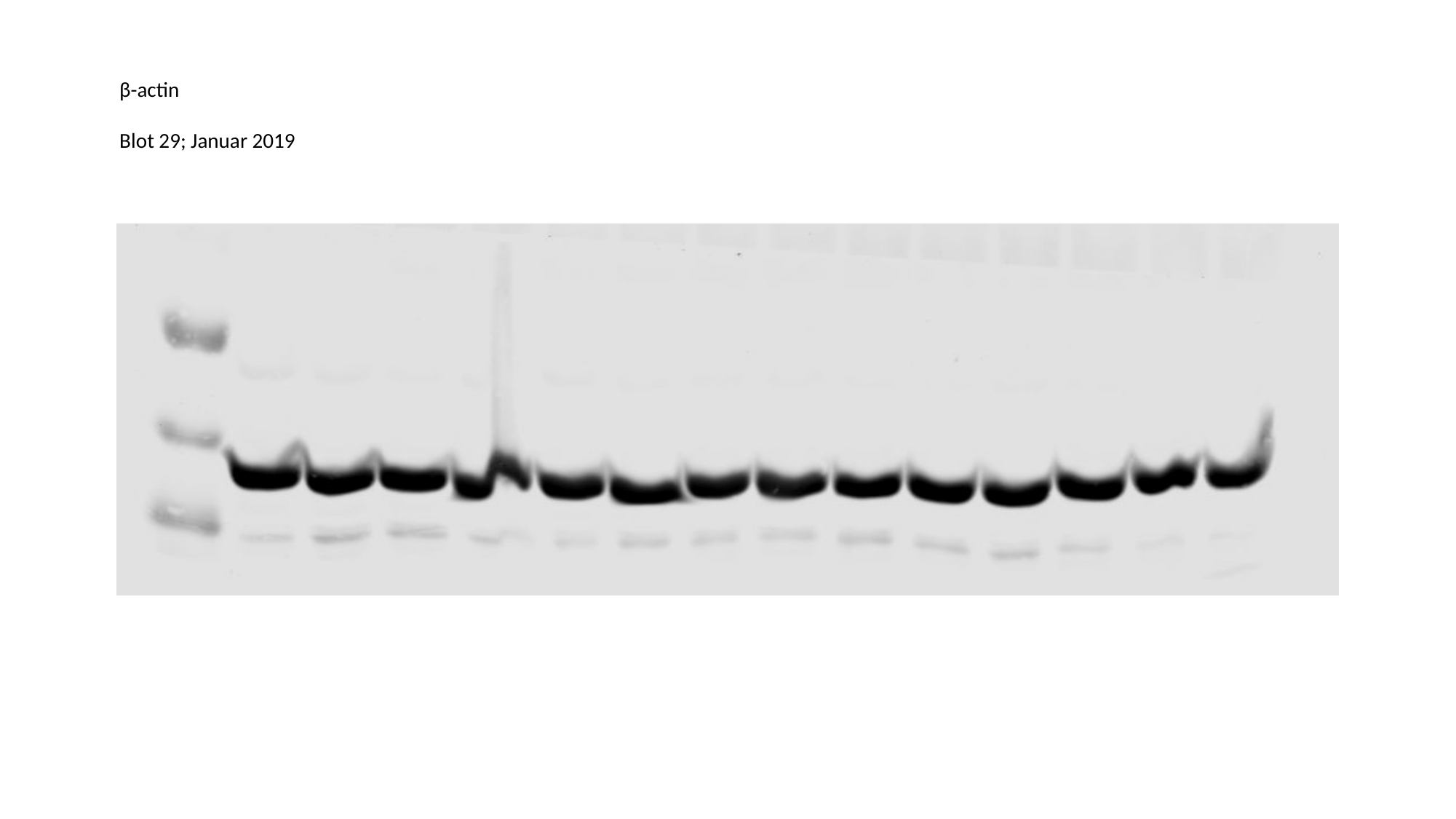

β-actin
Blot 29; Januar 2019

## Slide 40
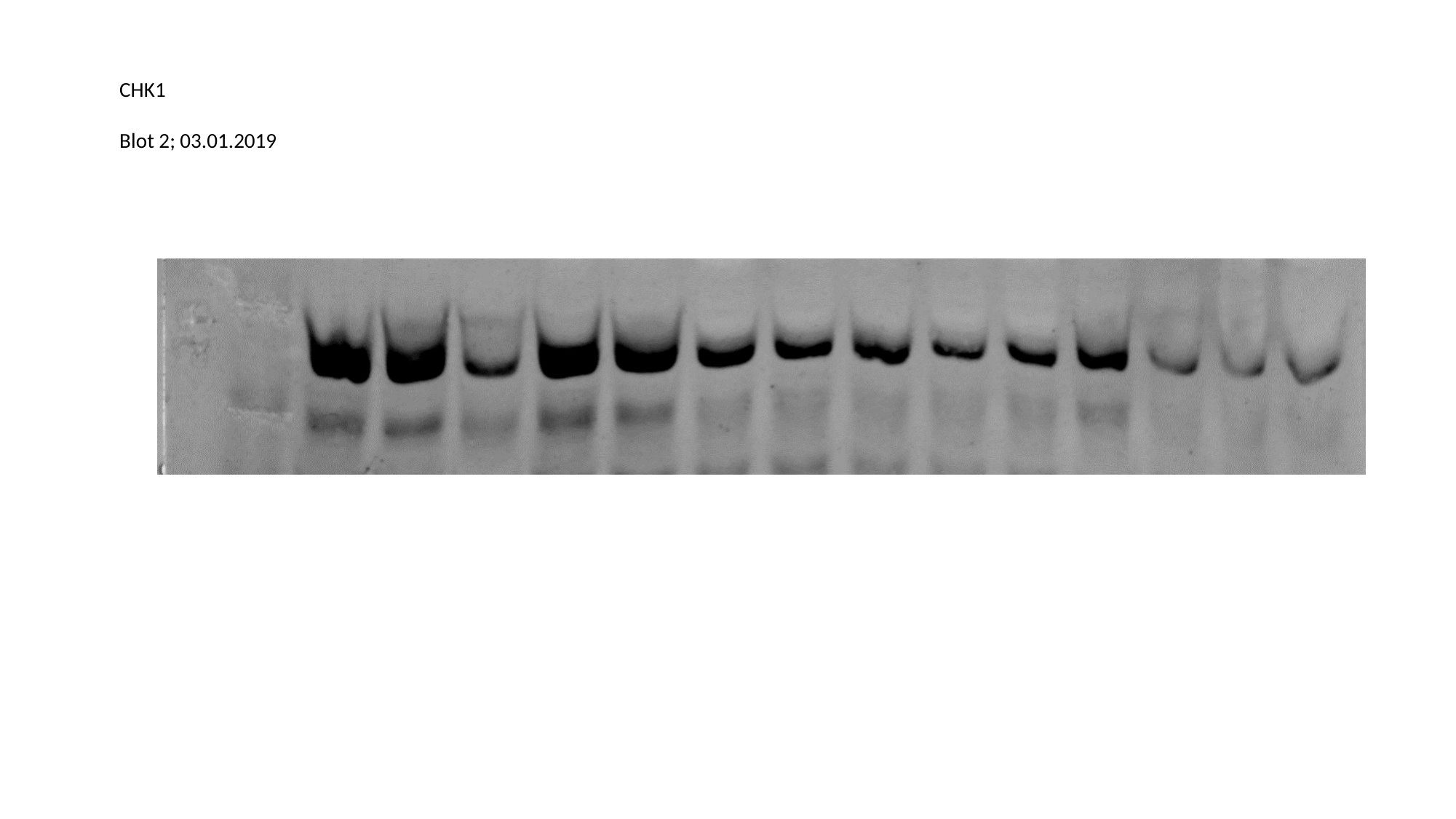

CHK1
Blot 2; 03.01.2019

## Slide 41
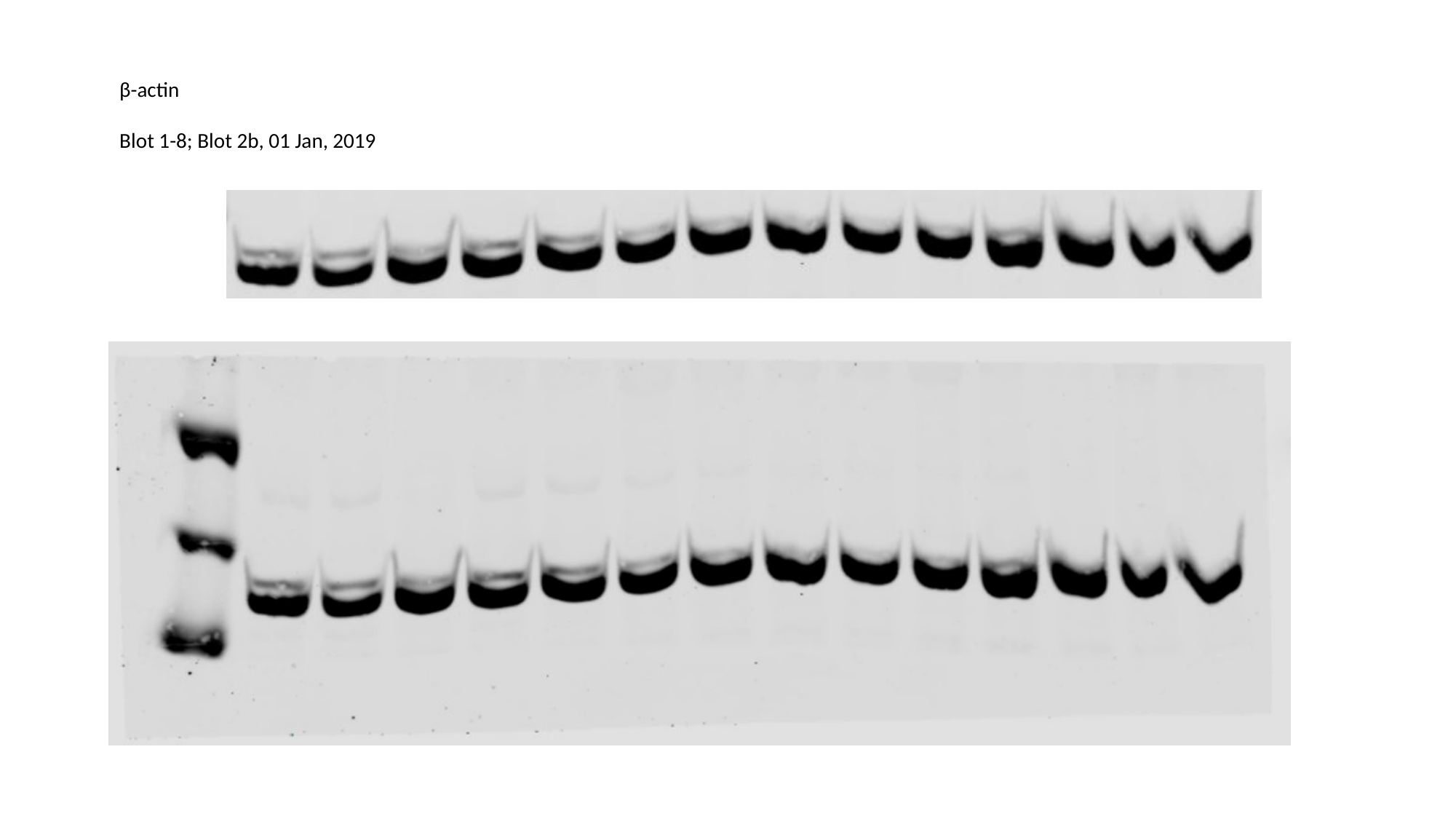

β-actin
Blot 1-8; Blot 2b, 01 Jan, 2019

## Slide 42
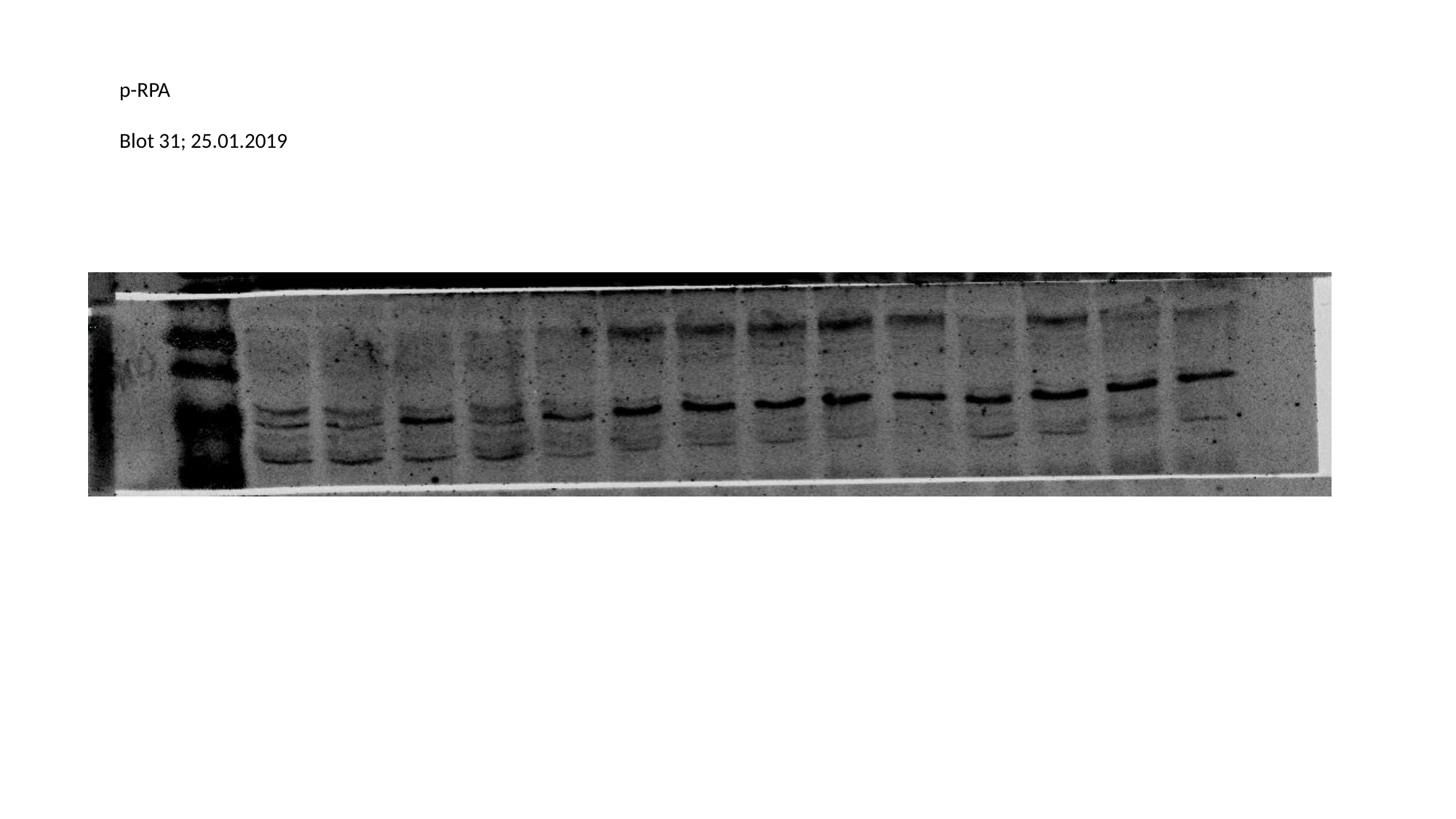

p-RPA
Blot 31; 25.01.2019

## Slide 43
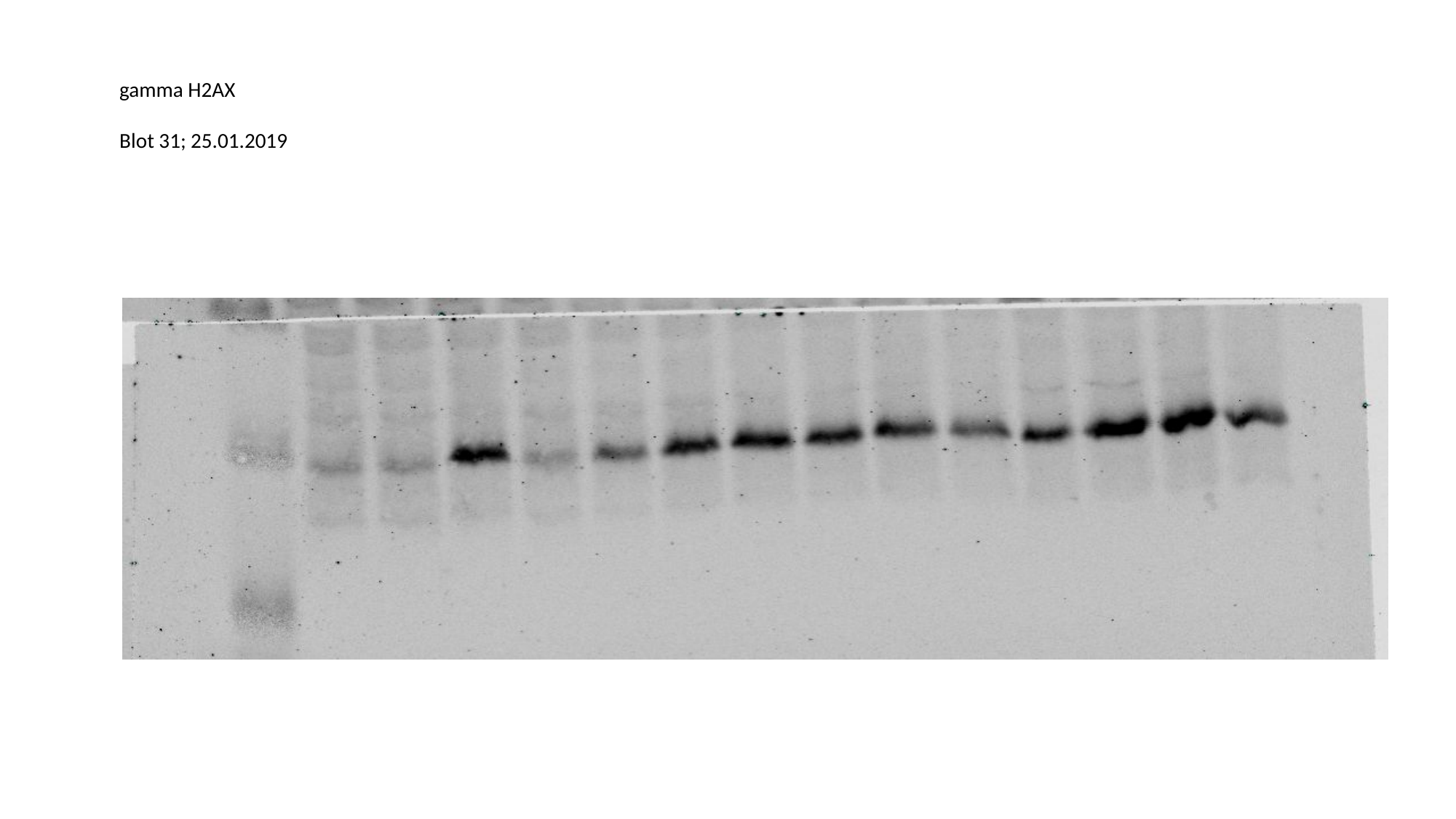

gamma H2AX
Blot 31; 25.01.2019

## Slide 44
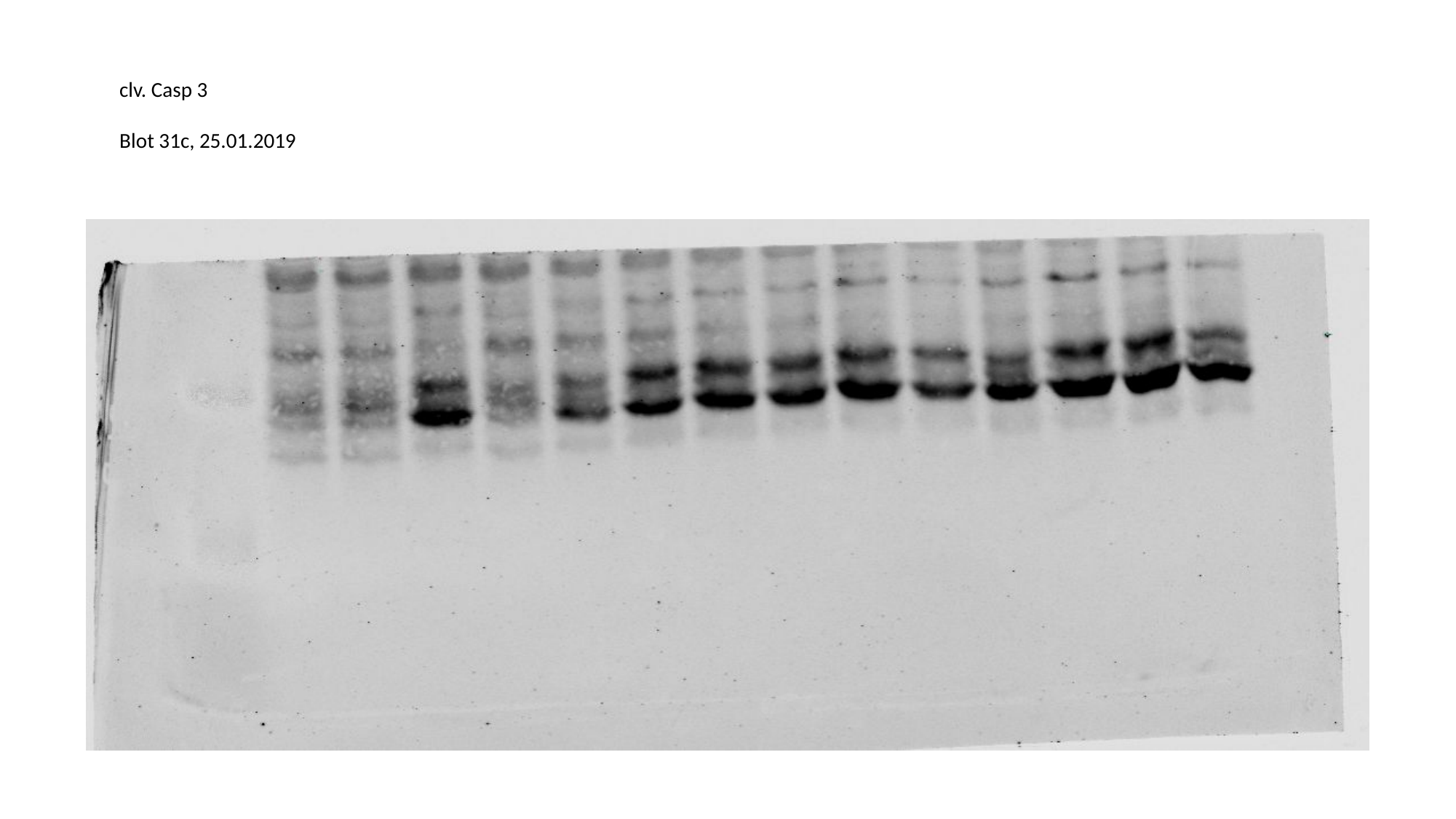

clv. Casp 3
Blot 31c, 25.01.2019

## Slide 45
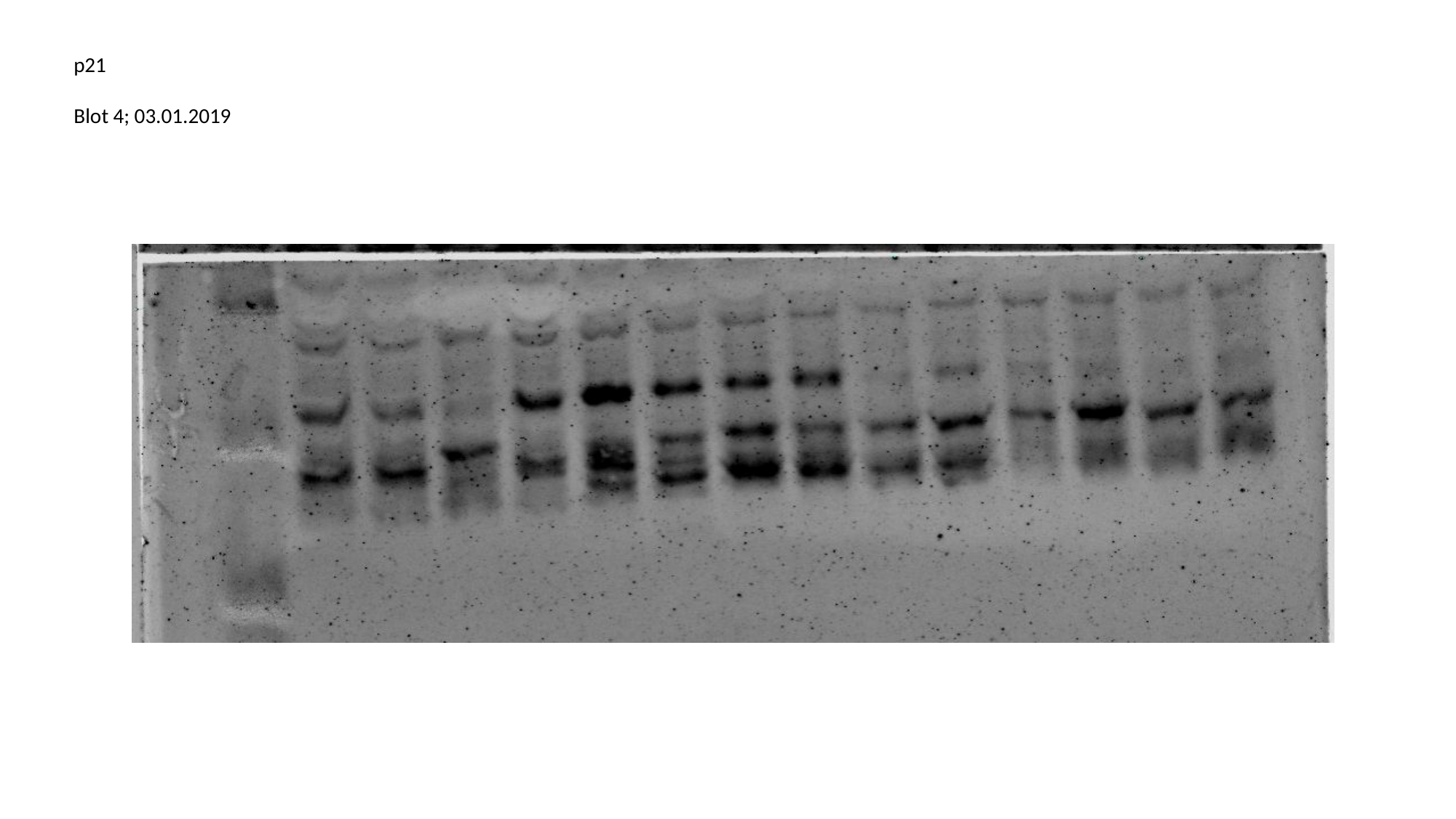

p21
Blot 4; 03.01.2019

## Slide 46
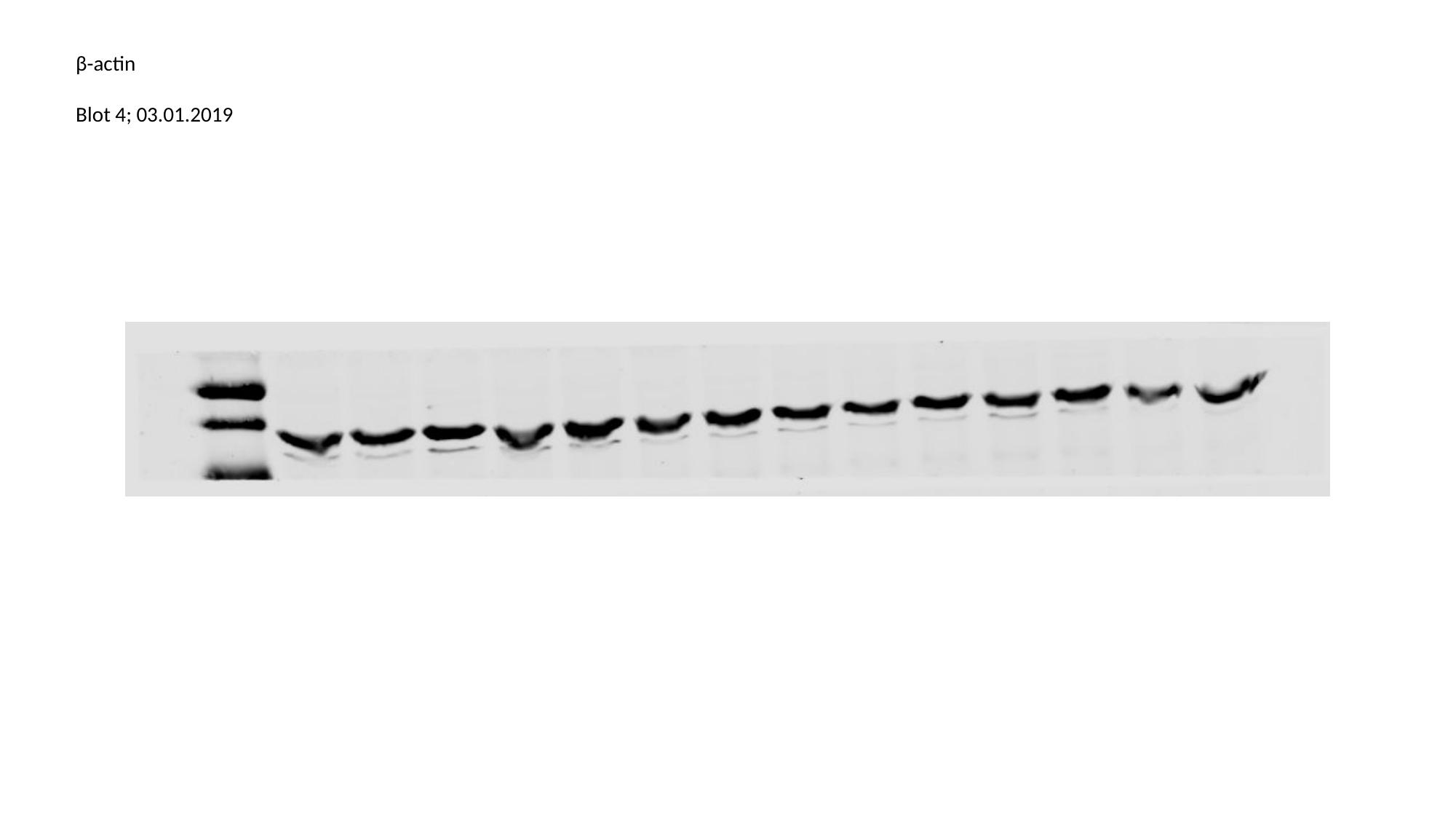

β-actin
Blot 4; 03.01.2019

## Slide 47
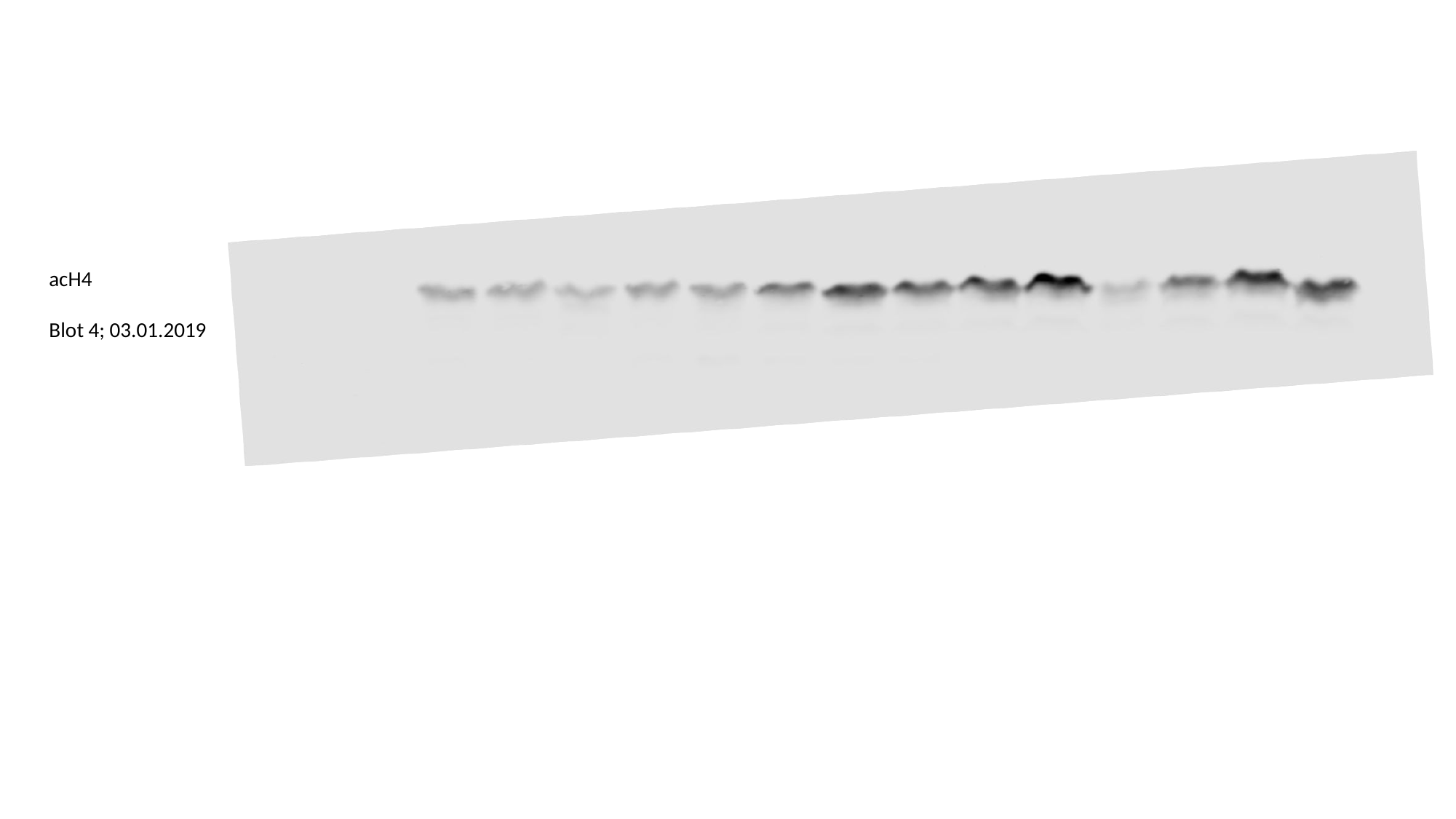

acH4
Blot 4; 03.01.2019

## Slide 48
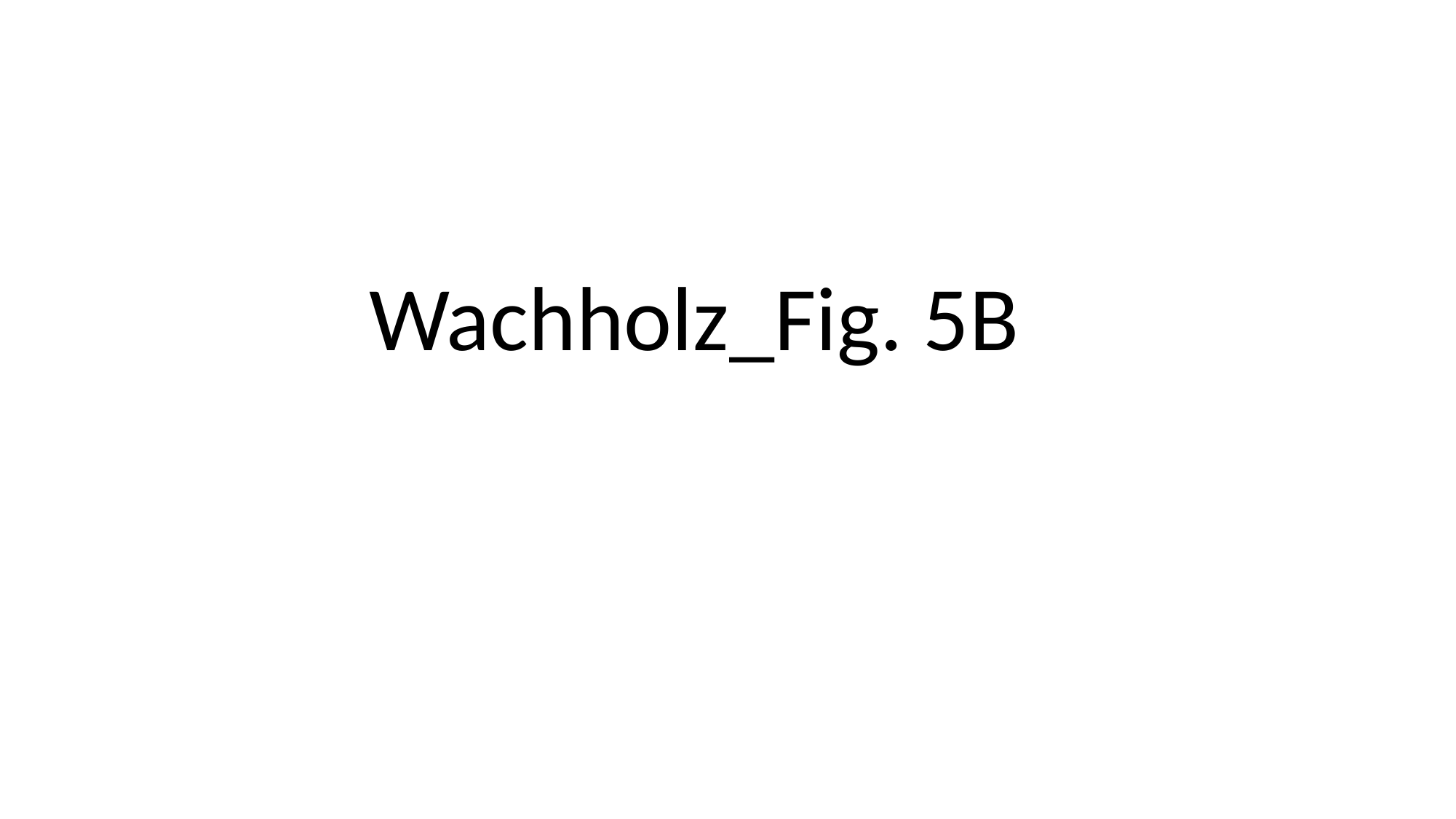

Wachholz_Fig. 5B

## Slide 49
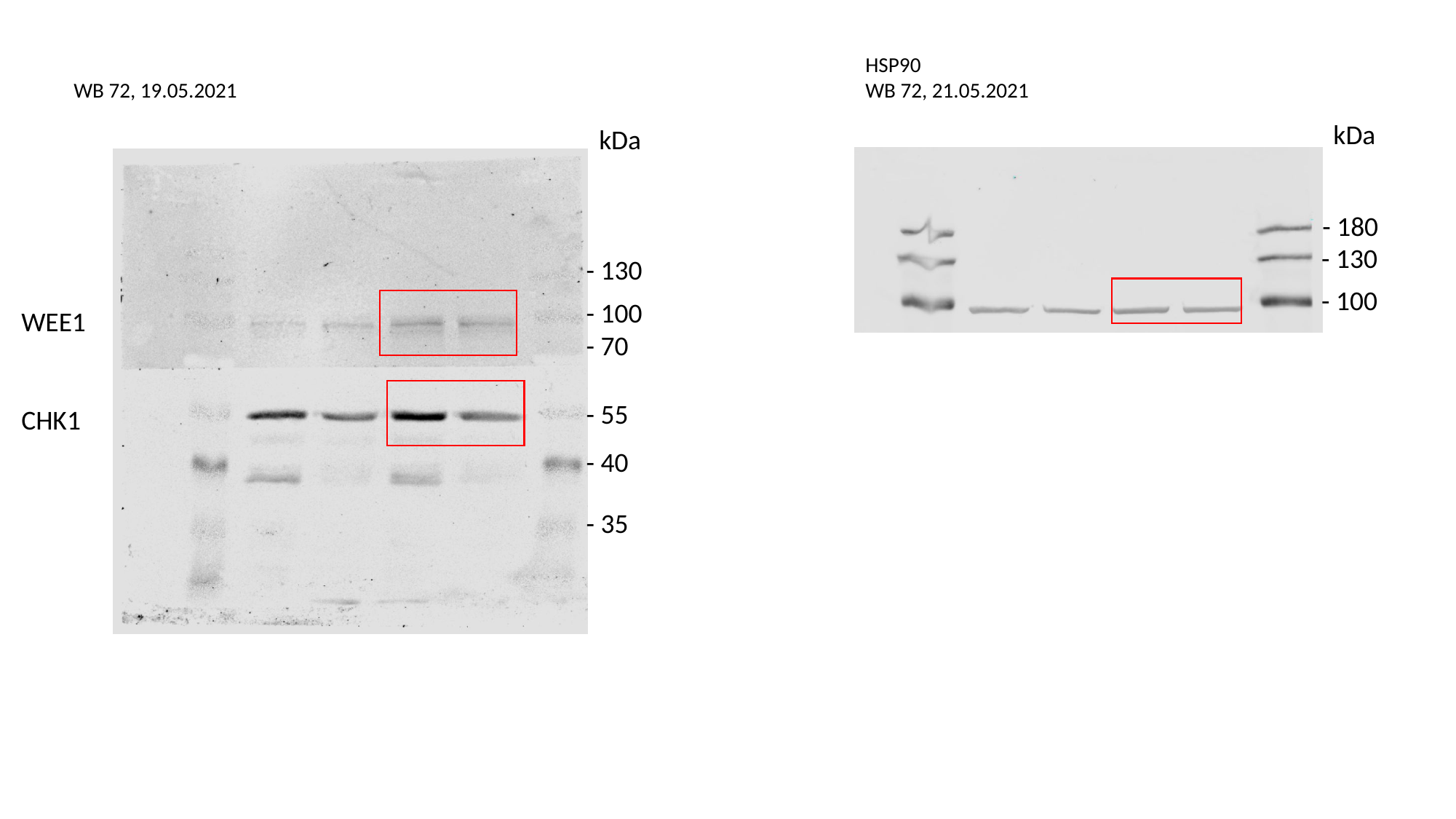

WB 72, 19.05.2021
HSP90
WB 72, 21.05.2021
kDa
kDa
- 180
- 130
- 130
- 100
- 100
WEE1
CHK1
- 70
- 55
- 40
- 35

## Slide 50
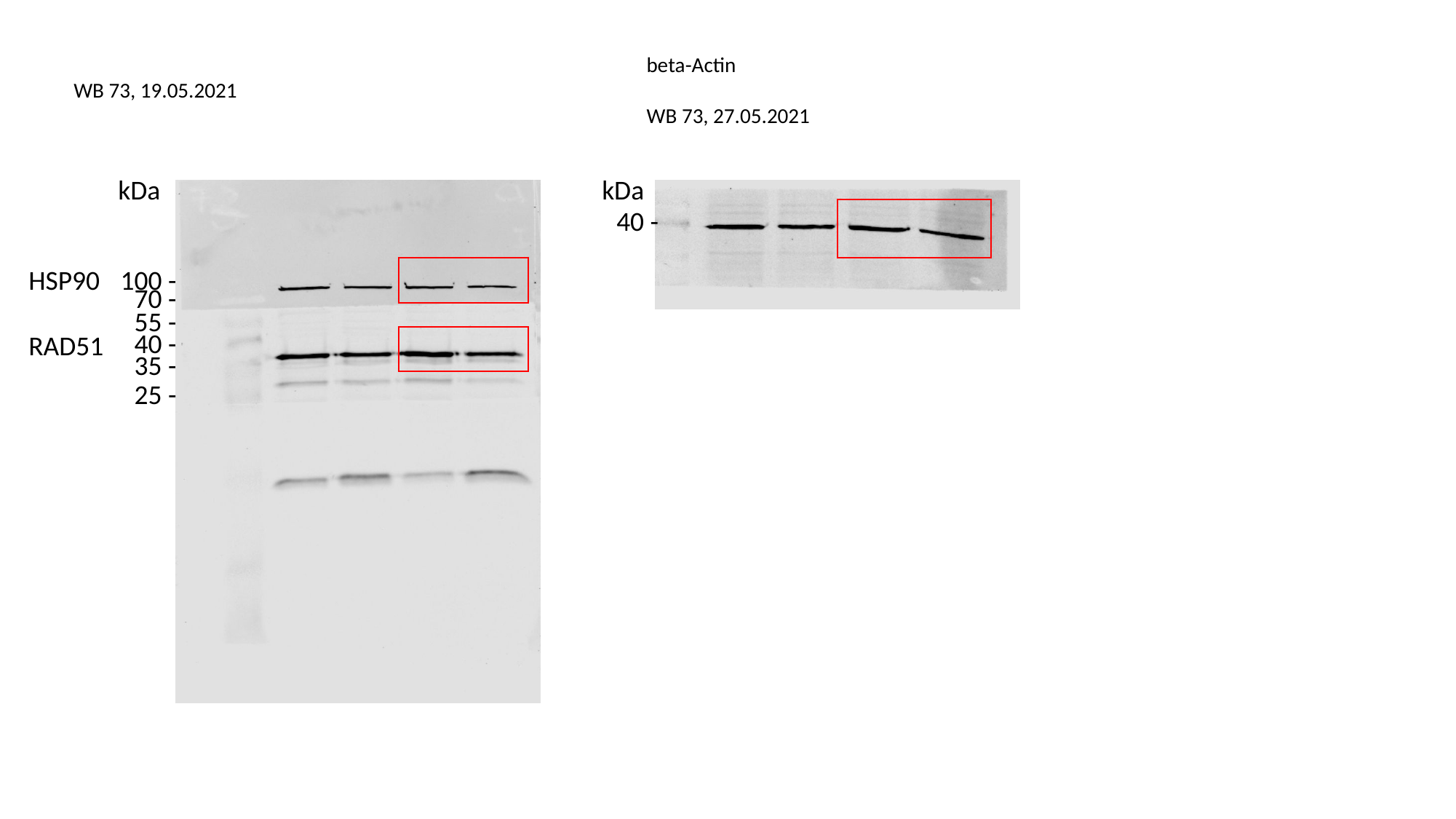

WB 73, 19.05.2021
beta-Actin
WB 73, 27.05.2021
kDa
kDa
 40 -
 100 -
HSP90
RAD51
 70 -
 55 -
 40 -
 35 -
 25 -

## Slide 51
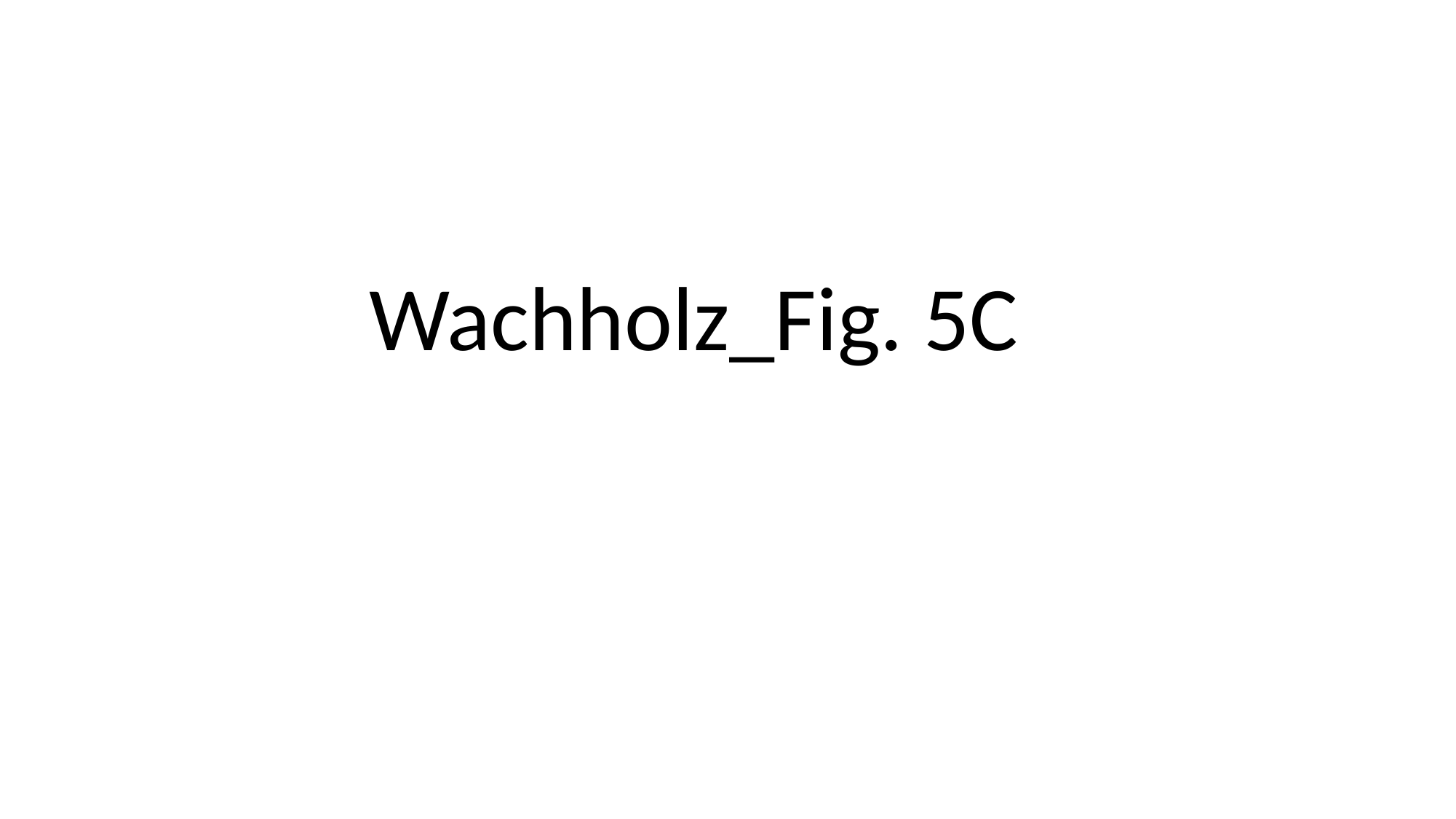

Wachholz_Fig. 5C

## Slide 52
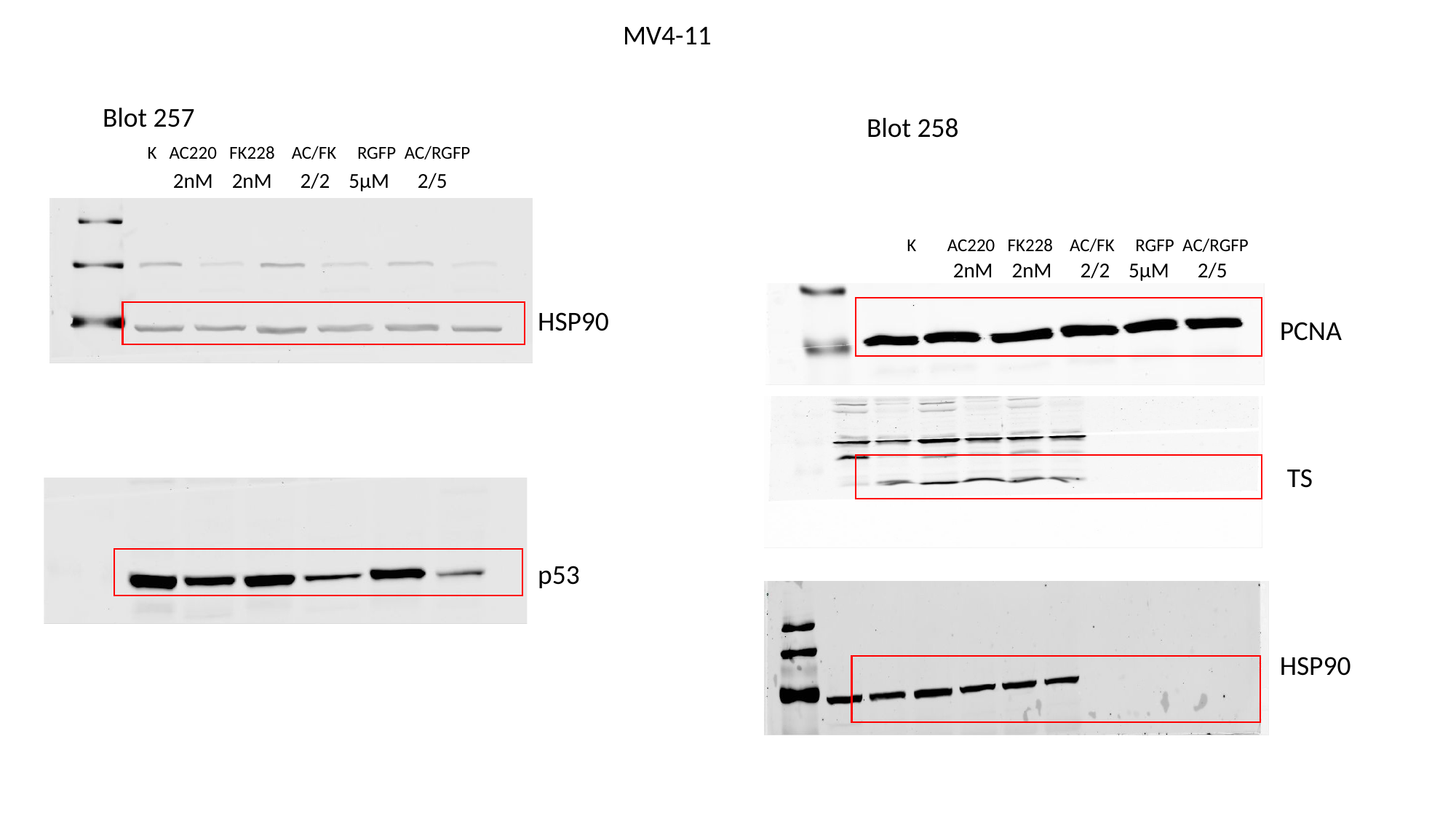

MV4-11
Blot 257
Blot 258
K AC220 FK228 AC/FK RGFP AC/RGFP
2nM 2nM 2/2 5µM 2/5
K AC220 FK228 AC/FK RGFP AC/RGFP
2nM 2nM 2/2 5µM 2/5
HSP90
PCNA
TS
p53
HSP90
